# Synthesis, Structure and Antileishmanial Evaluation of Endoperoxide–Pyrazole Hybrids

**DOI:** 10.3390/molecules27175401

**Published:** 2022-08-24

**Authors:** Patrícia S. M. Amado, Inês C. C. Costa, José A. Paixão, Ricardo F. Mendes, Sofia Cortes, Maria L. S. Cristiano

**Affiliations:** 1 Center of Marine Sciences, CCMAR, Gambelas Campus, University of Algarve, UAlg, 8005-139 Faro, Portugal; 2 Department of Chemistry and Pharmacy, Faculty of Sciences and Technology, FCT, Gambelas Campus, University of Algarve, UAlg, 8005-139 Faro, Portugal; 3 CFisUC, Department of Physics, University of Coimbra, 3004-516 Coimbra, Portugal; 4 CICECO-Aveiro Institute of Materials, University of Aveiro, 3810-193 Aveiro, Portugal; 5 Global Health and Tropical Medicine-GHTM, Instituto de Higiene e Medicina Tropical-IHMT, Universidade Nova de Lisboa-NOVA, 1349-008 Lisboa, Portugal

**Keywords:** antileishmanial chemotherapy, 1,2,4-trioxanes, 1,2,4,5-tetraoxanes, pyrazoles, endoperoxide–pyrazole hybrids, prototropic tautomerism

## Abstract

Leishmaniases are among the most impacting neglected tropical diseases. In attempts to repurpose antimalarial drugs or candidates, it was found that selected 1,2,4-trioxanes, 1,2,4,5-tetraoxanes, and pyrazole-containing chemotypes demonstrated activity against *Leishmania* parasites. This study reports the synthesis and structure of trioxolane–pyrazole (**OZ1**, **OZ2**) and tetraoxane–pyrazole (**T1**, **T2**) hybrids obtained from the reaction of 3(5)-aminopyrazole with endoperoxide-containing building blocks. Interestingly, only the endocyclic amine of 3(5)-aminopyrazole was found to act as nucleophile for amide coupling. However, the fate of the reaction was influenced by prototropic tautomerism of the pyrazole heterocycle, yielding 3- and 5-aminopyrazole containing hybrids which were characterized by different techniques, including X-ray crystallography. The compounds were evaluated for *in vitro* antileishmanial activity against promastigotes of *L. tropica* and *L. infantum*, and for cytotoxicity against THP-1 cells. Selected compounds were also evaluated against intramacrophage amastigote forms of *L. infantum.* Trioxolane–pyrazole hybrids **OZ1** and **OZ2** exhibited some activity against *Leishmania* promastigotes, while tetraoxane–pyrazole hybrids proved inactive, most likely due to solubility issues. Eight salt forms, specifically tosylate, mesylate, and hydrochloride salts, were then prepared to improve the solubility of the corresponding peroxide hybrids and were uniformly tested. Biological evaluations in promastigotes showed that the compound **OZ1•HCl** was the most active against both strains of *Leishmania*. Such finding was corroborated by the results obtained in assessments of the *L. infantum* amastigote susceptibility. It is noteworthy that the salt forms of the endoperoxide–pyrazole hybrids displayed a broader spectrum of action, showing activity in both strains of *Leishmania*. Our preliminary biological findings encourage further optimization of peroxide–pyrazole hybrids to identify a promising antileishmanial lead.

## 1. Introduction

Leishmaniases are a set of vector-borne infectious syndromes classified as neglected tropical diseases. Infections are caused by intracellular protozoans belonging to the genus *Leishmania*, from which more than 20 infective species are known worldwide [1,2,3]. Epidemiological data estimate 1.5 to 2 million new cases annually, with visceral leishmaniasis (VL) accounting for 500,000 cases and cutaneous leishmaniasis (CL) for 1 to 1.5 million cases [4]. Antileishmanial chemotherapy relies on a restricted arsenal of drugs comprising antimony-salts, paromomycin, miltefosine, and amphotericin B, all of which have serious shortcomings, such as unwanted side effects, unsatisfactory administration routes, and high cost and are also facing decreasing efficacy due to the emergence of resistances. These drawbacks prompted the search for better options to address leishmaniasis treatment [1,5].

**Figure 1:**
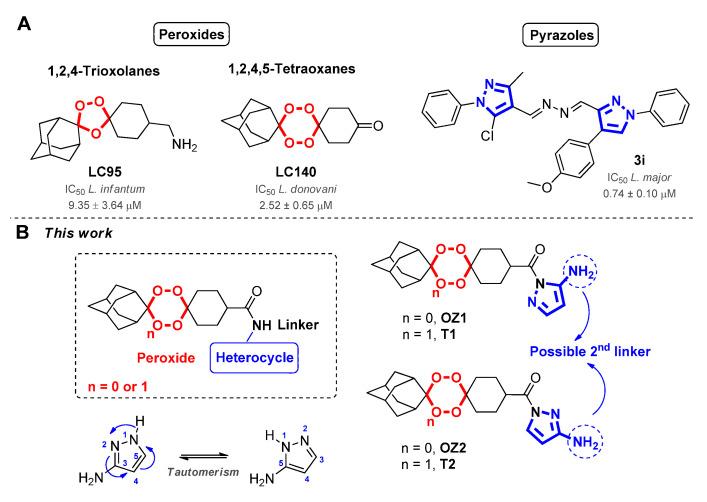
Structures of: (**A**) 1,2,4-trioxolane **LC95** [5], 1,2,4,5-tetraoxane **LC140** [1], and pyrazole derivative **3i** [17], with known antileishmanial activity; and (**B**) 1,2,4-trioxolane-3(5)-aminopyrazoles (**OZ1**, **OZ2**) and 1,2,4,5-tetraoxane-3(5)-aminopyrazoles (**T1**, **T2**) reported in this study.

Recently, it was demonstrated that selected synthetic 1,2,4,5-tetraoxanes and 1,2,4-trioxolanes exhibit antiparasitic activity against *Leishmania* spp. (Figure 1A) [1,5,6,7,8]. These endoperoxide classes have been extensively explored as an alternative to the natural compound artemisinin in the fight against malaria [9,10,11,12,13]. As for endoperoxides, the limelight of pyrazoles is prominent in the medicinal scope, extending their anti-inflammatory [14], antineoplastic [15], antiviral [16], and antileishmanial activity (Figure 1A) [17]. The pyrazole moiety exhibits prototropic tautomerism, leading to distinctive pyrazole-based structures with diverse reactivities [18]; so this class remains under intense scrutiny [19,20].

Bioactive endoperoxides can be coupled with other biologically active moieties, preferentially those acting through a different mechanism of action, to generate hybrids that integrate multiple pharmacophores in a single chemical entity, thus enabling the development of endoperoxide-based multi-target drugs [21]. This approach aims to increase effectiveness through a synergistic effect and to delay resistance selection. The linker between the pharmacophoric moieties can be tailored to adjust the drug’s pharmacokinetic performance and bioavailability [22,23]. Given the potential of endoperoxides and pyrazoles as tools against parasitic diseases, namely malaria and leishmaniases, we propose to explore endoperoxide–pyrazole hybrids. In this work, we disclose the synthesis and structure of 1,2,4,5-tetraoxane–pyrazole (**T1**, **T2**) and 1,2,4-trioxolane–pyrazole (**OZ1**, **OZ2**) hybrids (Figure 1B). The structures of the tetraoxane-based hybrids (5-amino-*1H*-pyrazol-1-yl)(dispiro[cyclohexane-1,3′-[1,2,4,5]tetraoxane-6′,2″-tricyclo[3.3.1.1^3,7^]decan]-4-yl)methanone (T1) and (3-amino-*1H*-pyrazol-1-yl)(dispiro[cyclohexane-1,3′-[1,2,4,5]tetraoxane-6′,2″-tricyclo[3.3.1.1^3,7^]decan]-4-yl)methanone (T2) were elucidated by X-ray crystallography. We conducted studies to evaluate the activity of the hybrids against *Leishmania infantum*, responsible for both CL and VL, and *Leishmania tropica,* responsible for CL [24,25], to explore novel antiparasitic candidates. By synthesizing and fully characterizing these novel compounds, we intended to explore their activity against protozoan parasites using *Leishmania* spp. as first models.

## 2. Results and Discussion

### 2.1. Synthesis and Structure Analysis

**Scheme 1:**
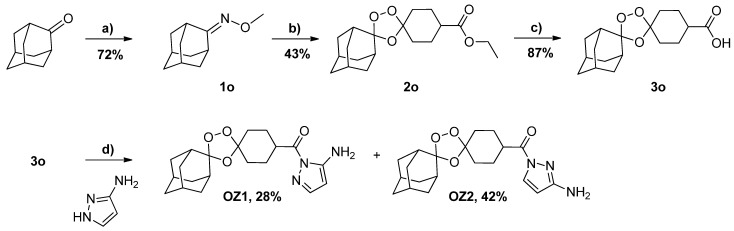
Reagents and conditions: (**a**) pyridine, MeONH_2_, MeOH, r.t, 72 h; (**b**) ethyl 4-oxocyclohexanecarboxylate, O_3_, DCM/Pentane, 0 °C, 2 h; (**c**) LiOH, THF/H_2_O, rt, o/n; (**d**) EDC.HCl, HOBt, Et_3_N, CH_2_Cl_2_, rt, o/n.

**Scheme 2:**
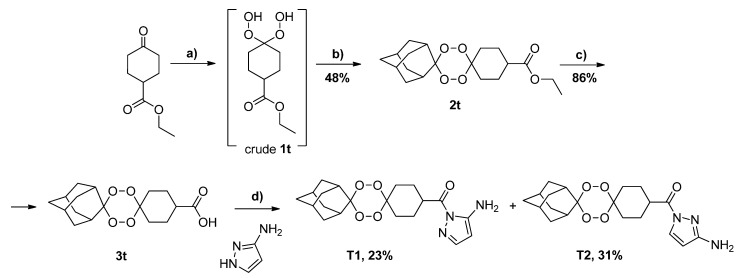
Reagents and conditions: (**a**) H_2_SO_4_-SiO_2_, H_2_O_2_ (50% *w*/*w*), CH_3_CN, 0º C-rt, 1 h; (**b**) adamantan-2-one, H_2_SO_4_-SiO_2_, CH_2_Cl_2_, 0ª C-r.t., 3 h; (**c**) LiOH, THF/H_2_O, rt, o/n; (**d**) EDC.HCl, HOBt, Et_3_N, CH_2_Cl_2_, rt, o/n.

The novel 1,2,4-trioxolane–pyrazoles **OZ1/OZ2** and 1,2,4,5-tetraoxane–pyrazoles **T1/T2** were synthesized by adapting procedures described in the literature, and their complete structure elucidation was achieved by ^1^H, ^13^C{^1^H}, 2D NMR, X-ray crystallography, and HRMS. The synthetic strategies adopted for both classes are depicted in Scheme 1 and Scheme 2. The detailed procedures for their preparation and characterization are described in the Section 3 and Supporting Information (SI). The main difference between these syntheses resides in the method used to produce the endoperoxide pharmacophore. Formation of the 1,2,4-trioxolane ring relies on a Griesbaum coozonolysis, using adamantan-2-one *O*-methyloxime **1o**, 4-oxocyclohexanecarboxylate, and ozone to yield the expected 1,2,4-trioxolane intermediate **2o**. The adamantan-2-one *O*-methyl-oxime **1o** employed in this procedure was previously synthesized from the reaction of adamantan-2-one with *O*-methylhydroxylamine in pyridine and methanol (Scheme 1). The method described by Amado et al. [26] was followed for the preparation of the precursor 1,2,4,5-tetraoxane **2t**, starting with the peroxyacetalization of ethyl 4-oxocyclohexanecarboxylate with 50% (*w*/*w*) aqueous hydrogen peroxide in acetonitrile in the presence of the silica sulfuric acid catalyst (SSA) to afford the crude dihydroperoxide (DHP, **1t**). Cyclocondensation of crude DHP **1t** with adamantan-2-one catalysed by SSA produced the desired 1,2,4,5-tetraoxane **2t** (Scheme 2). Hydrolysis of trioxolane-ester **2o** or tetraoxane-ester **2t** with lithium hydroxide in THF/H_2_O afforded the corresponding carboxylic acids, trioxolane **3o** or tetraoxane **3t** (Scheme 1 and Scheme 2). Endoperoxide carboxylic acids (**3o** or **3t**) were then reacted with 3-aminopyrazole under an inert atmosphere in the presence of 1-ethyl-3-(3-(dimethylamino)propyl)carbodiimide hydrochloride (EDC·HCl) and hydroxybenzotriazole (HOBt), as coupling agents, and triethylamine, to yield a mixture of 1,2,4-trioxolane–pyrazole or 1,2,4,5-tetraoxane–pyrazole positional isomers (**OZ1** and **OZ2** or **T1** and **T2**, Scheme 1 and Scheme 2).

The structures of both trioxolane and tetraoxane isomers were primarily elucidated by 1D and 2D Nuclear Magnetic Resonance (NMR), through ^1^H−^1^H correlation spectroscopy (COSY), heteronuclear single-quantum coherence (HSQC), and heteronuclear multiple-bond correlation (HMBC) NMR studies [Table 1 and Table 2 and Figures S11–S37 (SI)].

Due to conformational inversion, broad ^13^C signals were observed for the dispiro rings in 1,2,4,5-tetraoxanes in contrast to the sharper signals observed for the relatively inflexible 1,2,4-trioxolanes. Generally, in the tetraoxane dispiro system, the ^13^C{^1^H} peaks expand due to conformational flipping between the dispiro-cyclohexyl rings, resulting in the broadening of the peaks and a reduction in the number of carbons associated with those respective rings’ carbons. These observations are more frequent in six-membered ring endoperoxides, such as 1,2,4,5-tetraoxanes or 1,2,4-trioxanes [27]. Compared to the tetraoxane analogues, dispiro 1,2,4-trioxolanes exhibit much improved signal resolution and sharpness and a higher signal number in the non-aromatic region. As expected, a strong correlation coefficient was obtained in the ^1^H−^1^H COSY and HMBC spectra for endoperoxide containing dispiro rings. Furthermore, the combination of the ^1^H−^1^H COSY and HMBC spectra allowed for the complete identification of the pyrazole moiety’s carbons, enabling a more precise identification of the multiple -NH protons. Regarding the pyrazole ring, in the ^1^H−^1^H COSY spectrum, ^3^*J* correlations between H4 with H5 were observed, together with two long-range couplings with one of the -NH proton (δ = 5.54 ppm), including a four-bond coupling (^4^*J*) with H4 and a five-bond coupling (^5^*J*) with H5 in both isomers **OZ1**/**T1** (Figures S14 and S25, SI). HMBC ^3^*J*_C-NH_ correlations between each -NH (δ = 5.19 and 5.54 ppm) with C4 demonstrated that both isomers **OZ1** and **T1** have two signals in C4, implying the presence of two non-equivalent -NH protons. No HMBC ^3^*J*_C-NH_ correlation was observed between the carbonyl carbon and the -NH from the pyrazole ring, suggesting that the exocyclic amino group did not participate in the amide generation in isomers **OZ1**/**T1** (Figures S16 and S27, SI). These correlations indicate that a prototropic annular tautomerism [18,28] occurred during the coupling reaction, where isomers **OZ1**/**T1** are the products from reaction of the peroxide carboxylic acids with the 5-aminopyrazole tautomer. Protons -NH are described as non-equivalent, implying a strong intramolecular hydrogen bond involving one of the exocyclic -NH protons. 

For isomers **OZ2** and **T2**, a few ^1^H−^1^H COSY correlations on the pyrazole ring are shown, with a ^3^*J* correlation between H4 and H5 (Figures S19 and S31, SI). A five-bond coupling (^5^*J*) correlation is observed between H5 and -NH_2_ in isomer **T2** (Figure S31, SI). Regarding HMBC spectrum, a single signal ^3^*J*_C-NH_ in C3-NH_2_ to C4 is observed, which demonstrates that both -NH protons are equivalent. A weak ^2^*J*_C-H_ correlation in C3-NH_2_ to C3 was also observed in isomer **T2** (Figure S33, SI). Notably, the data demonstrate the presence of the conjugate in its 3-aminopyrazole-linked tautomeric form, which is identifiable in the crystal phase. Theoretically, the frame of 3(5)-aminopyrazole is also susceptible to side-chain tautomerism, as it has a proton-exchangeable amine side chain [18,28], which can be observed in this situation.

### 2.2. X-ray Crystal Analysis

A successful preparation of the single crystals of both tetraoxane–pyrazole hybrids enabled us to further confirm the different isomers through X-ray crystallography. 

**Figure 2:**
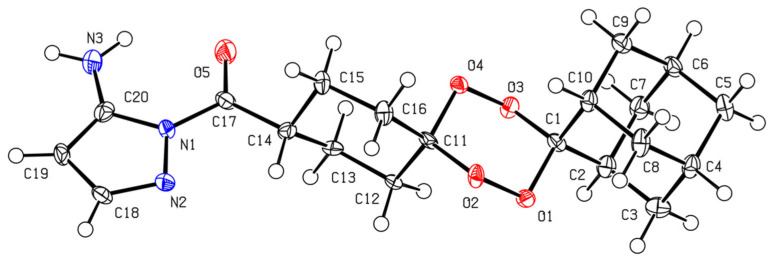
ORTEP drawing of the molecule of (5-amino-1H-pyrazol-1-yl) (dispiro[cyclohexane-1,3′-[1,2,4,5]tetraoxane-6′,2″-tricyclo[3.3.1.1^3,7^]decan]-4-yl)-methanone (**T1**). Displacement ellipsoids are drawn at the 50% probability level.

**Figure 3:**
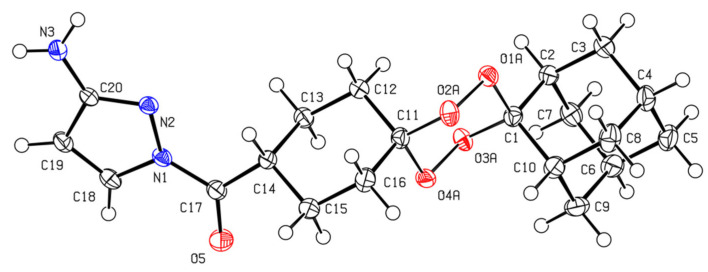
ORTEP drawing of the molecule of (3-amino-1H-pyrazol-1-yl)-(dispiro[cyclohexane-1,3′-[1,2,4,5]tetraoxane-6′,2″-tricyclo[3.3.1.1^3,7^]decan]-4-yl)-methanone (**T2**). Displacement ellipsoids are drawn at the 50% probability level. The tetraoxane group has minor disorder over two alternate chair conformations; for the sake of clarity, only the major conformation is shown.

The crystal of **T1** used for the data collection turned out to be a non-merohedral twin corresponding to a 180° rotation around the (100) reciprocal lattice axis; the twin-law (1 0 0.860; 0 −1 0; 0 0 −1) was found using the TwinRotMax routine implemented in PLATON [29]. The proportion of the two components of the twin was found to be 59%:41% via refinement of the BASF parameter. In compound **T2**, examination of the residual electron density disclosed that the tetraoxane group is slightly disordered (3%) over two alternate chair conformations, which was considered in the final refinement. The same amount of disorder was observed in the analysis of the dataset collected at room temperature. Oak Ridge Thermal Ellipsoid Plots (ORTEP) of the isomer molecules of **T1** and **T2** are depicted in Figure 2 and Figure 3, respectively. 

Bond lengths and valency angles are unexceptional, the average values in the tetraoxane ring being C–O (I: 1.429 Å; II: 1.430 Å), O–O, (I: 1.474 Å; II: 1.476 Å) and C–O–O (I: 107.8; II: 107.1°), respectively. In **T2,** the cyclohexane and tetraoxane rings adopt both an almost ideal chair form, with Cremer and Pople [30] puckering parameters *Q*: ~0.6 Å and pseudo-rotation angle *θ* < 2°. In the case of **T1**, the puckering amplitudes of these rings are similar to those of **T2**, and the conformation of the tetraoxane ring is also close to the ideal chair form, with *θ* = 1.7(6)°. However, the cyclohexane ring in **T1** features a significant distortion from the ideal chair conformation, with *θ* = 11.3(10)° For both isomers, the substituent at C14 is at an equatorial position to the cyclohexane ring. The conformation of the adamantane substituent is close to the ideal boat–boat form of the forming eight-membered rings, with approximate C*_s_* symmetry, in the two crystals. The pyrazole ring is planar within experimental error in both isomers. The C=O group is not strictly coplanar with the pyrazole ring, with a more pronounced rotation around the single N1–C17 bond in **T2**, as shown by the torsion angles O5–C17–N1–N2 (I: −175.9(8)°; II 167.90(12)°).

**Figure 4:**
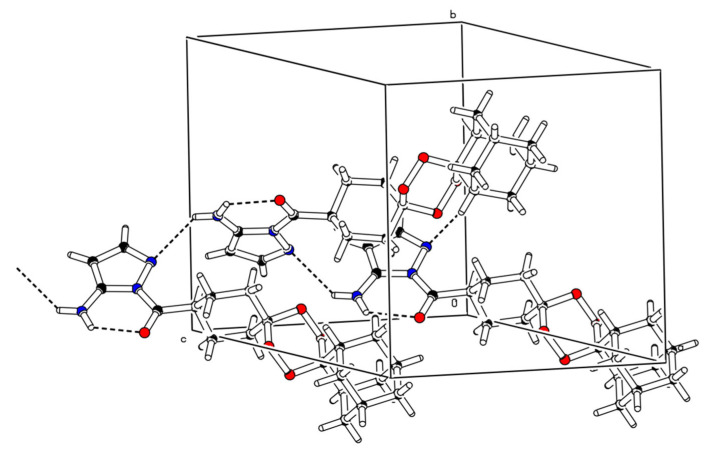
Hydrogen-bonding motif in the crystal structure of isomer **T1**.

**Figure 5:**
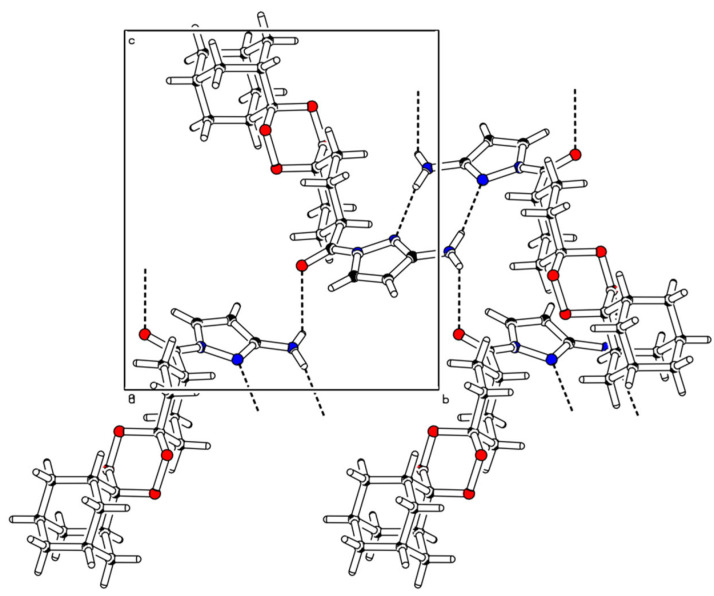
Hydrogen-bonding motif in the crystal structure of isomer **T2** (projection along the crystallographic a-axis).

In the crystal of **T1**, one of the two H atoms of the amino group forms a short intramolecular hydrogen bond with the bare O5 atom as acceptor (Table 3). The other H atom establishes a longer intermolecular hydrogen bond with the bare N atom of the pyrazole ring of a neighbor molecule, this hydrogen bond linking the molecules in infinite chains propagating along the crystallographic *c*-axis (Figure 4). In the crystal structure of **T2**, both H atoms of the amino group are involved in intermolecular hydrogen bonds; one of them is an N–H^…^N bond linking molecules across inversion centers in the unit cell; the other one is directed towards atom O5, as in the crystal of **T1**. In **T2**, the two hydrogen bonds form an infinite 2D network of molecules lying in the (100) plane (Figure 5). In both crystals, a set of C–H^…^O and C–H^…^N short contacts can be spotted, as well as C–H^…^C_g_ interactions with the π electron cloud of the pyrazole ring, that contribute to the stabilization of the crystal structures.

### 2.3. In Vitro Susceptibility of Leishmania spp. to Endoperoxide–Pyrazole Hybrids 

We undertook a preliminary *in vitro* evaluation of the antileishmanial activity of the endoperoxide–pyrazole hybrids **OZ1**, **OZ2**, **T1**, **T2**, and 3-aminopyrazole (**PYR**) against promastigote forms of the viscerotropic species *L. infantum* and the dermotropic species *L. tropica.* Axenic *Leishmania* promastigotes were used as a model for the initial compound screening, since they are a more straightforward and economical model due to their ease of culture development, requiring small amounts of the tested molecule [31]. The half-inhibitory concentration (IC_50_) of each compound was determined upon 48 h incubation with each parasite strain (Table 4). As control, the standard antileishmanial drug amphotericin B (**AmB**) was used. Compounds cytotoxicity was assessed (CC_50_) on the monocytic THP-1 cell line upon exposure to each compound, and the ClogP and ClogS values were calculated (Table 4). Materials and methodologies applied in the biological evaluation of the compounds are described in the Section 3.

From the endoperoxide–pyrazole hybrids tested against *L. tropica* promastigotes, trioxolanes **OZ1** and **OZ2** displayed the lowest IC_50_ values (219 ± 25 µM and 161 ± 19 µM, respectively). Regarding the assessments performed on *L. infantum* promastigotes, this strain only revealed susceptibility towards **OZ2**, which exhibited an IC_50_ of 219 ± 44 µM, yet slightly higher than that obtained for the parent **LC67 (2o)** (123 ± 1 µM, Table 4). Since both ozonides have a similar value of ClogS, the lack of activity of **OZ1** in *L. infantum* could be related to steric effects. Disparity in response to standard CL treatments or experimental drugs has become a significant burden to disease management and variations in treatment outcomes are ascribed to a number of factors, including specific characteristics and susceptibility among *Leishmania* species [32]; *L. tropica* and *L. infantum* are phylogenetically and genetically distinct, which justifies the appearance of different clinical forms as well as variable pharmacological susceptibility among species [33]. The calculated partition coefficient (ClogP) values proved very similar among the endoperoxides studied (1.79–2.61), suggesting that other physical properties could explain the differences in activity.

Overall, both ozonides demonstrated some antileishmanial activity, though lower than disclosed from previous in vitro studies where selected endoperoxides revealed antileishmanial potency at low micromolar (<10 µM) concentrations [5,34]. In the present study, the evaluation of compounds **T1** and **T2** was limited by their low solubility in M199 and RPMI medium. Calculated intrinsic aqueous solubility (ClogS) values show similar values between trioxolanes (1.20–1.38) and tetraoxanes (1.18–1.21). However, the poor solubility of dispiro 4′’-substituted tetraoxanes may be associated to their structural symmetry [35]. A decrease in molecular symmetry has been correlated to better physicochemical properties, such as absorption and solubility, resulting in enhanced systemic exposure to a drug. So it is anticipated that 3′’-substituted analogues could overcome this solubility issue [35,36]. Therefore, future optimization to 3”-substituted analogues should be considered. Likewise, conversion of the compounds into water-soluble salts, as well as encapsulation of the endoperoxide structures in delivery systems such as liposomes, could represent alternative strategies to solve the solubility issue [37,38].

Cytotoxicity tests revealed toxic effects of the hybrids **OZ1** and **OZ2** on THP-1 cells, with low selectivity (SI values of <5 µM). We observed that the parent 3-aminopyrazole (**PYR**) is devoid of antileishmanial activity but exhibits similar toxicity to **OZ2**, indicating that the toxicity in **OZ1** and **OZ2** is likely due to the aminopyrazole moiety. Moreover, the efficacy of **PYR** in inhibiting THP-1 (202 ± 10 µM), a monocytic leukemia human-derived cell line, also indicates that it could be a promising framework in the design of compounds with antineoplastic potential.

### 2.4. Effects of Endoperoxide–Pyrazole Hybrids on L. tropica Promastigotes Morphology

To evaluate the effect promoted by endoperoxide–pyrazole hybrids on *Leishmania* morphology, which can impair its infection capacity, we performed a morphometric analysis with optical microscopy (1000× amplification) in *L. tropica* promastigotes treated for 48 h with 300 µM of **OZ1** and 200 µM of **OZ2** as these concentrations were close to the IC_50_ values obtained for this species (219 ± 25 and 161 ± 19 µM, respectively). 

**Figure 6:**
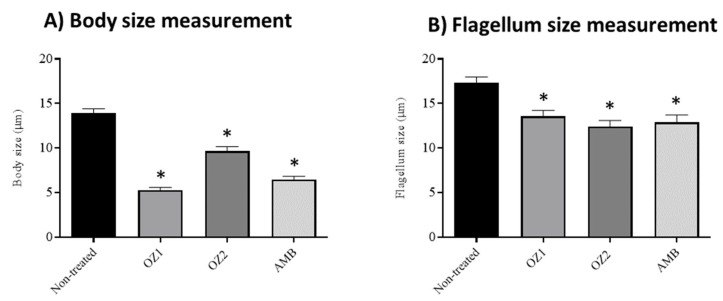
Body (**A**) and flagellum (**B**) measurements of *L. tropica* promastigotes after 48 h of treatment with OZ1 (300 µM), OZ2 (200 µM), and amphotericin B (0.62 µM). Non-treated parasites were also included in this evaluation. Significant differences between compounds and amphotericin B in comparison with non-treated parasites are indicated as *, *p* < 0.001 (ANOVA).

Figure 6 illustrates the measurements of *L. tropica* body (A) and flagellum (B) of parasites treated with both compounds and the control drug amphotericin B in comparison with non-treated parasites. Results show significant differences *p* < 0.001 for both compounds. Despite exhibiting a higher IC_50_ than **OZ2**, compound **OZ1** induced a greater reduction in body size (5.265 ± 1.733 µm) than **OZ2** (9.646 ± 2.744 µm), even slightly more pronounced than amphotericin B (6.463 ± 1.874 µm). On the other hand, both molecules decreased the flagellum size, with effects within the same range for **OZ2** (12.404 ± 3.745 µm) and **OZ1** (13.561 ± 3.535 µm). In general, changes in flagellum size are less evident when compared to body size loss; nevertheless, they may limit mobility and its capacity in infecting cells.

**Figure 7:**
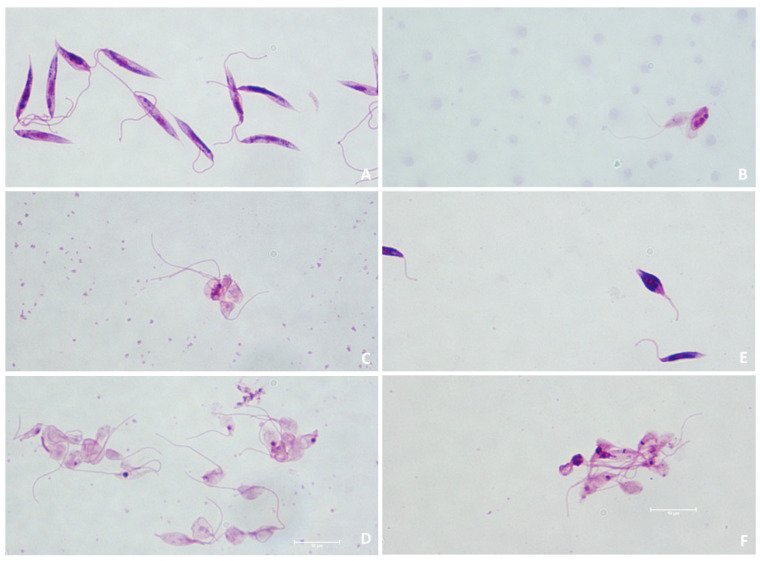
Assessment of *L. tropica* promastigotes morphology after 48- hours of treatment with 300 µM of **OZ1** (**C**,**D**), 200 µM of **OZ2** (**E**,**F**), and 0.625 µM of amphotericin B (**B**); in comparison with non-treated parasites (**A**). Figures (**C**–**F**) show the damage caused by both ozonides to the parasite, including body size reduction with an unnaturally rounded form, cytoplasmic disaggregation, derangement of internal organelles, and cell shrinkage. The observations were conducted on an inverted microscope (Eclipse 80i Nikon), and images were captured with a Nikon DS-Ri1 camera (magnification ×100).

Representative images of cellular alterations are depicted in Figure 7C–F. Non-treated cells and amphotericin B-treated cells were used as negative and positive controls, respectively (Figure 7A,B).

After 48 h of incubation, non-treated promastigotes (Figure 7A) exhibited a fusiform-shaped body, well-defined intracellular organelles, and an elongated flagellum. Contrarywise, in cultures treated with **OZ1** (Figure 7C,D) and **OZ2** (Figure 7E,F), most promastigotes showed considerable damage, namely significant body size reduction with an abnormally rounded shape, cytoplasmic disintegration, apparent disruption of internal organelles, and cell shrinkage, which are the typical alterations observed upon treatment with established antileishmanial compounds [39]. Another parallel impact of these compounds is the flagellum length reduction as discussed previously, which may cause impairments in motility. As expected, all these constraints were also observed in promastigotes treated with amphotericin B (Figure 7B).

Even though the IC_50_ values indicated that the ozonides have some activity, microscopic analysis revealed that *L. tropica* promastigotes experienced cell changes analogous to those caused by amphotericin B. These findings could support further investigations on the endoperoxide–pyrazole hybrids in the frame of leishmaniases chemotherapy, underlining the need for structure–activity studies and further optimisation.

### 2.5. Evaluation of the Hybrids’ Stability upon Conversion to Their Salt Forms

**Scheme 3:**
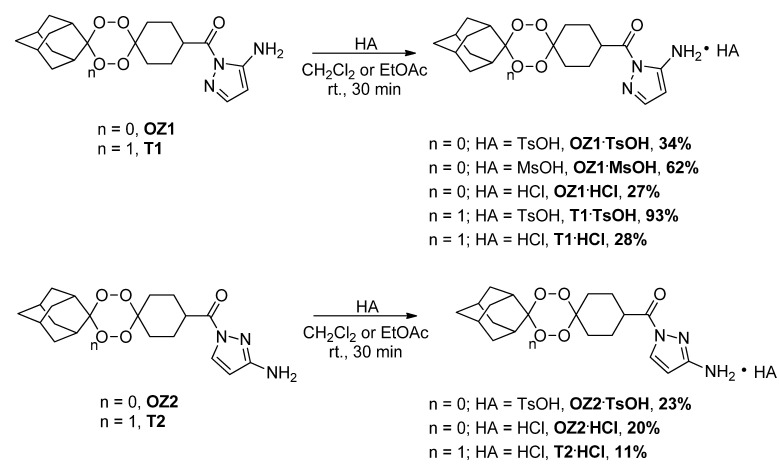
Preparation of different salt forms from the endoperoxide–pyrazole hybrids.

The antileishmanial activity of the hybrids on promastigotes of *L. tropica* revealed the need for structural modifications to improve the antileishmanial effect of these compounds. Because solubility may play a significant role in the antileishmanial activity of this series of molecules, we converted the free amines attached to the pyrazole moiety into their salt forms. Several acids, namely *p*-toluenesulfonic, methanesulfonic, and hydrochloric acid, can generate the free amines into salts with variable solubility levels. As such, the endoperoxide–pyrazole hybrids **OZ1, OZ2**, **T1** and **T2** were converted into their *p*-tosylate (**OZ1•TsOH, OZ2•TsOH**, and **T1•TsOH**), mesylate (**OZ1•MsOH**), and hydrochloride (**OZ1•HCl**, **OZ2•HCl**, **T1•HCl**, and **T2•HCl**) salts, with low to good yields (Scheme 3). Interestingly, this study has shown that the endoperoxide–pyrazole hybrids are stable even in strongly acidic conditions without decomposition of the endoperoxide ring, allowing further structure optimization.

Tests of antileishmanial activity were conducted on promastigote forms of *L. tropica* and *L. infantum,* then also on intracellular amastigote forms of *L. infantum* to ascertain the effect of the endoperoxide–pyrazole hybrids in their salt forms. The results are depicted in Table 5. The different salts displayed different antileishmanial efficacy depending on the conjugate salt, with IC_50_ values ranging from 135– > 400 µM against *L. tropica*, and from 164– > 400 µM against *L. infantum*. Among the salt forms of endoperoxide–pyrazole hybrids tested against *L. tropica* promastigotes the trioxolane **OZ1•HCl** displayed an IC_50_ value of 135 ± 36 µM, indicating that different salts of the same isomer exhibit divergent antileishmanial activities, with the **OZ1•HCl** salt proving to be the most active salt against *L. tropica*. Unlike for the results obtained for **OZ1, OZ2, T1** and **T2**, the assessments performed on *L. infantum* promastigotes revealed susceptibility of this strain towards most of the different salts except for the **T1** analog with TsOH and HCl. As observed for *L. tropica*, the trioxolane **OZ1•HCl** also displayed the lowest IC_50_ value (164 ± 20 µM) against *L. infantum*. Cytotoxicity assessments revealed toxic effects of all the salt derivatives on THP-1 cells, with CC_50_ values compressed in a concentration range of 331–529 µM. The improvements in solubility exhibited by the different salts enhanced the antileishmanial activity, although further structure–activity optimizations are still needed. However, it is worth noting that the salt forms of the endoperoxide–pyrazole hybrids display a broader spectrum of action, showing some effect on both strains of *Leishmania*. 

**Figure 8:**
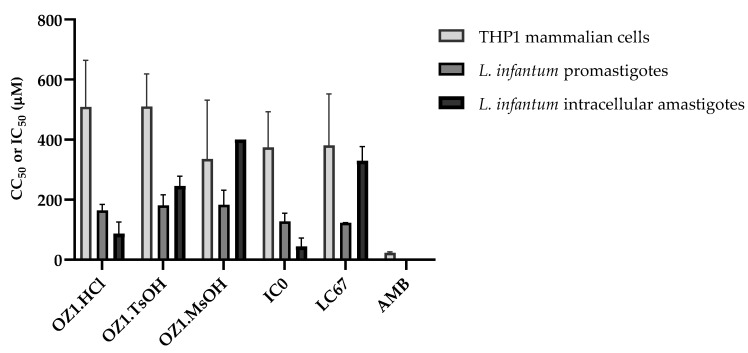
Graphic representation of cytotoxic (CC_50_) and inhibitory concentrations (IC_50_) for THP1 cell line, promastigotes, and amastigotes of *L. infantum*, respectively.

Compounds that exhibited an IC_50_ < 200 µM were also tested for intracellular *Leishmania* amastigotes susceptibility. The *L. infantum* strain was used as the model for this assay. According to the results of intracellular amastigote susceptibility (Table 4 and Table 5), **OZ1•HCl** and **IC0 (2t)** demonstrated superior activity compared to the other compounds, with lower IC_50_ values against intracellular amastigotes (87 ± 39 µM and 45 ± 28 µM) than in promastigotes (164 ± 20 µM and 128 ± 27 µM). In contrast, **OZ1•TsOH**, **OZ1•MsOH** and **LC67** evidenced a diminished effect on amastigotes compared to that shown on promastigotes (Figure 8). Possible reasons could be linked to bioaccumulation and mechanism of action for this class of compounds, including passage through the host cell’s cellular membrane or the formation of toxic metabolites toward *Leishmania* after metabolization of these compounds by host cell enzymes. As reported, the parasitophorous vacuole may serve as a barrier to direct interaction with the parasite, which was reported to eventually lead to a decrease in activity on moving from promastigote to amastigote stages [40]. Thus, it is noteworthy that **OZ1•HCl** and **IC0 (2t)** demonstrated superior activity compared to the other compounds, with slightly lower IC_50_ values against intracellular amastigotes than in promastigotes. Overall, the compounds tested against the amastigote forms displayed poor selectivity since SI values are smaller than 10.

## 3. Materials and Methods

### 3.1. Chemicals

All reagents for synthesis were purchased from commercial sources and used without further purification. Analytical thin layer chromatography (TLC) was carried out using Merck (Darmstadt, Germany) TLC Silica gel 60 F254 aluminum sheets and visualized under UV or by appropriate stain. *P*-Anisaldehyde and potassium permanganate were the most used. Column chromatography was carried out using Sigma Aldrich (Darmstadt, Germany) technical grade silica gel (pore size 60 Å, 230–400 mesh particle size, 40–63 μm particle size). 

### 3.2. Equipment

^1^H and ^13^C Nuclear Magnetic Resonance (NMR) spectra were recorded using a 400 MHz Bruker (Billerica, MA, USA) instrument or using a 500 MHz JEOL system (Peabody, MA, USA) equipped with a Royal HFX probe in the deuterated solvents described in each experimental procedure. The chemical shifts (δ) are described in parts per million (ppm) downfield from an internal standard of tetramethylsilane (TMS). Melting points (°C) were obtained on an SMP30 melting point apparatus and are uncorrected. High-Resolution Mass Spectrometry (HRMS) was recorded using the analytical service within the Centre of Marine Sciences (CCMAR, Algarve, Portugal) and was conducted on a Thermo Scientific High Resolution Mass Spectrometer (HRMS) (Waltham, MA, USA), model Orbitrap Elite, capable of MSn, n up to 10. X-ray diffraction data were collected on Bruker Kappa Apex II and D8 Quest diffractometers (Billerica, MA, USA) using graphite monochromated MoKα (λ = 0.71073 Å) radiation (see Section 3.4 for more details). 

Safety. Organic peroxides are potentially hazardous compounds (inflammable and explosive) and must be handled carefully: (1) a safety shield should be used for all reactions involving H_2_O_2_; (2) direct exposure to strong heat or light, mechanical shock, oxidizable organic materials, or transition-metal ions should be avoided.

### 3.3. Synthesis 

General Procedure 1: Removal of the ester group. Followed procedure by Jiricek et al. with slight modifications [41]. To a solution of peroxide-ester (1 mmol) in THF:H_2_O (1:1, 5 mL), LiOH (3 mmol) was added, and the reaction mixture was stirred until completion, at room temperature. THF was removed in vacuo, diluted with water, and acidified with 1M HCl. The aqueous layer was extracted with EtOAc (3 × 30 mL), washed with brine (2 × 30 mL), and dried with anhydrous MgSO_4_. The organic phase was then filtered and concentrated under reduced pressure. The solid obtained was then crushed and washed with cold n-hexane, giving the desired product.

General Procedure 2: Synthesis of the peroxide–pyrazole hybrids. Followed the procedure by Stec et al. [42] with slight modifications. Peroxide–carboxylic acid (1 mmol) was dissolved in anhydrous dichloromethane (DCM, 5 mL) at room temperature. To the solution, was added hydroxybenzotriazole (HOBt, 1.5 mmol) and 1-ethyl-3-(3-(dimethylamino)propyl)-carbodiimide hydro-chloride (EDC·HCl, 1.5 mmol) under a nitrogen atmosphere. After stirring for 1 h, 3-aminopyrazole (1.1 mmol) and triethylamine (2 mmol) were added, and the reaction mixture was stirred at room temperature until consumption of the starting material (usually overnight). To the resulting solution was added water, and the mixture was extracted with DCM (3 × 15 mL) and EtOAc (3 × 15 mL). The organic layers were separated, washed with brine, dried over anhydrous MgSO_4_, filtered, and concentrated under reduced pressure. The residue was purified by flash chromatography using a 0–40% EtOAc−hexane gradient (unless specified differently) to obtain the peroxide–pyrazole hybrids.

General Procedure 3: Synthesis of the peroxide-pyrazole *p*-tosylate salts. Procedure adapted from Vennerstrom et al. [43]. A solution of *p*-toluenesulfonic acid monohydrate (0.9 mmol) in ethanol (2.5 mL) was added to a solution of the endoperoxide–pyrazole hybrid (1 mmol) in CHCl_3_ (10 mL). The product formed was concentrated under reduced pressure and treated with diethyl ether (20 mL). The precipitate was then filtered, washed with diethyl ether (20 mL), and dried to yield the desired endoperoxide–pyrazole *p*-tosylate salt.

General Procedure 4: Synthesis of the peroxide–pyrazole mesylate salts. Procedure adapted from Vennerstrom et al. [43]. A solution of methanesulfonic acid (0.73 mmol) in diethyl ether (2.5 mL) was added to a solution of the endoperoxide–pyrazole hybrid (1 mmol) in CHCl_3_ (5 mL). The precipitate was then filtered, washed with diethyl ether (20 mL), and dried to yield the desired endoperoxide–pyrazole mesylate salt. 

General Procedure 5: Synthesis of the peroxide–pyrazole hydrochloride salts. Procedure adapted from Vennerstrom et al. [43]. Anhydrous HCl–Et_2_O (2.0 M, 10 mL was added to a solution of the endoperoxide–pyrazole hybrid (1 mmol) in CHCl_3_ (5 mL). The precipitate was then filtered, washed with diethyl ether (20 mL), and dried to yield the desired endoperoxide–pyrazole hydrochloride salt. 

*O-methyl-adamantan-2-one oxime (**1o**).* Followed procedure by Cabral et al. [1] with slight modifications. To a solution of adamantan-2-one (7.00 g, 46.6 mmol) in methanol (50 mL) were added pyridine (6.00 mL, 74.18 mmol) and methoxylamine hydrochloride (5.06 g, 60.52 mmol). The reaction mixture was stirred at room temperature for 48 h. The final mixture was concentrated and then diluted with DCM (50 mL) and water (50 mL). The organic layer was separated, and the aqueous layer was extracted with DCM (30 mL). The combined organic extracts were washed with aqueous HCl (1 M; 30 mL × 2), then with brine (30 mL). The final organic extract was dried over MgSO_4_, filtered, and concentrated under reduced pressure to give *O*-methyl-adamantan-2-one oxime (6.01 g, 72% yield) as a colourless solid. M.p. 69–70 °C. Spectral data are in accordance with those reported [1]. ^1^H NMR (400 MHz, CDCl_3_): δ 1.69–2.02 (m, 14H), 2.14 (t, *J* = 6.9 Hz, 4H), 2.51 (t, *J* = 7.0 Hz, 4H) ppm; ^13^C{^1^H} NMR (100 MHz, CDCl_3_): δ 25.9, 26.31, 31.09, 32.59, 34.25, 35.70, 36.18, 37.35, 106.46, 111.95, 208.90 ppm. HRMS (ES^+^, *m/z*) calcd C_11_H_18_NO (M+H)^+^: 180.13829; found 180.13783. Diff: 2.55 ppm.

*Ethyl dispiro[cyclohexane-1,3′-[1,2,4]trioxolane-5′,2″-tricyclo[3.3.1.1^3,7^]decane]-4-carboxylate (**2o**).* Followed procedure by Cabral et al. [1] with slight modifications. Ozone, produced with an ozone generator Sander Labor-Ozonizator 301.7 (0.5 L/min O_2_, 140 V), which was passed through a solution of dichloromethane at –78 °C and flushed into a solution of **1o** (2.00 g, 23.95 mmol) and ethyl 4-oxocyclohexanecarboxylate (1.89 mL, 11.97 mmol), in pentane/DCM (6:4, 100 mL) at 0 °C. After completion, the solution was flushed with nitrogen for 5 min and concentrated under reduced pressure at room temperature to give a crude material that was purified by column chromatography. (EtOAc: *n*-hexane, 1:99, *v/v*), affording a colourless oil (1.73 g, 48% yield). Spectral data are in accordance with those reported [1]. ^1^H NMR (500 MHz, CDCl_3_): δ 4.11 (dq, *J* = 9.4, 7.1 Hz, 2H), 2.40–2.26 (m, 1H), 2.00–1.66 (m, 22H), 1.24 (dt, *J* = 8.3, 7.1 Hz, 3H). ^13^C{^1^H} NMR (126 MHz, CDCl_3_): δ 175.04, 111.60, 108.04, 60.49, 41.52, 41.30, 37.15, 36.85, 36.44, 34.94, 34.90, 34.85, 34.83, 34.06, 33.66, 33.44, 33.29, 33.23, 31.59, 27.17, 27.02, 26.94, 26.53, 26.31, 26.13, 14.33, 14.30. HRMS (ES^+^, *m/z*) calcd C_19_H_32_NO_5_ (M+NH_4_)^+^: 354.22750; found 354.22702. Diff: 1.36 ppm.

*Ethyl dispiro[cyclohexane-1,3′-[1,2,4,5]tetraoxane-6′,2″-tricyclo[3.3.1.1^3,7^]decane]-4-carboxylate (**2t**).* Procedure adapted from Amado et al. [26]. Ethyl 4-oxocyclohexanecarboxylate (2 mL, 12.55 mmol) was dissolved in acetonitrile (38 mL), and silica sulfuric acid (SSA, 3.42 g, 2 mmol) was added to the mixture. Hydrogen peroxide 50 *wt*.% in H_2_O (4.09 mL, 4 mmol) was slowly added over an ice bath. Then the mixture was left to stir at room temperature until consumption of the starting material. To this mixture was added distilled water. Then the catalyst was filtered and rinsed with DCM. The filtrate was extracted with DCM (3 × 30 mL), dried over with MgSO_4_, and concentrated under reduced pressure at low temperature (30–35 °C) to obtain the *gem*-dihydroperoxide semi-crude, which was used immediately without further purification. The *gem*-dihydroperoxide semi-crude (**1t**) was dissolved in anhydrous DCM (75 mL) followed by addition of 2-adamantanone (5.66 g, 1.5 mmol). The mixture was cooled over an ice bath prior to addition of SSA (3.42 g, 2 mmol). The mixture was then warmed and left to stir at room temperature until consumption of the starting material. The resulting solution was then filtered, rinsed with DCM, and concentrated under reduced pressure. Purification through flash chromatography (EtOAc: *n*-hexane, 2.5:97.5, *v/v*) yielded a white solid (2.11 g, 48% yield). M.p. = 67–69 °C. Spectral data are in accordance with those reported [26]. ^1^H NMR (500 MHz, CDCl_3_): δ 4.12 (q, *J* = 7.1 Hz, 2H), 3.02 (d, *J* = 118.6 Hz, 2H), 2.41–2.34 (m, 1H), 2.08–1.60 (m, 19H), 1.50 (s, 1H), 1.23 (t, *J* = 7.1 Hz, 3H). ^13^C{^1^H} NMR (126 MHz, CDCl_3_): δ 174.75, 110.61, 107.28, 60.53, 41.77, 39.35, 37.01, 34.35, 33.21, 30.18, 30.16, 28.29, 27.11, 24.82, 23.94, 14.31. HRMS (ESI^+^, *m/z*) calcd C_19_H_28_O_6_Na (M+Na)^+^: 375.17781; found 375.17725. Diff: 1.49 ppm.

*Dispiro[cyclohexane-1,3′-[1,2,4]trioxolane-5′,2″-tricyclo[3.3.1.1^3,7^]decane]-4-carboxylic acid (**3o***). This compound was synthesized in accordance with general procedure 1, using **2o**. White solid (2.01 g, 88% yield). M.p. = 148–150 °C. Spectral data are in accordance with those reported [44]. ^1^H NMR (500 MHz, CDCl_3_): δ 2.43–2.33 (m, 1H), 2.00–1.67 (m, 22H). ^13^C{^1^H} NMR (126 MHz, CDCl_3_): δ 181.06, 180.94, 111.93, 111.68, 107.93, 107.88, 41.06, 40.85, 39.36, 36.84, 36.43, 34.95, 34.89, 34.85, 34.82, 33.30, 33.06, 27.17, 26.93, 26.52, 26.05, 25.88. HRMS (ESI^−^, *m/z*) calcd C_17_H_23_O_5_ (M−H)^−^: 307.15510; found 307.15488. Diff: 0.72 ppm.

*Dispiro[cyclohexane-1,3′-[1,2,4,5]tetraoxane-6′,2″-tricyclo[3.3.1.1^3,7^]decane]-4-carboxylic acid (**3t**).* This compound was synthesized in accordance with general procedure 1, using **2t**. White solid (1.96 g, 86% yield). M.p. = 179–182 °C. Spectral data are in accordance with those reported [1]. ^1^H NMR (500 MHz, CDCl_3_): δ 3.14 (s, 1H), 2.91 (s, 1H), 2.49–2.42 (m, 1H), 2.06–1.72 (m, 17H), 1.62–1.44 (m, 3H). ^13^C{^1^H} NMR (126 MHz, CDCl_3_): δ 180.57, 110.66, 107.12, 41.32, 39.36, 37.01, 34.33, 33.30, 33.21, 32.31, 30.26, 28.21, 27.12, 24.58, 23.68. HRMS (ESI^−^, *m/z*) calcd C_17_H_23_O_6_ (M−H)^−^: 323.15001; found 323.14975. Diff: 0.80 ppm.

*(5-Amino-1H-pyrazol-1-yl)(dispiro[cyclohexane-1,3′-[1,2,4]trioxolane-5′,2″-tricyclo-[3.3.1.1^3,7^]-decan]-4-yl)methanone (**OZ1**) and (3-Amino-1H-pyrazol-1-yl)(dispiro[cyclohexane-1,3′-[1,2,4]trioxolane-5′,2″-tricyclo[3.3.1.1^3,7^]decan]-4-yl)methanone (**OZ2**).* The compounds were prepared in accordance with general procedure 2, through amide coupling of **3o** with 3(5)-aminopyrazole. Purification by flash chromatography (EtOAc: *n*-hexane, 20:80, *v/v*) yielded the two isomers as white solids (**OZ1:** 0.81 g, 28% yield; **OZ2:** 1.22 g, 42%).

**OZ1**: M.p. = 153–155 °C. TLC *R*_f_: 0.41 (EtOAc: *n*-hexane, 20:80, *v/v*). ^1^H NMR (500 MHz, CDCl_3_): δ 7.37 (d, *J* = 1.8 Hz, 1H), 5.54 (s, 2H), 5.37 (dd, *J* = 4.6, 1.8 Hz, 1H), 3.59 (dtq, *J* = 11.7, 9.3, 3.6 Hz, 1H), 1.87 (m, 22H). ^13^C{^1^H} NMR (126 MHz, CDCl_3_): δ 178.50, 150.35, 144.01, 111.64, 108.00, 88.93, 40.86, 36.87, 36.47, 34.87, 34.83, 33.46, 26.95, 26.55, 26.34. HRMS (ES^+^, *m/z*) calcd C_20_H_28_N_3_O_4_ (M+H)^+^: 374.20743; found 374.20706. Diff: 0.99 ppm. 

**OZ2**: M.p. = 64–66 °C. TLC *R*_f_: 0.23 (EtOAc: *n*-hexane, 20:80, *v/v*). ^1^H NMR (500 MHz, CDCl_3_): δ 7.99 (d, J = 3.0 Hz, 1H), 5.88 (d, *J* = 3.0 Hz, 1H), 3.99 (s, 2H), 3.45–3.36 (m, 1H), 2.06–1.71 (m, 22H). ^13^C{^1^H} NMR (126 MHz, CDCl_3_): δ 173.12, 157.34, 130.13, 130.09, 111.98, 111.58, 108.06, 108.03, 101.03, 100.97, 39.64, 36.87, 36.47, 36.45, 34.95, 34.89, 34.87, 34.83, 33.51, 33.43, 26.96, 26.55, 26.45, 26.36. HRMS (ES^+^, *m/z*) calcd C_20_H_28_N_3_O_4_ (M+H)^+^: 374.20743; found 374.20752. Diff: -0.24 ppm.

*(5-Amino-1H-pyrazol-1-yl)(dispiro[cyclohexane-1,3′-[1,2,4,5]tetraoxane-6′,2″-tricyclo-[3.3.1.1^3,7^]-decan]-4-yl)methanone (**T1**) and (3-Amino-1H-pyrazol-1-yl)(dispiro[cyclohexane-1,3′-[1,2,4,5]tetraoxane-6′,2″-tricyclo[3.3.1.1^3,7^]decan]-4-yl)methanone (**T2**).* The compounds were synthesised in accordance with general procedure 2, through amide coupling of **3t** with 3(5)-aminopyrazole. Purification by flash chromatography (EtOAc: *n*-hexane, 20:80, *v/v*) yielded the two isomers as white solids (**T1**: 0.35 g, 23% yield; **T2**: 0.49 g, 31%).

**T1:** M.p. = 187–189 °C (dec.). TLC *R*_f_: 0.40 (EtOAc: *n*-hexane, 20:80, *v/v*). ^1^H NMR (500 MHz, CDCl_3_): δ 7.36 (d, *J* = 1.8 Hz, 1H), 5.54 (s, 2H), 5.37 (d, *J* = 1.8 Hz, 1H), 3.66 (ddt, *J* = 11.2, 7.5, 3.8 Hz, 1H), 3.17 (s, 2H), 2.11–1.57 (m, 22H). ^13^C{^1^H} NMR (126 MHz, CDCl_3_): δ 178.17, 150.34, 144.04, 110.65, 107.22, 88.95, 41.32, 37.02, 34.05, 33.23, 30.24, 28.40, 27.13, 24.85, 24.44. HRMS (ES^+^, *m/z*) calcd C_20_H_28_N_3_O_5_ (M+H)^+^: 390.20235; found 390.20181. Diff: 1.38 ppm.

**T2**: M.p. = 171–173 °C (dec.). TLC *R_f_*: 0.22 (EtOAc: *n*-hexane, 20:80, *v/v*). ^1^H NMR (500 MHz, CDCl_3_): δ 8.03–7.97 (d, *J* = 2.9 Hz, 1H), 5.90–5.87 (d, *J* = 3.0 Hz, 1H), 4.02–3.89 (s, 2H), 3.54–3.45 (tt, *J* = 11.1, 3.8 Hz, 1H), 3.28–3.07 (s, 2H), 2.05–1.61 (m, 20H). ^13^C{^1^H} NMR (126 MHz, CDCl_3_): δ 178.17, 150.34, 144.04, 110.65, 107.22, 88.95, 41.32, 37.02, 34.05, 33.23, 30.24, 28.40, 27.13, 24.85, 24.09. HRMS (ES^+^, *m/z*) calcd C_20_H_28_N_3_O_5_ (M+H)^+^: 390.20235; found 390.20206. Diff: 0.74 ppm.

*(5-Amino-1H-pyrazol-1-yl)(dispiro[cyclohexane-1,3′-[1,2,4]trioxolane-5′,2″-tricyclo-[3.3.1.1^3,7^]-decan]-4-yl)methanone p-tosylate (**OZ1•TsOH).*** The compound was prepared from **OZ1,** in accordance with general procedure 3. White solid (87 mg, 34% yield). M.p. = 149 °C (dec.). ^1^H NMR (500 MHz, DMSO-*d*_6_): δ 7.51–7.43 (d, *J* = 8.0 Hz, 2H), 7.44–7.37 (d, *J* = 1.8 Hz, 1H), 7.21–7.06 (d, *J* = 7.8 Hz, 2H), 5.34–5.27 (d, *J* = 1.8 Hz, 1H), 3.57–3.44 (ddt, *J* = 11.8, 7.1, 3.7 Hz, 1H), 2.32–2.25 (s, 3H), 2.05–1.40 (m, 22H) ppm. ^13^C{^1^H} NMR (126 MHz, DMSO-*d*_6_): δ 177.47, 151.10, 145.59, 144.28, 137.78, 128.15, 125.54, 110.83, 107.87, 87.33, 40.11, 40.02, 36.10, 35.78, 34.28, 32.78, 26.23, 25.97, 25.83, 20.83 ppm.

*(5-Amino-1H-pyrazol-1-yl) (dispiro[cyclohexane-1,3′-[1,2,4]trioxolane-5′,2″-tricyclo-[3.3.1.1^3,7^]-decan]-4-yl) methanone mesylate (**OZ1•MsOH).*** The compound was prepared in accordance with general procedure 4, using **OZ1.** White solid (110 mg, 62% yield). M.p. = 118 °C (dec.). ^1^H NMR (500 MHz, DMSO-*d*_6_): δ 7.43–7.38 (d, *J* = 1.8 Hz, 1H), 5.34–5.28 (d, *J* = 1.8 Hz, 1H), 3.59–3.51 (tt, *J* = 11.7, 3.4 Hz, 1H), 2.41–2.38 (s, 3H), 1.95–1.53 (m, 22H) ppm. ^13^C{^1^H} NMR (126 MHz, DMSO-*d*_6_): δ 177.47, 151.10, 144.28, 110.84, 107.88, 87.34, 40.11, 40.02, 36.11, 35.78, 34.29, 32.78, 26.24, 25.98, 25.84 ppm.

*(5-Amino-1H-pyrazol-1-yl) (dispiro[cyclohexane-1,3′-[1,2,4]trioxolane-5′,2″-tricyclo-[3.3.1.1^3,7^]-decan]-4-yl) methanone hydrochloride (**OZ1•HCl).*** The compound was prepared in accordance with general procedure 5, using **OZ1.** White solid (53 mg, 27% yield). ^1^H NMR (500 MHz, DMSO-*d*_6_): δ 7.41 (d, *J* = 1.8 Hz, 1H), 5.31 (dd, *J* = 3.2, 1.8 Hz, 1H), 3.55 (tt, *J* = 11.7, 3.4 Hz, 1H), 1.96–1.53 (m, 24H) ppm. ^13^C{^1^H} NMR (126 MHz, DMSO-*d*_6_): δ 177.46, 151.09, 144.26, 110.82, 107.87, 87.32, 36.09, 35.77, 34.44, 34.27, 32.77, 32.71, 26.22, 25.96, 25.82 ppm. 

*(3-Amino-1H-pyrazol-1-yl) (dispiro[cyclohexane-1,3′-[1,2,4]trioxolane-5′,2″-tricyclo-[3.3.1.1^3,7^] decan]-4-yl) methanone p-tosylate (**OZ2•TsOH**).* The compound was synthesised in accordance with general procedure 3, using **OZ2**. White solid (89 mg, 23% yield). M.p. = 129 °C (dec.). ^1^H NMR (500 MHz, DMSO-*d*_6_): δ 8.03 (d, *J* = 3.0 Hz, 1H), 7.48 (d, *J* = 8.1 Hz, 2H), 7.12 (d, *J* = 7.8 Hz, 2H), 5.94 (d, *J* = 3.0 Hz, 1H), 3.41–3.33 (m, 1H), 2.29 (s, 3H), 1.94–1.54 (m, 22H) ppm. ^13^C{^1^H} NMR (126 MHz, DMSO-*d*_6_): δ 173.16, 158.43, 145.61, 137.77, 129.61, 128.15, 125.54, 110.85, 107.85, 102.12, 38.61, 36.10, 35.77, 34.28, 32.84, 26.23, 26.09, 25.83, 20.83 ppm.

*(3-Amino-1H-pyrazol-1-yl) (dispiro[cyclohexane-1,3′-[1,2,4]trioxolane-5′,2″-tricyclo-[3.3.1.1^3,7^] decan]-4-yl) methanone hydrochloride (**OZ2•HCl**).* The compound was synthesised in accordance with general procedure 5, using **OZ2** (38 mg, 20% yield). ^1^H NMR (500 MHz, DMSO-*d*_6_): δ 7.89 (d, *J* = 2.6 Hz, 1H), 5.98 (dd, *J* = 3.0, 1.1 Hz, 1H), 3.47–3.22 (m, 1H), 1.89–1.66 (m, 24H) ppm. ^13^C{^1^H} NMR (126 MHz, DMSO-*d*_6_): δ 176.01, 158.40, 129.57, 110.69, 107.98, 102.15, 36.10, 35.77, 34.41, 34.30, 34.27, 32.84, 32.75, 32.60, 26.23, 26.08, 25.91, 25.83 ppm. 

*(5-Amino-1H-pyrazol-1-yl) (dispiro[cyclohexane-1,3′-[1,2,4,5] tetraoxane-6′,2″-tricyclo-[3.3.1.1^3,7^]-decan]-4-yl) methanone p-tosylate **(T1•TsOH).*** The compound was synthesised in accordance with general procedure 3, using **T1**. White solid (60 mg, 93% yield). M.p. = 156 °C (dec.). ^1^H NMR (500 MHz, DMSO-*d*_6_): δ 7.44 (d, *J* = 7.7 Hz, 2H), 7.37 (s, 1H), 7.08 (d, *J* = 7.7 Hz, 2H), 5.28 (s, 1H), 3.60–3.55 (m, 1H), 3.11–2.93 (m, 2H), 2.25 (s, 3H), 1.86–1.52 (dt, *J* = 85.6, 25.7 Hz, 22H) ppm. ^13^C{^1^H} NMR (126 MHz, DMSO-*d*_6_): δ 177.79, 151.60, 146.09, 144.80, 138.25, 128.63, 126.03, 110.33, 107.55, 87.84, 40.98, 36.73, 34.13, 33.08, 30.14, 28.29, 26.91, 25.06, 21.32 ppm.

*(3-Amino-1H-pyrazol-1-yl) (dispiro[cyclohexane-1,3′-[1,2,4,5]tetraoxane-6′,2″-tricyclo [3.3.1.1^3,7^] decan]-4-yl) methanone hydrochloride **(T1•HCl).*** The compound was synthesised in accordance with general procedure 5, using **T1**. White solid (57 mg, 28% yield). ^1^H NMR (500 MHz, DMSO-*d*_6_): δ 7.41 (d, *J* = 1.8 Hz, 1H), 5.31 (d, *J* = 1.8 Hz, 1H), 3.61 (tt, *J* = 11.0, 3.7 Hz, 1H), 3.08 (d, *J* = 41.3 Hz, 2H), 1.86–1.64 (m, 22H) ppm. ^13^C{^1^H} NMR (126 MHz, DMSO-*d*_6_): δ 177.79, 151.60, 144.78, 110.33, 107.55, 87.85, 40.97, 36.73, 34.15, 33.08, 30.30, 29.98, 28.30, 26.91, 25.04, 24.08 ppm.

*(3-Amino-1H-pyrazol-1-yl) (dispiro[cyclohexane-1,3′-[1,2,4,5]tetraoxane-6′,2″-tricyclo [3.3.1.1^3,7^] decan]-4-yl) methanone hydrochloride **(T2•HCl).*** The compound was synthesised in accordance with general procedure 5, using **T2**. White solid (23 mg, 11% yield). ^1^H NMR (500 MHz, DMSO-*d*_6_): δ 7.97 (d, *J* = 2.9 Hz, 1H), 5.88 (d, *J* = 3.0 Hz, 1H), 5.63 (s, 2H), 3.47–3.42 (m, 1H), 2.99 (br d, *J* = 48.0 Hz, 3H), 1.78–1.59 (m, 19H) ppm. ^13^C{^1^H} NMR (126 MHz, DMSO-*d*_6_): δ 176.34, 159.46, 129.89, 110.33, 110.25, 107.54, 102.56, 36.73, 34.15, 33.08, 31.77, 30.35, 29.54, 28.63, 26.91, 25.15, 24.44 ppm. 

### 3.4. X-ray Crystallography

X-ray crystallographic studies were performed on single crystals of **T1** and **T2** at room temperature (292 K) for T2 and at low-temperature (150 K) for **T1** and **T2**, using Mo Kα radiation (λ = 0.71073 Å). The room temperature data were collected in a Bruker Kappa Apex II diffractometer equipped with a 4K CCD detector; the low temperature data were measured in a Bruker D8 Quest diffractometer equipped with a PHOTON II CMOS detector and an Oxford Cryostream 700 N_2_ flow cryostat. Both isomers crystallize in the monoclinic *P*2_1_/*c* space group (nº 14). 

Summary of crystal data:

**T1**: *a* =14.909(4) Å, *b*= 11.695(3) Å, *c* = 11.121(3) Å, β = 108.697(7)°, *V* = 1836.7(3) Å ^3^, *P*2_1_/*c*, *Z* = 4, *T* =150 (2) K, µ(Mo Kα) = 0.102 mm^−1^, *D*_calc_ = 1.419 g/cm^3^, 8603 reflections measured (2.261° < 2θ < 30.187°) 3212 unique (*R*_int_ = 0.0542, *R*_σ_ = 0.0520), 2917 with *I* > 2σ which were used in the calculations. The final *R*_1_ was 0.1159 (*I* > 2σ) and w*R* was 0.3672 (all data).

**T2**: *a* =12.9853(2) Å, *b*= 11.2578(2) Å, *c* = 11.1712(2) Å, β = 101.911(6)°, *V* = 1884.0(4) Å^3^, *P*2_1_/*c*, *Z* = 4, *T* =150 (2) K, µ(Mo Kα) = 0.099 mm^−1^, *D*_calc_ = 1.373 g/cm^3^, 66967 reflections measured (2.402° < 2θ < 27.499°) 4321 unique (*R*_int_ = 0.0124, *R*_σ_ = 0.0322), 4030 with *I* > 2σ which were used in the calculations. The final *R*_1_ was 0.0449 (*I* > 2σ) and w*R* was 0.1139 (all data).

The structures of **T1** and **T2** were solved by direct methods using SHELXT-2018/2 [45]. Full-matrix least-squares refinements on *F*^2^ were carried out with SHELXL-2018/3 [46] with anisotropic displacement parameters for all non-hydrogen atoms (CCDC 2151742 and 2151175 for detailed crystal data).

Hydrogen atoms were located on a difference Fourier synthesis, and their positions were refined as riding on parent atoms with an isotropic temperature constrained those of their parent atoms using SHELXL-2018/3 defaults [46], except those of the amino group that had their positions freely refined with an isotropic displacement factor constrained to 1.2× that of the parent N atom, for being involved in hydrogen bonding. 

### 3.5. Biological Evaluation

All compounds were solubilized in dimethyl sulfoxide (DMSO, Merck, Darmstadt, Germany). Working solutions for biological studies were prepared with a maximum of 1% DMSO. Amphotericin B (Gibco) was used as control drug.

#### 3.5.1. Parasites and Cell Lines

For the anti-*Leishmania* assay, promastigote forms of *L. infantum* (MHOM/PT/1988/IMT151) and *L. tropica* (MHOM/PS/2002/63jnF21) were maintained at 24 °C ± 1 °C in M199 medium (Sigma), supplemented with 10% (*v*/*v*) foetal bovine serum (FBS, Sigma, Darmstadt, Germany), 2,5 mg/mL hemin (Sigma), HEPES 1M pH7.4 (Sigma), and 1% (*v*/*v*) penicillin-streptomycin (Sigma). The viscerotropic *L. infantum* strain belongs to IHMT, Portugal cryobank collection, and the dermotropic *L. tropica* strain was provided by the Technical University of Applied Sciences Wildau, Germany (Dr. Katrin Kuhls). Promastigotes in culture had less than 10 in vitro passages, aiming to keep the strain virulence. Promastigotes were used for the experiments at the end of the log phase (days 5–6).

THP-1 (ATCC^®^ TIB-202TM) human acute monocytic leukemic cell line was obtained from DSMZ, Germany and cultured in RPMI 1640 (Sigma, Darmstadt, Germany) supplemented with 10% (*v*/*v*) FBS, 1% (*v*/*v*) penicillin-streptomycin (Sigma), and L-glutamine 200 mM (Sigma) at 37 °C, 5% CO_2_.

#### 3.5.2. *Leishmania* spp. Promastigotes In Vitro Susceptibility

The half maximal inhibitory concentration (IC_50_) was determined. *L. infantum* and *L. tropica* promastigotes were platted in 96-well flat bottom culture plates (VWR) at a density of 5 × 10^6^ parasites/mL in M199 medium with six different concentrations of each compound (OZ1: 400, 350, 300, 250, 200, and 150 µM; **OZ2**, **T1**, **T2** and PIR: 400, 200, 100, 50, 25, and 12.5 µM), including amphotericin B (10, 5, 2.5, 1.25, 0.625, and 0.313 µM). A blank (M199 medium alone) and an untreated control (parasites with M199 medium plus 1% DMSO as vehicle solution) were also included. All the compounds were plated out in quadruplicate and incubated for 48-h at 24 °C ± 1 °C. 

The parasite’s viability was determined using 3-(4,5-dimethyl-2-thiazolyl)-2,5-diphenyl-2H-tetrazolium bromide (MTT) colorimetric test. At the end of incubation time, 20 µL per well of MTT was added (5 mg/mL in PBS), followed by 2–4 h incubation at 37 °C ± 1 °C. After a centrifugation at 1000× *g* (20 min. 0 °C), medium was removed, and formazan crystals were solubilized in DMSO; optical density at 595 nm was measured with Synergy^TM^ HTX Multi Mode Microplate Reader (Dynex Technologies, Chantilly, VA, USA). GraphPad Prism V 9.0 was used to calculate IC_50_ of each chemical by fitting the data as a non-linear regression with variable slope using a dose–response inhibitory model. At least three independent assays were performed. 

To detect morphological alterations, at the end of the incubation period (48 h), 30 µL of the parasite’s suspension were collected from different treatment concentrations of each compound to prepare smears on glass slides, fixed with methanol, and stained with 5% (*v*/*v*) Giemsa (Sigma). Stained slides were examined with bright field in an inverted microscope (Eclipse 80i Nikon, Tokyo, Japan) with 1000× amplification, and images were captured with a Nikon DS-Ri1 camera. Image J (version 1.53k) was used to measure promastigote body and flagellum size of at least 30 parasite samples.

Data were expressed as mean ± standard error of the mean (SEM), and statistical analysis was performed with one-way ANOVA (Bonferoni’s multiple comparisons test) to determine whether differences between means relatively to untreated control were significant at different levels (*p* < 0.05, *p* < 0.01, *p* < 0.001).

#### 3.5.3. In Vitro Cytotoxicity Assessment in THP-1 Cell Line

THP-1 cell line was used to calculate the endoperoxide–pyrazole hybrids’ half-maximal cytotoxic concentration (CC_50_). THP-1 was plated at a final density of 5 × 10^5^ cells/mL, in 96-well flat bottom culture plates (VWR), using six different concentrations of the compounds (**OZ1**, **OZ2**, **T1**, **T2** and PIR: 800, 400, 200, 100, 50, 25 µM) and amphotericin B (10, 5, 2.5, 1.25, 0.625, 0.313 µM). A blank (RPMI medium alone) and an untreated control (parasites with RPMI medium plus 1% DMSO as vehicle solution) were also included. All the compounds were plated out in quadruplicate and incubated for 48 h at 37 °C ± 1 °C, 5% CO_2_.

Following the incubation period, 20 µL per well of MTT was added as described in Section 3.5.2. After absorbance readings, the CC_50_ values of each compound were determined with GraphPad Prism V 9.0 (Dotmatics, San Diego, CA, USA) as previously described. At least three independent assays were performed.

#### 3.5.4. *Leishmania* spp. Intracellular Amastigotes Susceptibility

The in vitro intracellular amastigote assay was performed using a THP1 cell line maintained in complete RPMI at 37 °C, 5% CO_2_. After 24 h differentiation of 5 × 10^5^ cells/mL into macrophages in sterile 16-chamber LabTek slides (Thermo Scientific, Waltham, MA, USA) with 25 ng mL^−1^ phorbol myristic acid (PMA, Sigma), cells were washed once with warm RPMI to remove non-differentiated and non-adherent cells and further infected with 5 × 10^6^ promastigotes/mL in a 10:1 parasite–macrophage ratio for another 24 h. After this period, slides were gently washed once with warm RPMI to remove non-internalised promastigotes. The compounds **2o**, **2t**, **OZ1•HCl**, **OZ1•MsOH**, and **OZ1•TsOH** at concentrations ranging 400 µM to 50 µM were added, and the slides were further incubated at 37 °C, 5% CO_2_ for 72 h. After this period, cells were washed with warm PBS, fixed with methanol (Sigma) and stained with Giemsa 5% (Sigma). AmB was used as positive control, and macrophages cultivated in RPMI medium and DMSO (0.5% *v*/*v*) were used as negative control. The infection index was determined by multiplying the percentage of infected cells and the number of amastigotes per infected cell as previously described [47]. The results represent the average of two experiments. The IC_50_ for each compound was calculated by fitting the data as a non-linear regression with variable slope using a dose–response inhibitory model in the GraphPad Prism V 8.0 (Dotmatics, San Diego, CA, USA) program.

Selectivity index (SI) was determined for each compound as the quotient between the cytotoxicity in cell lines (CC_50_) and the inhibitory concentration (IC_50_) (SI = CC_50_ in mammalian cells/IC_50_ *L. infantum*).

### 3.6. Software

Chemical structures were designed using ChemDraw Ultra 12.0 (Cambridge Soft, Cambridge, MA, USA). Graphics, measurements, and statistical analysis were performed using Microsoft Excel, ImageJ (version 1.53k, National Institutes of Health, Bethesda, Maryland, USA) and GraphPad Prism 9.0 (Dotmatics, San Diego, CA, USA). Bruker APEXIII [48] software package including SAINT V8.38A; [49] and SADABS-2016/2 [50] was used for data reduction of the X-ray crystallography work. SHELXT-2018/2 [45] and SHELXL-2018/3 [46] and PLATON (v. 2010420) [51] were used for the structure solution, refinement, and structure analysis and drawing of crystallographic figures, respectively.

## 4. Conclusions

This work describes, for the first time, the synthesis and structure of 1,2,4-trioxolane–pyrazole and 1,2,4,5-tetraoxane–pyrazole hybrids. Using a convergent synthetic approach, the hybrids were prepared by amide coupling of 3(5)-aminopyrazole with a carboxylic acid derivative of the endoperoxide-containing building block. The compounds were characterized in detail using 1D NMR (^1^H and ^13^C{^1^H}), 2D NMR (^1^H-^1^H COSY, HSQC, and HMBC), X-ray crystallography, and HRMS-ESI^+^. The ^1^H and ^13^C{^1^H} NMR spectroscopic and X-ray crystallography analyses enabled structural differentiation between 1,2,4-trioxolane and 1,2,4,5-tetraoxane isomers based on chemical shifts, signal sharpness, and number, whereas ^1^H-^1^H COSY and HMBC spectra allowed us to elucidate each positional isomer for both classes. X-ray crystallography unambiguously identified the structure of the two isomers and disclosed a distinct supramolecular organisation in the crystalline phase, despite both isomers crystallising in the same monoclinic space-group due to distinct hydrogen bonding patterns linking the molecules. Results reveal that only the endocyclic amine nitrogen of 3(5)-aminopyrazole participates in the amide coupling reactions with the carboxylic acid-containing endoperoxide building blocks. The reaction affords products from reaction of both 3- and 5-amino pyrazole tautomers, revealing the impact of prototropic tautomerism in the pyrazole heterocycle on reactivity.

Preliminary studies were conducted to evaluate the antileishmanial activity of this novel chemotype. Compounds **OZ1** and **OZ2** displayed better activity against *L. tropica* than the **T1** and **T2** compounds, which was attributed to the poor solubility of the tetraoxane hybrids in M199 and RPMI medium. Regarding the assessments performed in *L. infantum*, the results demonstrated no activity of all compounds toward this strain. The 3-aminopyrazole moiety was tested against both strains to determine the role of this moiety in the antileishmanial activity of all compounds and was found inactive. These results suggest that the antileishmanial activity depends on its substituents and could mean that the incorporation of a pyrazole scaffold into the endoperoxide structure may have no synergistic effect. In the future, and upon optimization, a broad range of *Leishmania* species and other protozoans, namely other trypanosomatids, should be tested in order to better understand their susceptibility and evaluate the potential of the novel chemotype. 

The endoperoxide–pyrazole hybrids were found to be very stable, as their salt forms could be successfully prepared without compromising the endoperoxide pharmacophore. This conversion to the salt form solved the insoluble nature of these compounds. Tests were conducted to ascertain the potential activity of these endoperoxide–pyrazole hybrids in their salt forms against promastigote and intracellular amastigote forms. Among all the salts tested against promastigotes of *L. tropica* and *L. infantum*, **OZ1•HCl** proved to be the best option. Our findings indicate that the conversion of the free amine in the respective salts not only improved the solubility but also amplified the spectrum of action of this chemotype.

The evaluation of *L. infantum* amastigotes susceptibility corroborates the findings on the antileishmanial activity in promastigotes in which **OZ1•HCl** was the compound with better activity of the new series. In addition, the compound with the lowest IC_50_ against *L. infantum* amastigotes was identified as **2t**, suggesting that the structure of these compounds must be simplified in order to enhance the effectiveness of new antileishmanial peroxides. Even though the issue of solubility has been addressed, it is not the primary factor influencing the antiparasitic effectiveness of this series. Considerable structure optimization was achieved with **OZ1•HCl** and **2t**. However, the results of 1,2,4-trioxolane–pyrazole and 1,2,4,5-tetraoxane–pyrazole hybrids highlight the need for further structure optimization to improve the activity of these compounds. 

## Figures and Tables

**Table 1:** Correlations found in the HSQC, and HMBC spectra of 1,2,4-trioxolane–pyrazole isomers **OZ1** and **OZ2**.

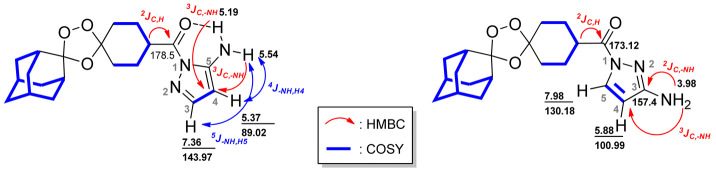							
Isomer OZ1				Isomer OZ2			
^1^H, δ (ppm)		^13^C, δ (ppm)		^1^H, δ (ppm)		^13^C, δ (ppm)	
	COSY	HSQC	HMBC		COSY	HSQC	HMBC
1.67	1.97	36.85	26.75, 35.05, 111.73	1.67	1.72, 1.80, 1.81	30.20, 36.85	26.75, 34.95, 111.49, 111.73
1.69	1.97	34.85	111.73	1.68	1.73	34.85	26.79, 111.49
1.72	2.00, 1.97	34.85	26.75	1.72	1.67	35.82	-
1.79	2.01, 2.02, 3.57	26.55	26.50	1.73	1.67, 1.68, 2.02	34.85	-
1.81	3.57	33.52	36.76, 108.07	1.75	1.97, 2.02	33.69	-
1.82	3.57	27.05, 26.22	33.32, 33.10, 40.91	1.77	1.67, 1.90, 1.96	26.55	26.53, 39.69
1.97	1.67, 1.69, 1.72, 3.57	36.51	36.76, 36.50, 111.73	1.80	1.67, 1.90	33.36	36.89, 39.70, 108.07
2.00	1.72, 3.57	34.85	34.81	1.81	1.67, 1.90	26.38	39.73
2.01	1.79, 3.57	26.38		1.82	1.72	27.05	26.50, 39.73
2.02	1.79, 3.57	33.52	26.26, 33.34, 40.91	1.90	1.81	34.85	26.58, 33.84, 37.02
3.57	1.79, 1.81, 1.82, 1.97, 2.00, 2.01	40.83	-	1.96	1.67, 1.77	26.38. 33.52	39.8, 108.07, 173.12
5.19	-	-NH	89.02	1.97	1.75, 1.77, 1.81	34.85, 36.51	26.53, 36.85, 111.49, 173.12
5.37	5.54, 7.36	88.02	143.97,150.32	2.02	1.73, 1.75	33.52	26.50, 33.40, 39.69, 108.07
5.54	5.37, 7.36	-NH	89.02	3.98	-	-NH_2_	100.99, 157.40
7.36	5.37, 5.54	143.97	89.02, 150.32	5.88	7.98	100.99	130.18, 157.40
-	-	-	-	7.98	5.88	130.18	100.99; 157.40

**Table 2:** Correlations found in the HSQC, and HMBC spectra of 1,2,4,5-tetraoxane–pyrazole isomers **T1** and **T2**.

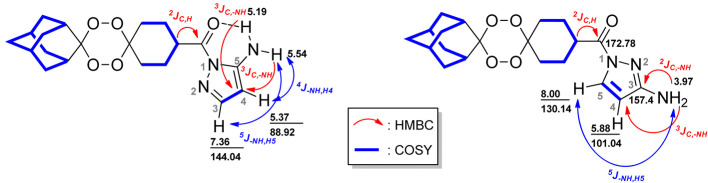							
Isomer T1				Isomer T2			
^1^H, δ (ppm)		^13^C, δ (ppm)		^1^H, δ (ppm)		^13^C, δ (ppm)	
	COSY	HSQC	HMBC		COSY	HSQC	HMBC
1.60	1.62, 1.70, 1.73, 1.86	33.19	-	1.59	1.69	33.19	-
1.62	1.60, 1.70, 1.73,	30.47	-	1.69	1.59, 1.71	37.01	33.34
1.70	1.73	37.01	33.34	1.71	3.14, 1.76	37.01	26.99, 33.34
1.73	1.70, 3.16	33.19	26.99	1.76	1.69, 1.71, 3.14, 1.97	33.19	-
1.86	1.97, 3.16, 3.65	30.56, 33.19	-	1.83	3.48	27.21	-
1.97	1.73, 3.65	27.05	-	1.87	1.76, 2.15, 3.48	28.21	30.17, 34.07
3.16	1.73	30.2	-	1.97	1.76, 2.15	33.19	172.78
3.65	1.86, 1.97	41.33	178.1	2.02	1.87. 1.97	33.19	-
5.19	-	-NH	88.92	3.14	1.71, 1.76	-	-
5.37	7.36, 5.54	88.92	143.97, 150.32	3.48	1.85, 1.87	40.00	172.78
5.54	5.37, 7.36	-NH	88.92	3.97	8.00	-NH_2_	101.04, 157.40
7.36	5.37; 5.54	144.04	89.02, 150.32	5.88	8.00	101.04	130.14, 157.40
-	-	-	-	8.00	3.97, 5.88	130.14	101.04, 157.40

**Table 3:** Hydrogen bonding information for the crystalline structures of isomers **T1** and **T2**. Symmetry codes: a: x,y,−1+z; b: 1−x,2−y,1−z; c: 1−x,1/2+y,1/2−z.

T1: D–H^…^A	D–H (Å)	H^…^A (Å)	D^…^A (Å)	<D–H^…^A (°)
N3–H3A^…^N2 ^a^	0.88	2.55	3.528 (11)	137
N3–H3B^…^O5	0.88	2.15	2.755 (11)	125
**T2: D–** **H^…^A**	**D–** **H (Å** **)**	**H^…^A (Å** **)**	**D^…^A (Å** **)**	**<D–** **H^…^A (°)**
N3–H3A^…^N2 ^b^	0.868 (18)	2.192 (18)	3.0432 (16)	166.7 (17)
N3–H3B^…^O5 ^c^	0.830 (18)	2.513 (18)	3.1726 (17)	137.2 (16)

**Table 4:** Estimated ClogP and ClogS values, cytotoxic concentrations (CC_50_) in THP-1 cell line, inhibitory concentrations (IC_50_) in promastigotes of *L. infantum* and *L. tropica* and intracellular amastigotes of *L. infantum*, and selectivity index (SI) of synthetic endoperoxide–pyrazole hybrids, and amphotericin B (control).

ID	Structures	ClogP ^a^	ClogS ^a^	Cytotoxicity CC_50_ ± SEM ^b^ (µΜ)	Promastigotes Susceptibility IC_50_ ± SEM ^b^ (µΜ)		Intracellular Amastigote Susceptibility IC_50_ ± SEM ^c^ (µΜ)	SI ^d^
					*L. tropica*	*L. infantum*	*L. infantum*	
**2o**	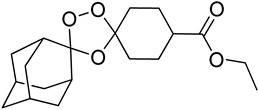	2.61	1.38	381 ± 172	156 ± 18	123 ± 1	330 ± 48	1.2
**2t**	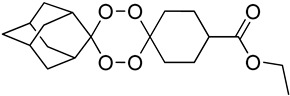	2.38	1.21	374 ± 119	143 ± 9	128 ± 27	45 ± 28	8.4
**OZ1**	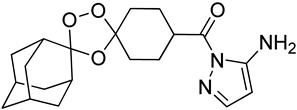	2.01	1.30	664 ± 163	219 ± 25	>400	ND	ND
**OZ2**	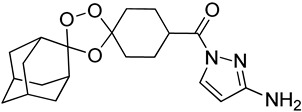	2.01	1.30	202 ± 116	161 ± 19	219 ± 44	ND	ND
**T1**	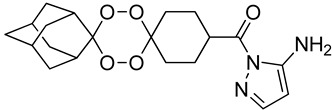	1.79	1.18	>800	>400	>400	ND	ND
**T2**	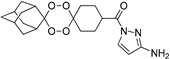	1.79	1.18	>800	ND	ND	ND	ND
**PIR**	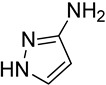	−0.51	5.59	202 ± 10	>400	>400	ND	ND
**AmB**	Amphotericin B	−1.19	2.48	23 ± 3	0.30 ± 0.1	0.20 ± 0.01	0.08 ± 0.05	290

^a^ Calculated using StarDrop v7.2.0.32905; ^b^ SEM: standard error of mean of at least three independent assays; ^c^ SEM: standard error of mean of two independent assays; ^d^ SI for amastigote forms; ND: not defined.

**Table 5:** Cytotoxic concentrations (CC_50_) in THP-1 cell line, inhibitory concentrations (IC_50_) in promastigote forms of *L. infantum* and *L. tropica*, and in intracellular amastigote forms of *L. infantum* and selective index (SI) of synthetic endoperoxide–pyrazole salts.

Compounds	Promastigotes Susceptibility IC_50_ ± SEM ^a^ (µΜ)		Cytotoxicity CC_50_ ± SEM ^a^ (µΜ)	Intracellular Amastigotes Susceptibility IC_50_ ± SEM ^b^ (µΜ)	SI ^c^
	*L. tropica*	*L. infantum*		*L. infantum*	
**OZ1•TsOH**	199 ± 9	181 ± 35	510 ± 109	246 ± 32	2.1
**OZ1•MsOH**	198 ± 24	183 ± 48	336 ± 196	>400	<0.8
**OZ1•HCl**	135 ± 36	164 ± 20	509 ± 155	87 ± 39	5.9
**OZ2•TsOH**	260 ± 82	217 ± 47	479 ± 83	ND	ND
**OZ2•HCl**	>400	258 ± 91	425 ± 104	ND	ND
**T1•TsOH**	>400	>400	331 ± 126	ND	ND
**T1•HCl**	>400	>400	529 ± 108	ND	ND
**T2•HCl**	266 ± 8	313 ± 40	444 ± 22	ND	ND

^a^ SEM: Standard error of mean of at least three independent assays; ^b^ SEM: standard error of mean of two independent assays; ^c^ SI for amastigote forms; ND: not defined.

## Data Availability

CIF files containing supplementary crystallographic data were deposited at the Cambridge Crystallographic Data Centre with references 2151742 (**T1**) and 2151175 (**T2**).

## Supplementary Materials

The following supporting information can be downloaded at: https://www.mdpi.com/article/10.3390/molecules27175401/s1, S1. ^1^H, ^13^C{^1^H} NMR, COSY, HSQC, HMBC spectra of the synthesized compounds. S2. HRMS spectra of the synthesized compounds. S3. X-ray crystallography. Figure S1: ^1^H NMR spectrum (400 MHz) of 1o in CDCl_3_. Figure S2: ^13^C{^1^H} NMR spectrum (100 MHz) of 1o in CDCl_3_. Figure S3. ^1^H NMR spectrum (500 MHz) of 2o in CDCl_3_. Figure S4. ^13^C{^1^H} NMR spectrum (126 MHz) of 2o in CDCl_3_. Figure S5. ^1^H NMR spectrum (500 MHz) of 2t in CDCl_3_. Figure S6. ^13^C{^1^H} NMR spectrum (126 MHz) of 2t in CDCl_3_. Figure S7. ^1^H NMR spectrum (500 MHz) of 3o in CDCl_3_. Figure S8. ^13^C{^1^H} NMR spectrum (126 MHz) of 3o in CDCl_3_. Figure S9. ^1^H NMR spectrum (500 MHz) of 3t in CDCl_3_. Figure S10. ^13^C{^1^H} NMR spectrum (126 MHz) of 3t in CDCl_3_. Figure S11. ^1^H NMR spectrum (500 MHz) of OZ1 in CDCl_3_. Figure S12. ^13^C{^1^H} NMR spectrum (126 MHz) of OZ1 in CDCl_3_. Figure S13. ^13^C{^1^H} DEPT-135 NMR spectrum (126 MHz) of OZ1 in CDCl_3_. Figure S14. COSY spectrum (500 MHz) of OZ1 in CDCl_3_. Figure S15. HSQC spectrum (500 MHz) of OZ1 in CDCl_3_. Figure S16. HMBC spectrum (500 MHz) of OZ1 in CDCl_3_. Figure S17. ^1^H NMR spectrum (500 MHz) of OZ2 in CDCl_3_. Figure S18. 1^13^C{^1^H} NMR spectrum (126 MHz) of OZ2 in CDCl_3_. Figure S19. COSY spectrum (500 MHz) of OZ2 in CDCl_3_. Figure S20. HSQC spectrum (500 MHz) of OZ2 in CDCl_3_. Figure S21. HMBC spectrum (500 MHz) of OZ2 in CDCl_3_. Figure S22. ^1^H NMR spectrum (500 MHz) of T1 in CDCl_3_. Figure S23. ^13^C{^1^H} NMR spectrum (126 MHz) of T1 in CDCl_3_. Figure S24. ^13^C{^1^H} DEPT-135 NMR spectrum (126 MHz) of T1 in CDCl_3_. Figure S25. COSY spectrum (500 MHz) of T1 in CDCl_3_. Figure S26. HSQC spectrum (500 MHz) of T1 in CDCl_3_. Figure S27. HMBC spectrum (500 MHz) of T1 in CDCl_3_. Figure S28. ^1^H NMR spectrum (500 MHz) of T2 in CDCl_3_. Figure S29. ^13^C{^1^H} NMR spectrum (126 MHz) of T2 in CDCl_3_. Figure S30. ^13^C{^1^H} DEPT-135 NMR spectrum (126 MHz) of T2 in CDCl_3_. Figure S31. COSY spectrum (500 MHz) of T2 in CDCl_3_. Figure S32. HSQC spectrum (500 MHz) of T2 in CDCl_3_. Figure S33. HMBC spectrum (500 MHz) of T2 in CDCl_3_. Figure S34. ^1^H NMR spectrum (500 MHz) of OZ1•TsOH in DMSO-*d*_6_. Figure S35. ^13^C{^1^H} NMR spectrum (126 MHz) of OZ1•TsOH in DMSO-*d*_6_. Figure S36. ^1^H NMR spectrum (500 MHz) of OZ1•MsOH in DMSO-*d*_6_. Figure S37. ^13^C{^1^H} NMR spectrum (126 MHz) of OZ1•MsOH in DMSO-*d*_6_. Figure S38. ^1^H NMR spectrum (500 MHz) of OZ2•TsOH in DMSO-*d*_6_. Figure S39. ^13^C{^1^H} NMR spectrum (126 MHz) of OZ2•TsOH in DMSO-*d*_6_. Figure S40. ^1^H NMR spectrum (500 MHz) of T1•TsOH in DMSO-*d*_6_. Figure S41. ^13^C{^1^H} NMR spectrum (126 MHz) of T1•TsOH in DMSO-*d*_6_. Figure S42. ^1^H NMR spectrum (500 MHz) of OZ1•HCl in DMSO-*d*_6_. Figure S43. ^13^C{^1^H} NMR spectrum (126 MHz) of OZ1•HCl in DMSO-*d_6_.* Figure S44. ^1^H NMR spectrum (500 MHz) of OZ2•HCl in DMSO-*d*_6_. Figure S45. ^13^C{^1^H} NMR spectrum (126 MHz) of OZ2•HCl in DMSO-*d*_6_. Figure S46. ^1^H NMR spectrum (500 MHz) of T1•HCl in DMSO-*d*_6_. Figure S47. ^13^C{^1^H} NMR spectrum (126 MHz) of T1•HCl in DMSO-*d*_6_. Figure S48. ^1^H NMR spectrum (500 MHz) of T2•HCl in DMSO-*d*_6_. Figure S49. ^13^C{^1^H} NMR spectrum (126 MHz) of T2•HCl in DMSO-*d_6_.* Figure S50. ORTEP drawing of the molecule of (5-amino-*1H*-pyrazol-1-yl) (dispiro[cyclohexane-1,3′-[1,2,4,5]tetraoxane-6′,2″-tricyclo[3.3.1.1^3,7^]decan]-4-yl)-methanone (T1). Figure S51. ORTEP drawing of the molecule of (3-amino-*1H*-pyrazol-1-yl) (dispiro[cyclohexane-1,3′-[1,2,4,5]tetraoxane-6′,2″-tricyclo[3.3.1.1^3,7^]-decan]-4-yl)-methanone (T2).

## Sample Availability

The samples are available from the authors.
