# Supplementary material for: Synthesis, Structure and Antileishmanial Evaluation of Endoperoxide–Pyrazole Hybrids
Source: Molecules. 2022 Aug 24;27(17):5401. doi: 10.3390/molecules27175401 (PMC9457810; doi:10.3390/molecules27175401)
Supplement: Supplementary file 1 [file molecules-27-05401-s001.zip › molecules-1835290-supplementary.pdf]

Supporting Information

Synthesis, Structure and Antileishmanial Evaluation of Endoperoxide–Pyrazole Hybrids

Patrícia S. M. Amado [1,2,†], Inês C. C. Costa [1,2], José A. Paixão [3] , Ricardo F. Mendes [4], Sofia Cortes [5] and Maria L. S. Cristiano [1,2,*]

1 Center of Marine Sciences, CCMAR, Gambelas Campus, University of Algarve, UAlg, 8005-039 Faro Portugal

2 Department of Chemistry and Pharmacy, Faculty of Sciences and Technology, FCT, Gambelas Campus,University of Algarve, UAlg, 8005-139 Faro, Portugal

3 CFisUC, Department of Physics, University of Coimbra, 3004-516 Coimbra, Portugal

4 CICECO-Aveiro Institute of Materials, University of Aveiro, 3810-193 Aveiro, Portugal

5 Global Health and Tropical Medicine-GHTM, Instituto de Higiene e Medicina Tropical-IHMT,Universidade Nova de Lisboa-NOVA, 1349-008 Lisboa, Portugal

* Correspondence: mcristi@ualg.pt; Tel.: +351-289-800-953

† These authors contributed equally to this work.

Table of contents

S1. ^{1}H, $^{13}C\{^{1}H\}$ NMR, COSY, HSQC, HMBC spectra of the synthesised compounds S2

S2. HRMS spectra of the synthesised compounds S51

S3. X-ray crystallography S60

S1. ^{1}H, ^{13}C{^{1}H} NMR, COSY, HSQC, HMBC spectra of the synthesised compounds

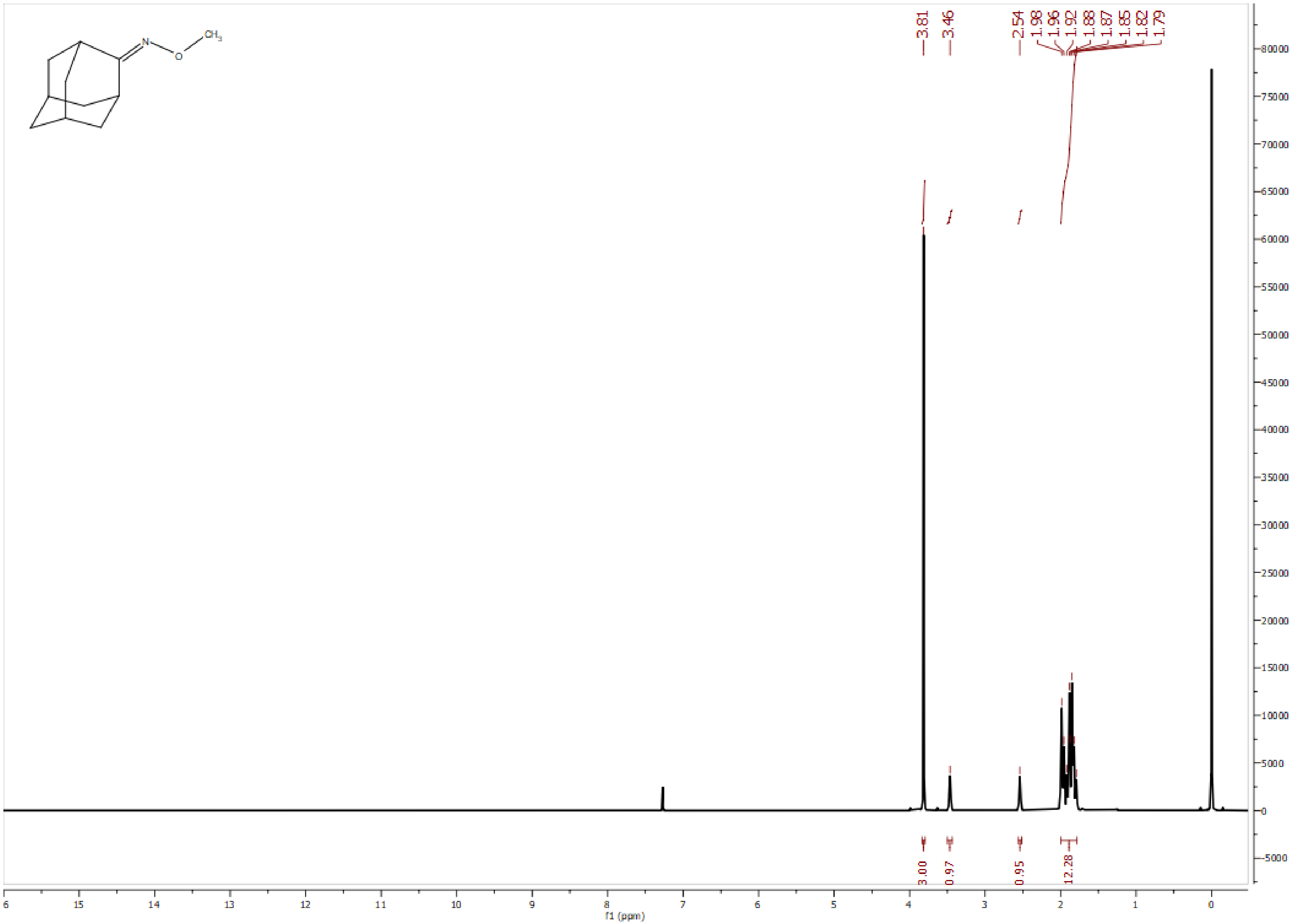

Figure S1. ^{1}H NMR spectrum (400 MHz) of **1o** in $CDCl_3$.

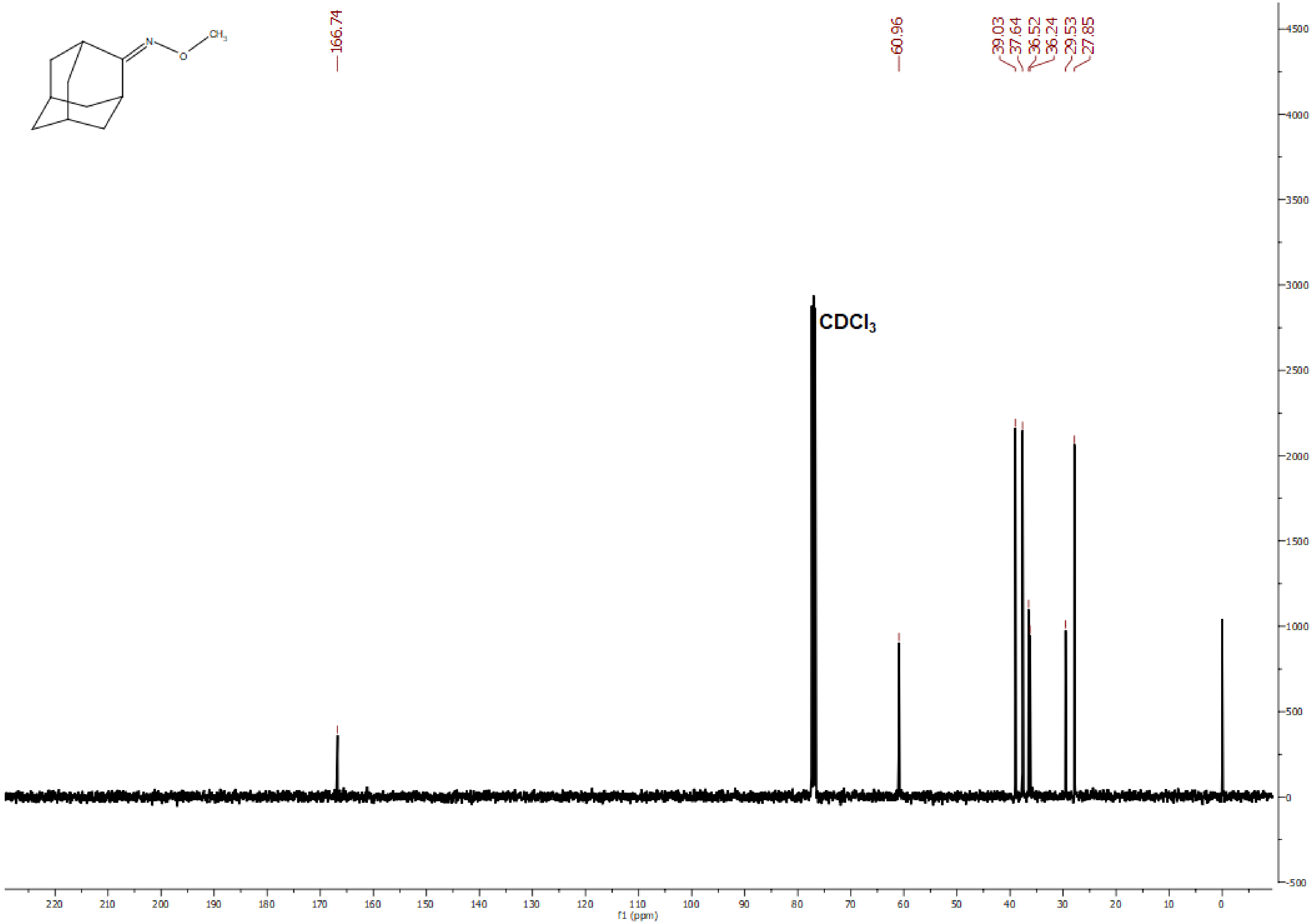

Figure S2. $^{13}C\{^{1}H\}$ NMR spectrum (100 MHz) of **1o** in $CDCl_3$.

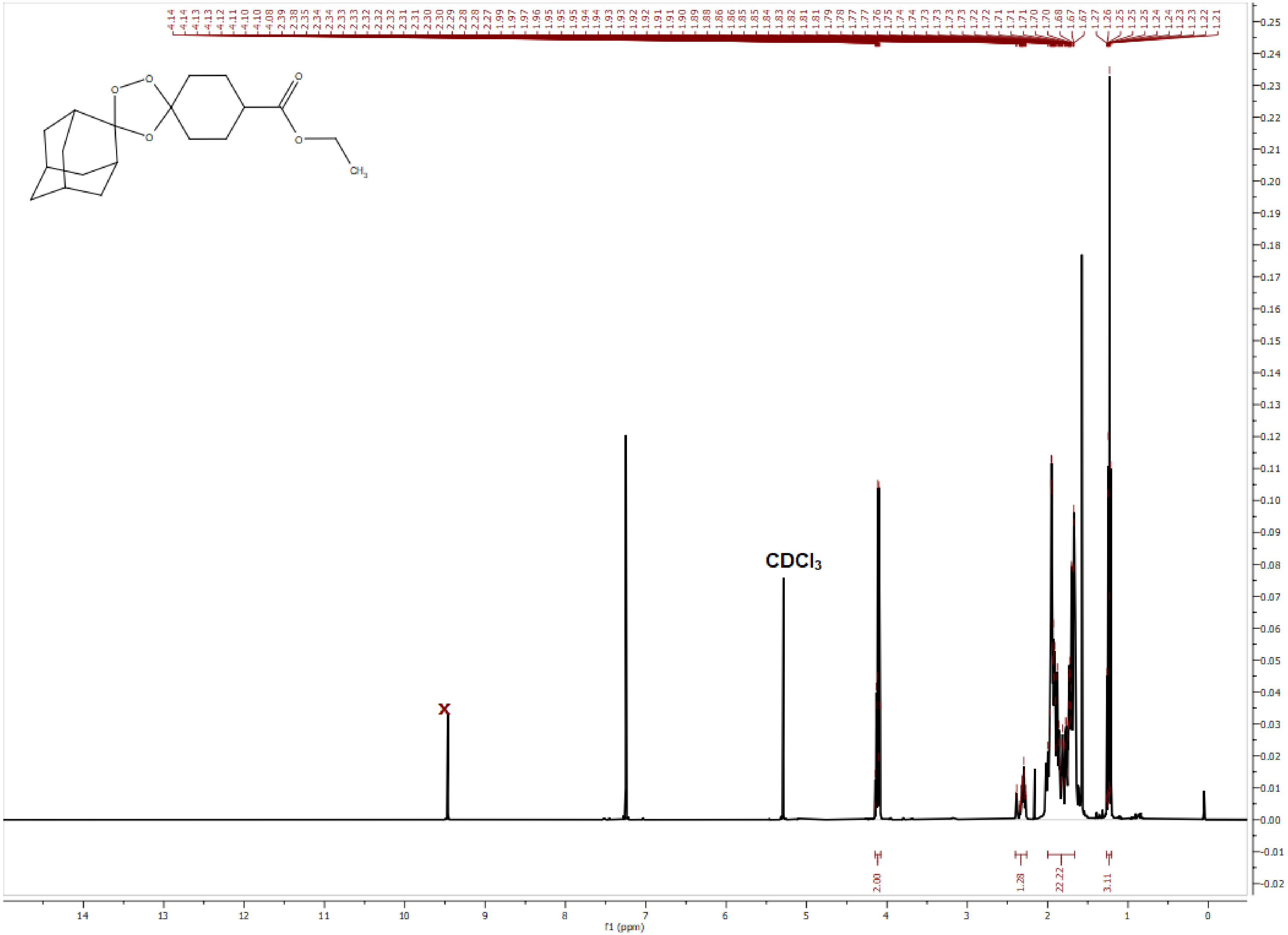

Figure S3. ^{1}H NMR spectrum (500 MHz) of **2o** in $CDCl_3$.

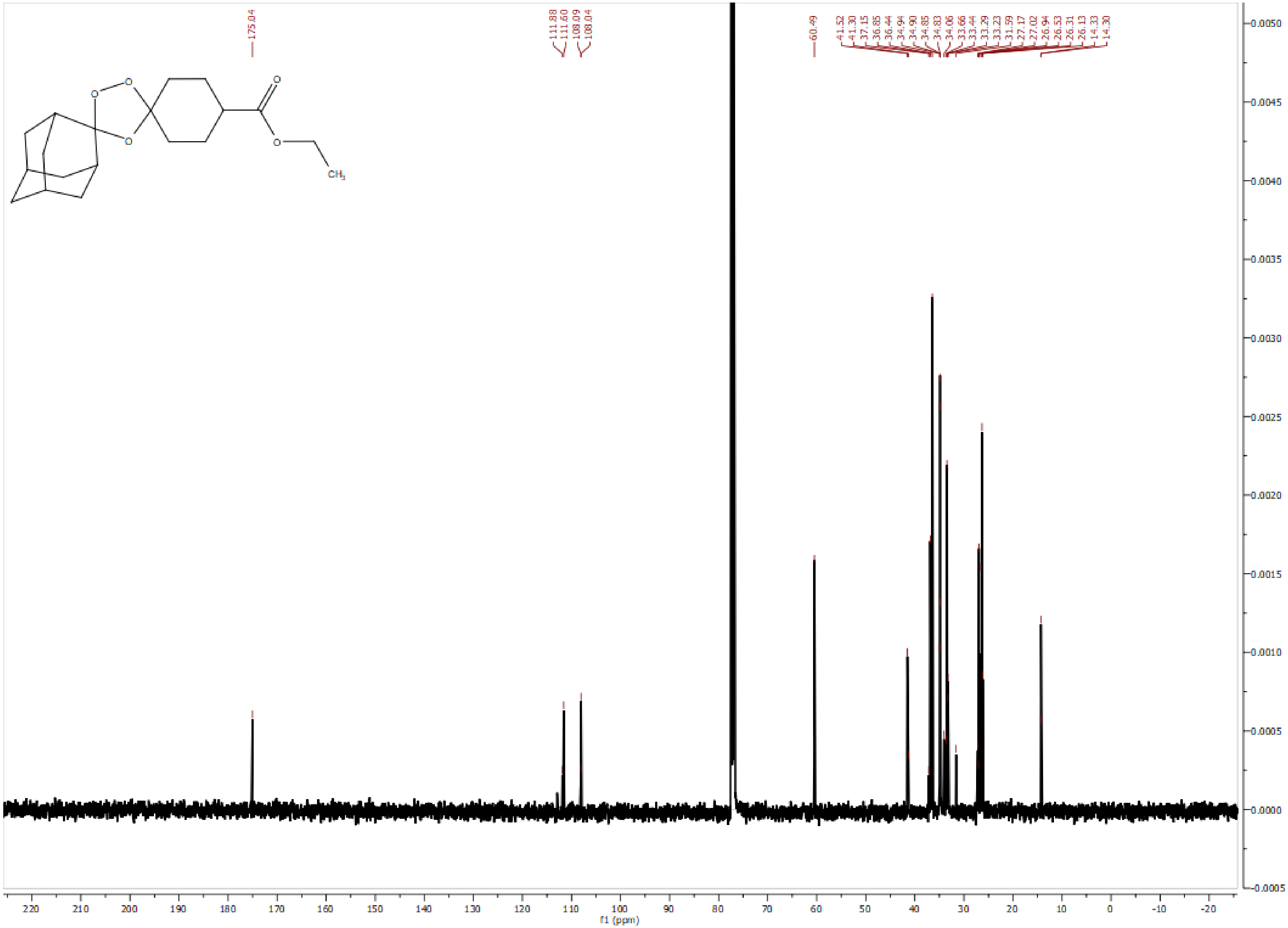

Figure S4. $^{13}C\{^1H\}$ NMR spectrum (126 MHz) of **2o** in $CDCl_3$.

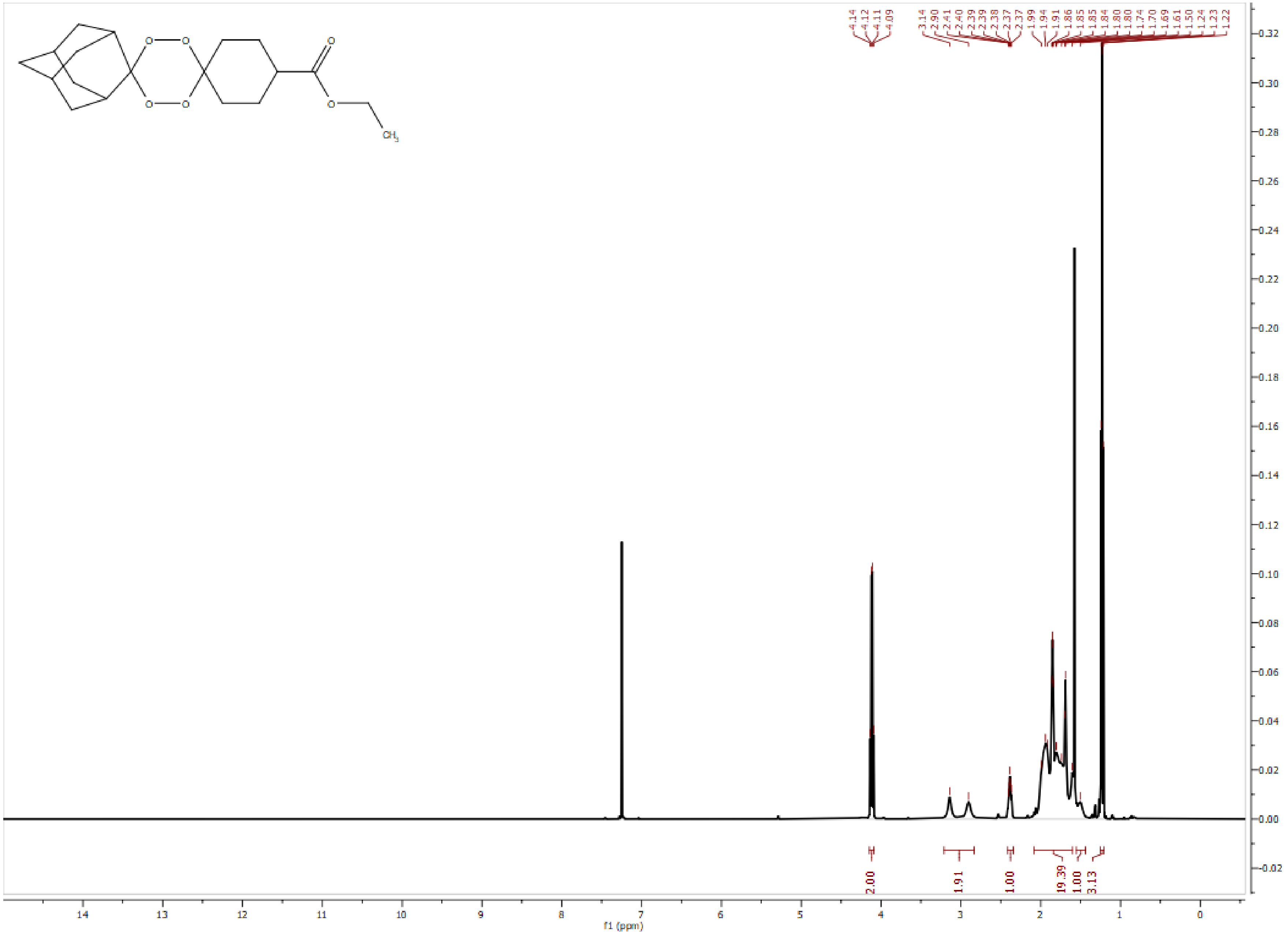

Figure S5. ^{1}H NMR spectrum (500 MHz) of **2t** in $CDCl_3$.

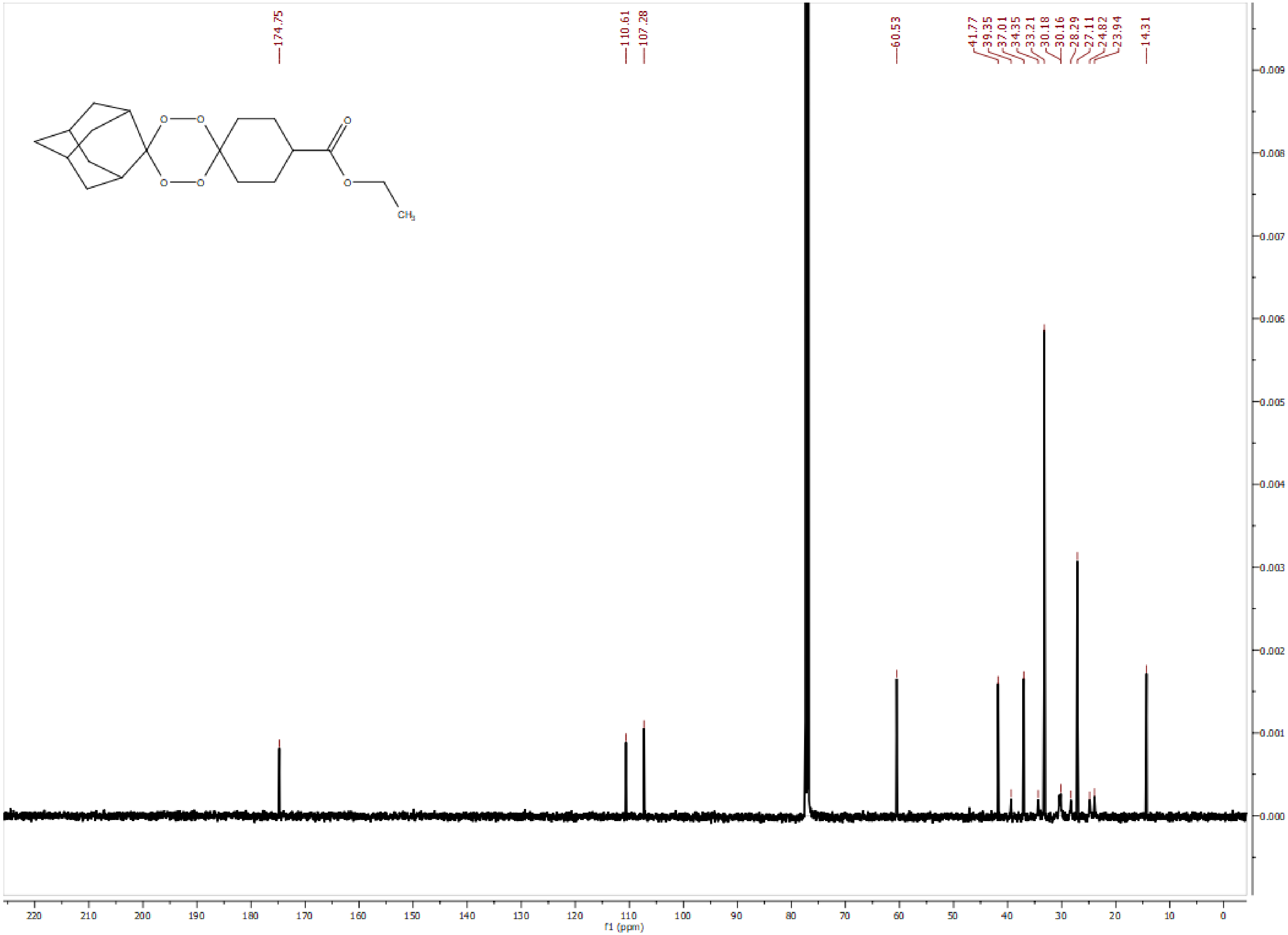

Figure S6. $^{13}C\{^{1}H\}$ NMR spectrum (126 MHz) of **2t** in $CDCl_3$.

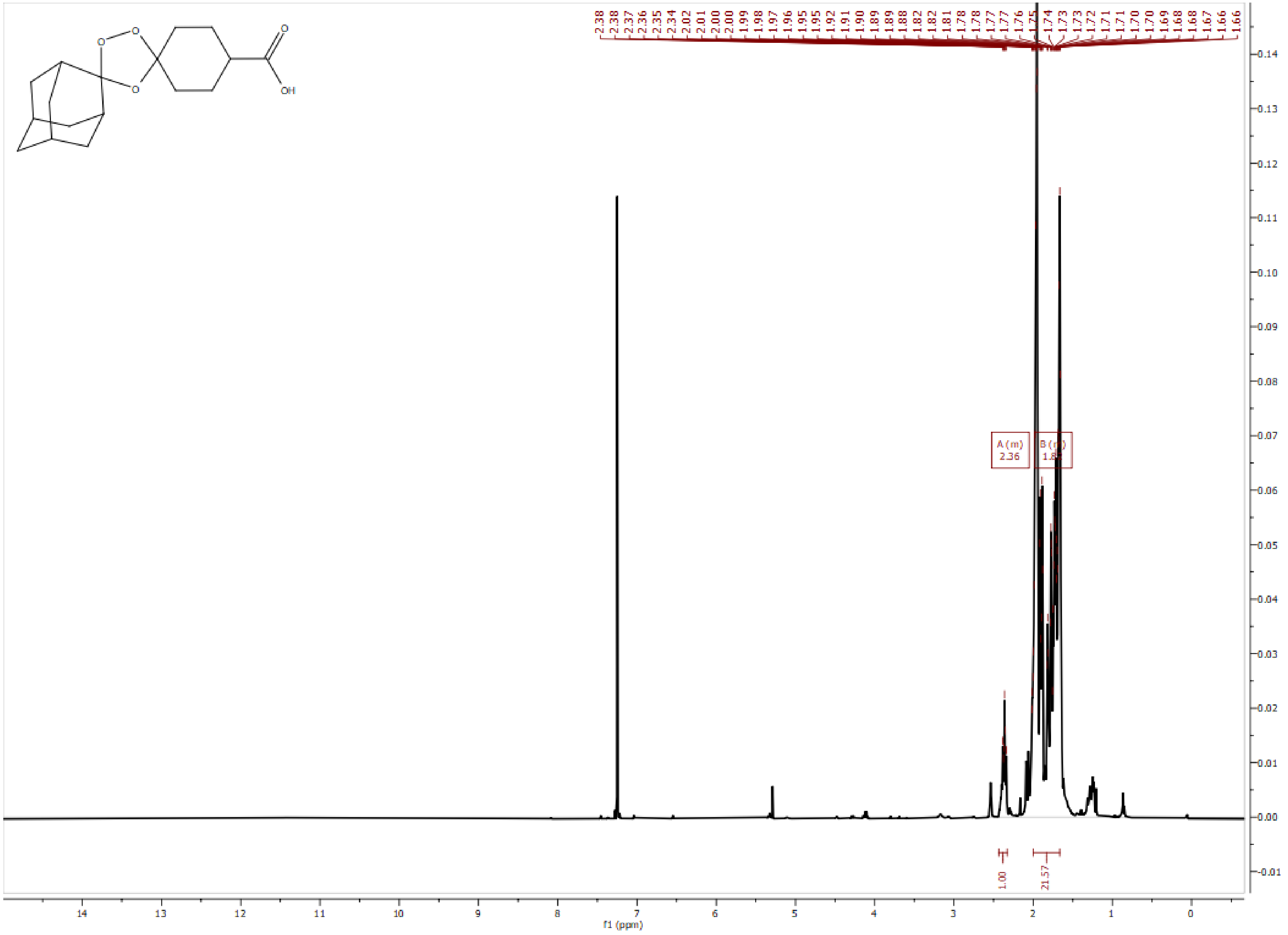

Figure S7. ^{1}H NMR spectrum (500 MHz) of **3o** in $CDCl_3$.

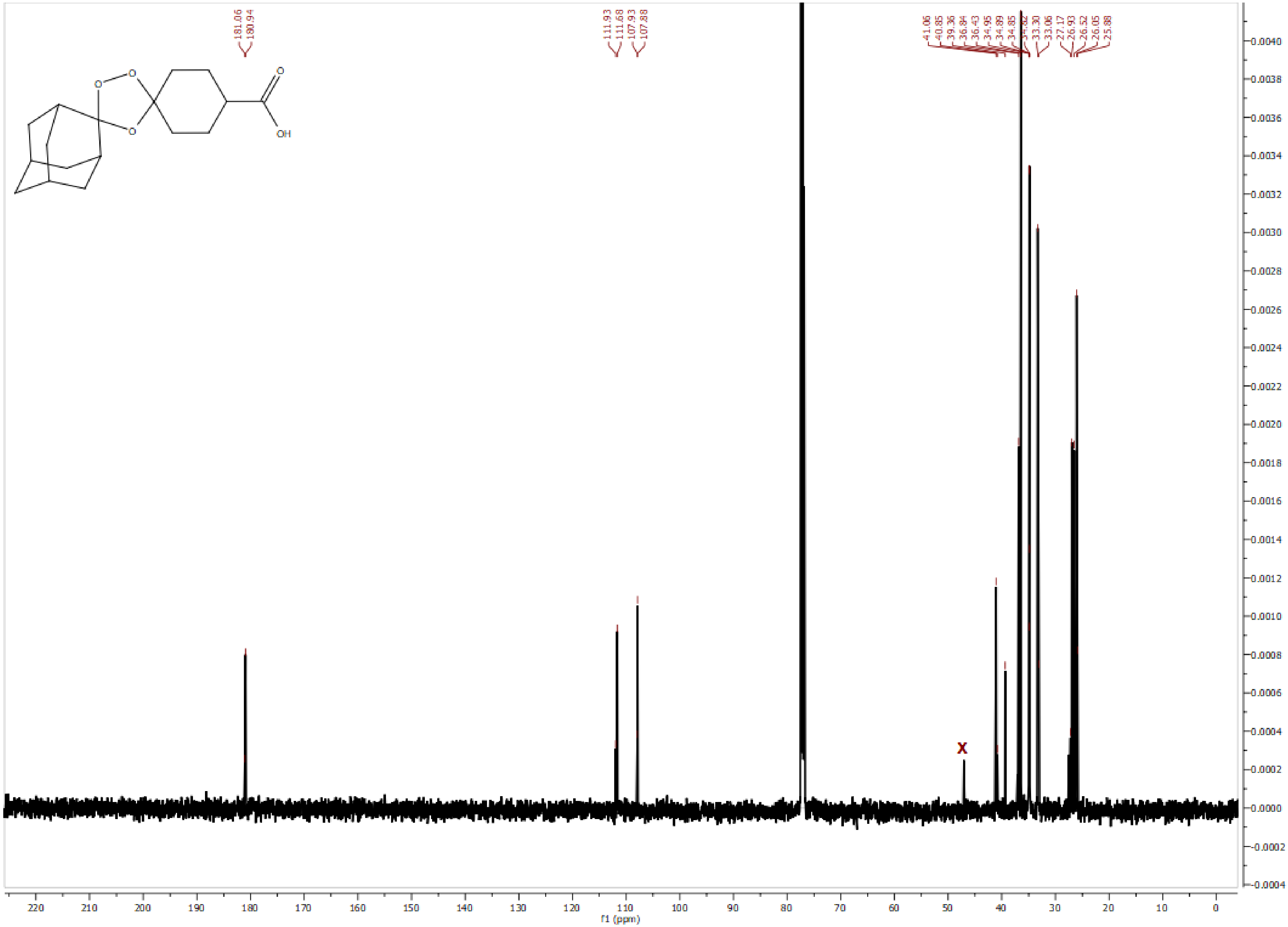

Figure S8. $^{13}C\{^{1}H\}$ NMR spectrum (126 MHz) of **3o** in $CDCl_3$.

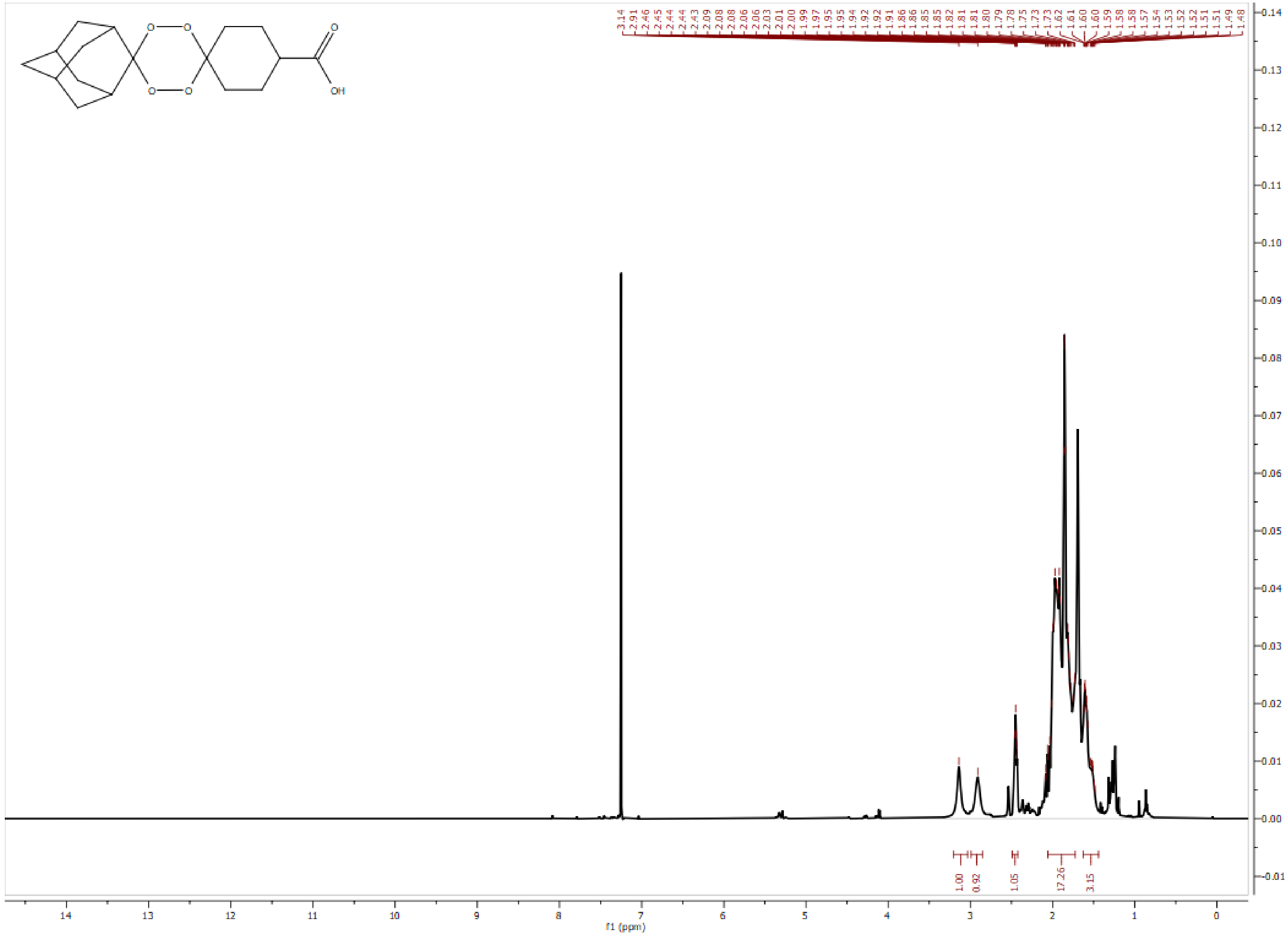

Figure S9. ^{1}H NMR spectrum (500 MHz) of **3t** in $CDCl_3$.

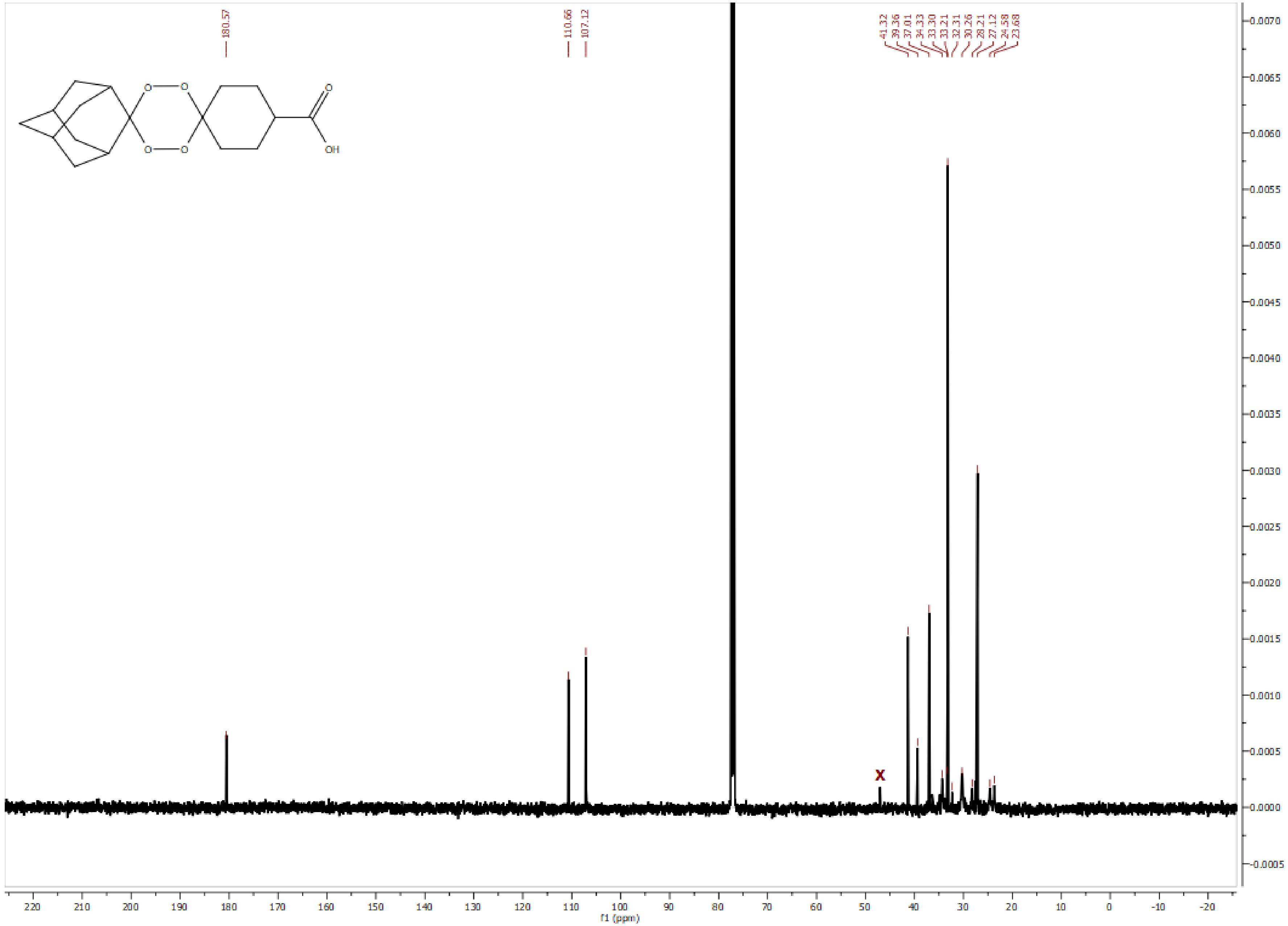

Figure S10. $^{13}C\{^1H\}$ NMR spectrum (126 MHz) of **3t** in $CDCl_3$.

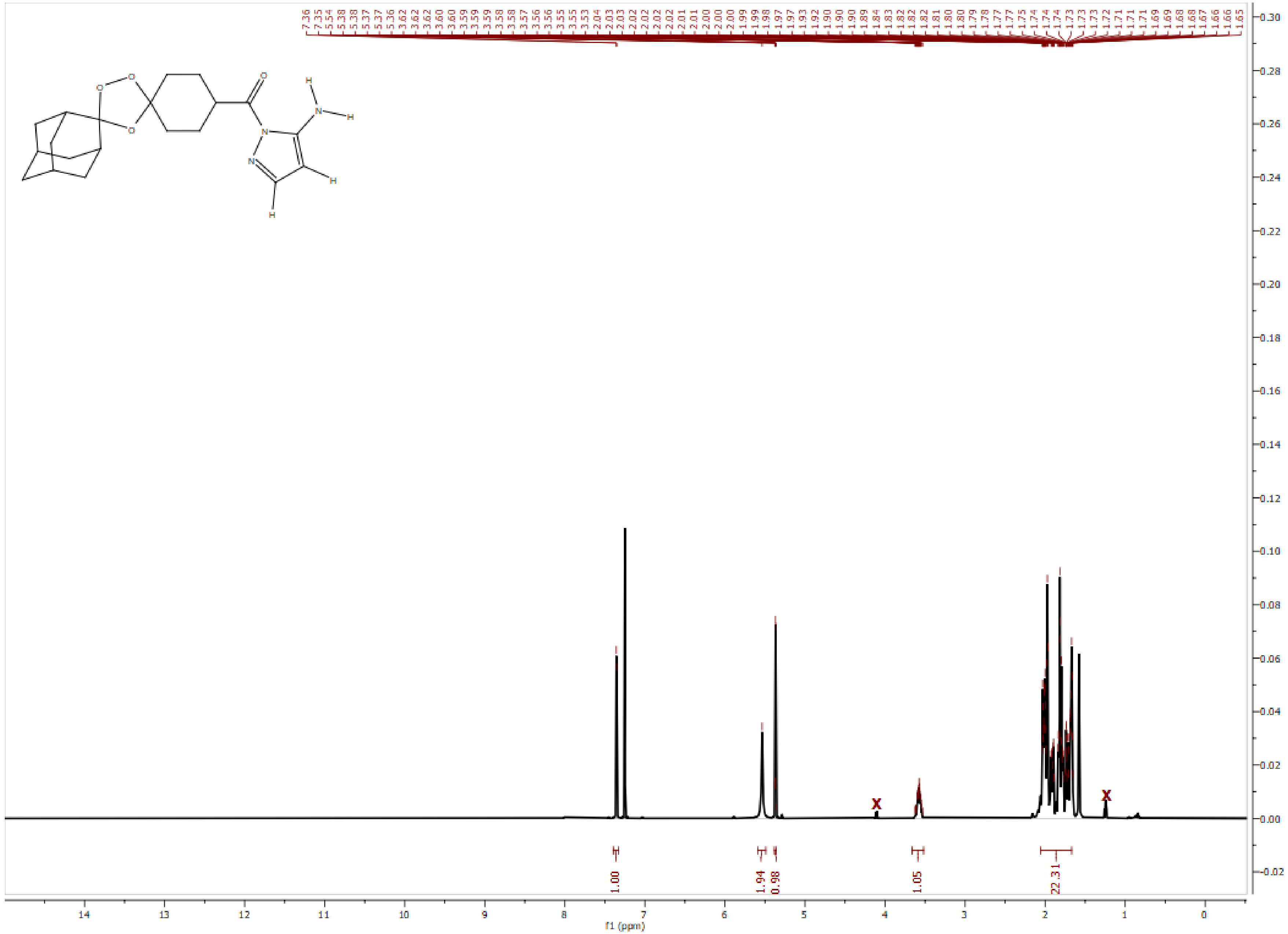

Figure S11. ^{1}H NMR spectrum (500 MHz) of **OZ1** in $CDCl_3$.

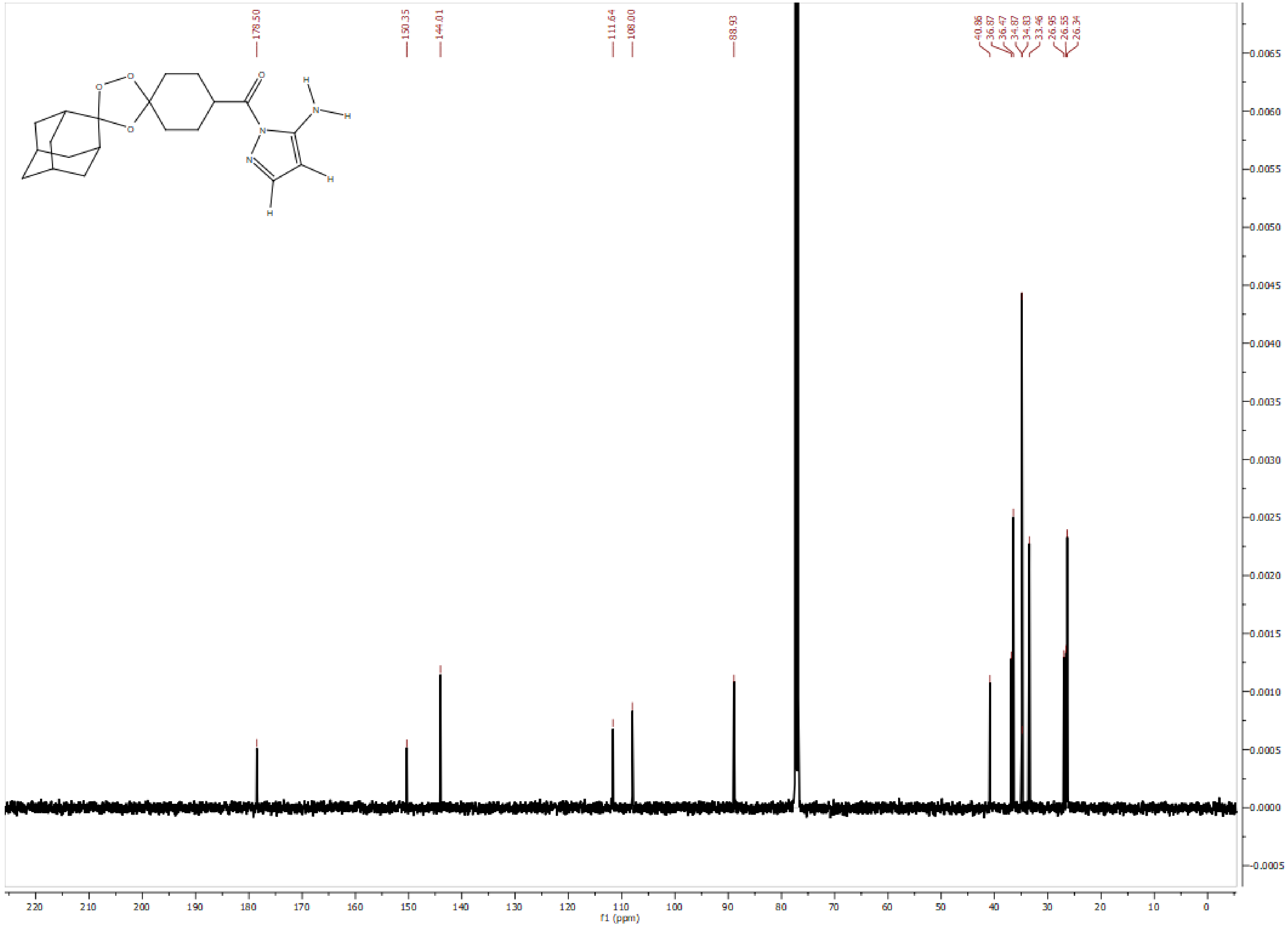

Figure S12. $^{13}C\{^{1}H\}$ NMR spectrum (126 MHz) of **OZ1** in $CDCl_3$.

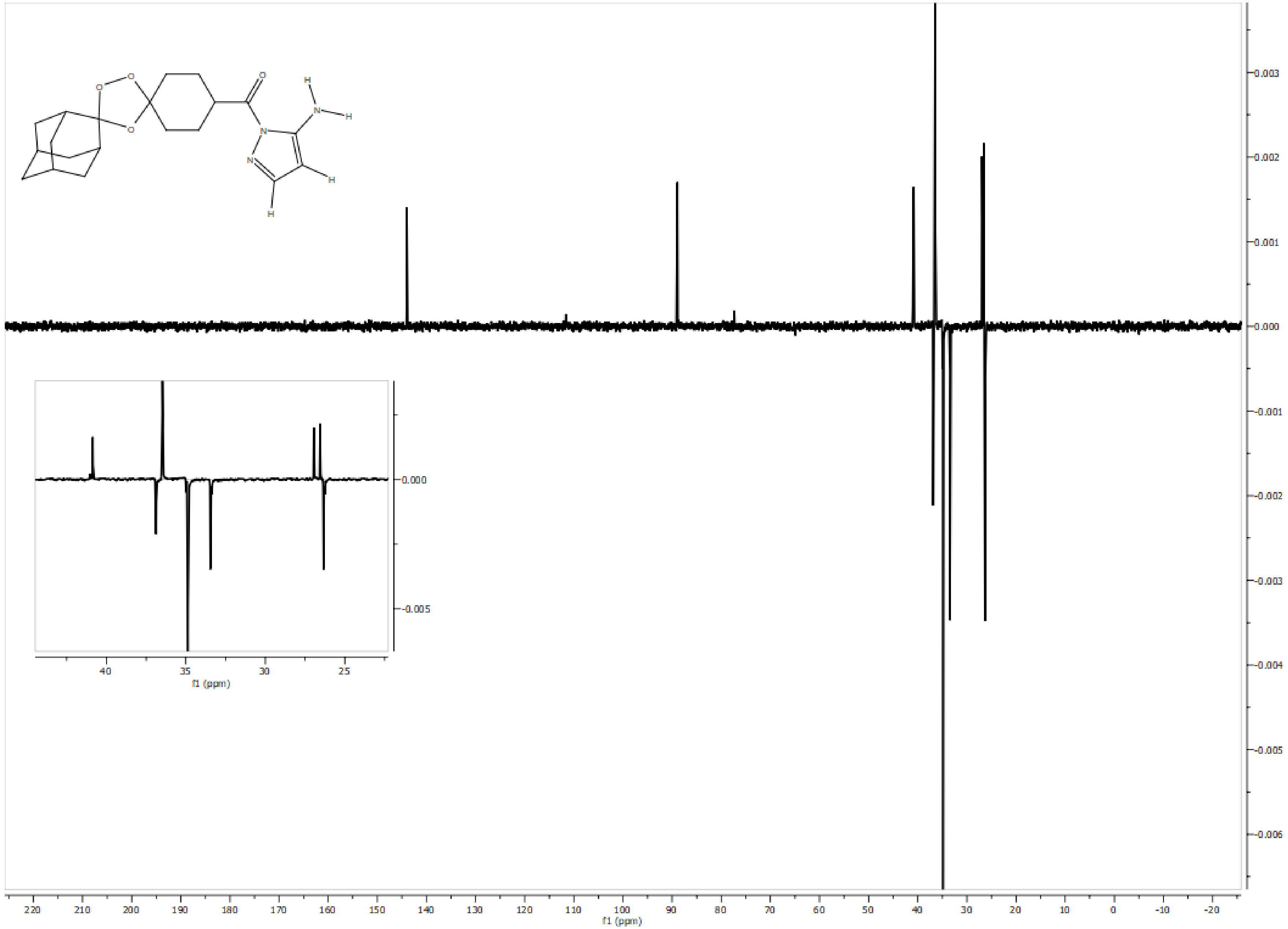

Figure S13. $^{13}C\{^{1}H\}$ DEPT-135 NMR spectrum (126 MHz) of **OZ1** in $CDCl_3$.

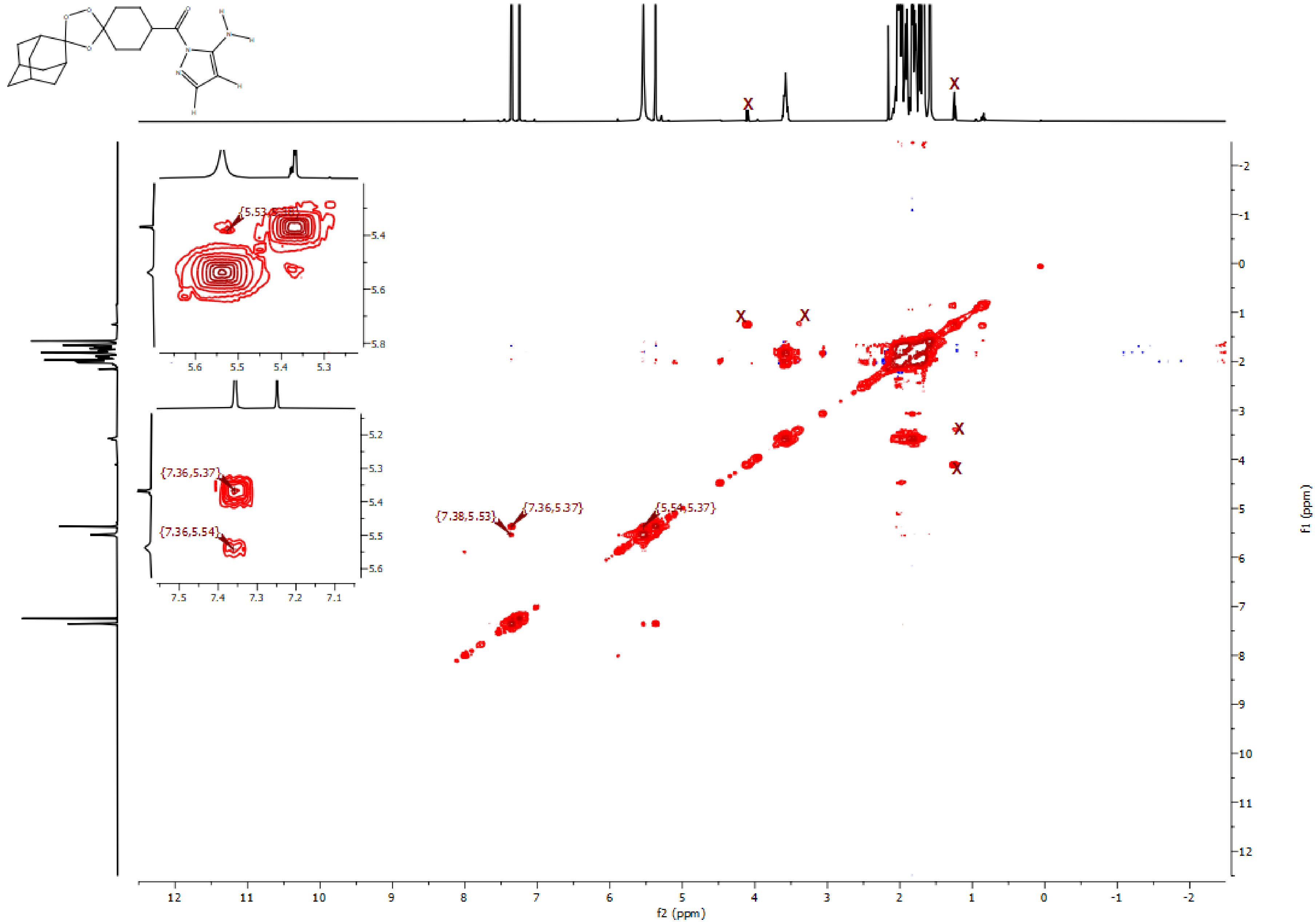

Figure S14. COSY spectrum (500 MHz) of **OZ1** in $CDCl_3$.

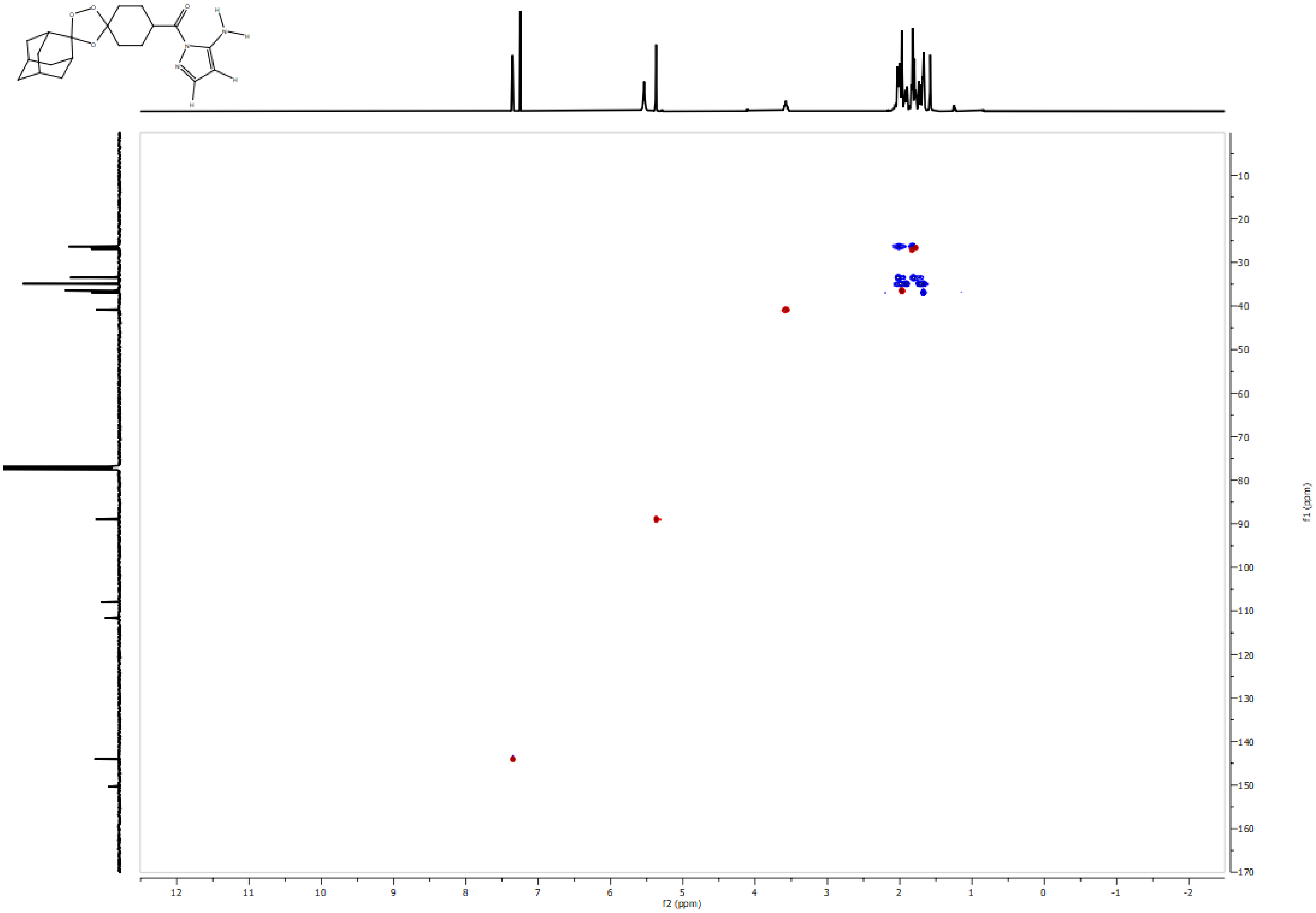

Figure S15. HSQC spectrum (500 MHz) of **OZ1** in $CDCl_3$.

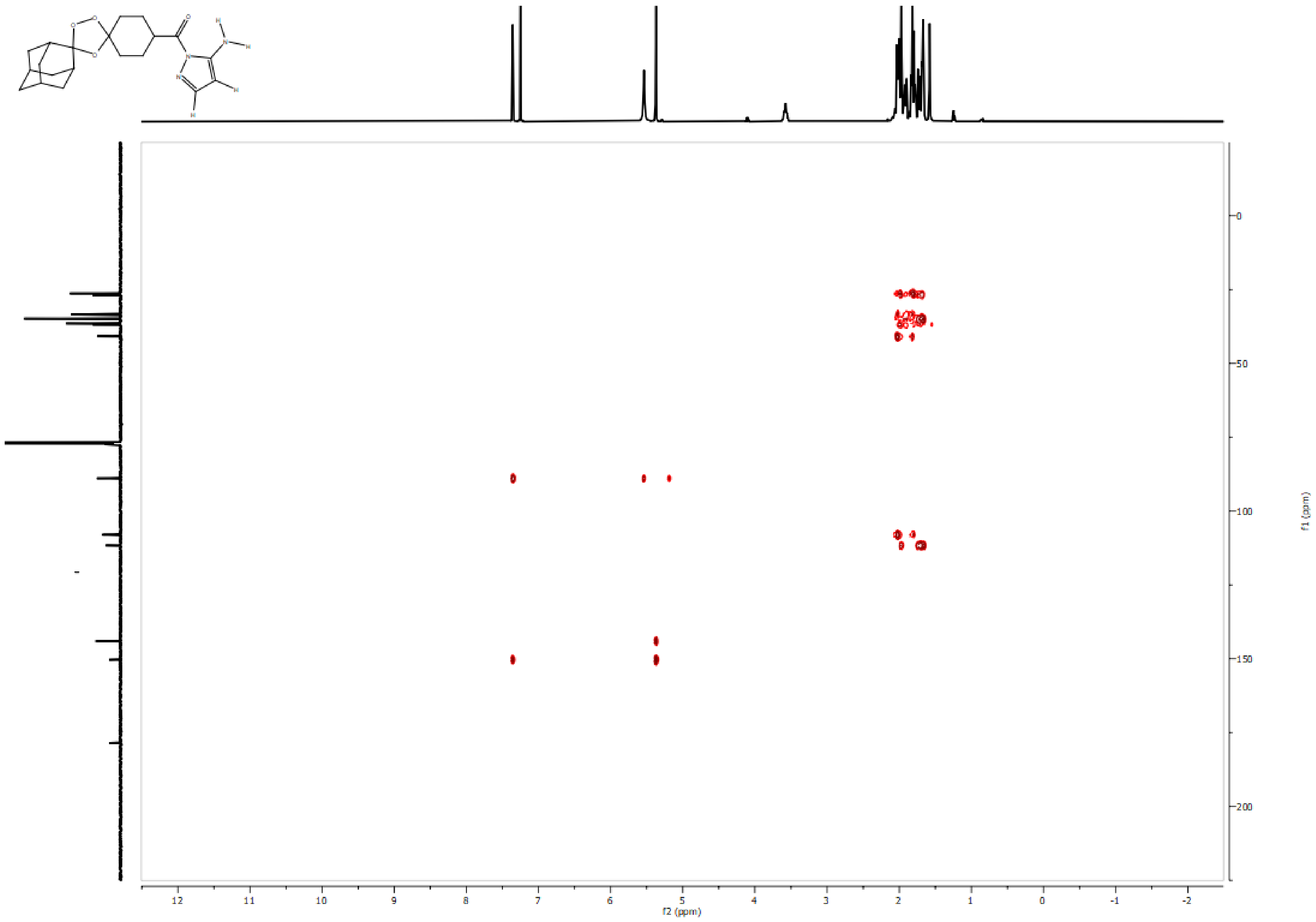

Figure S16. HMBC spectrum (500 MHz) of **OZ1** in $CDCl_3$.

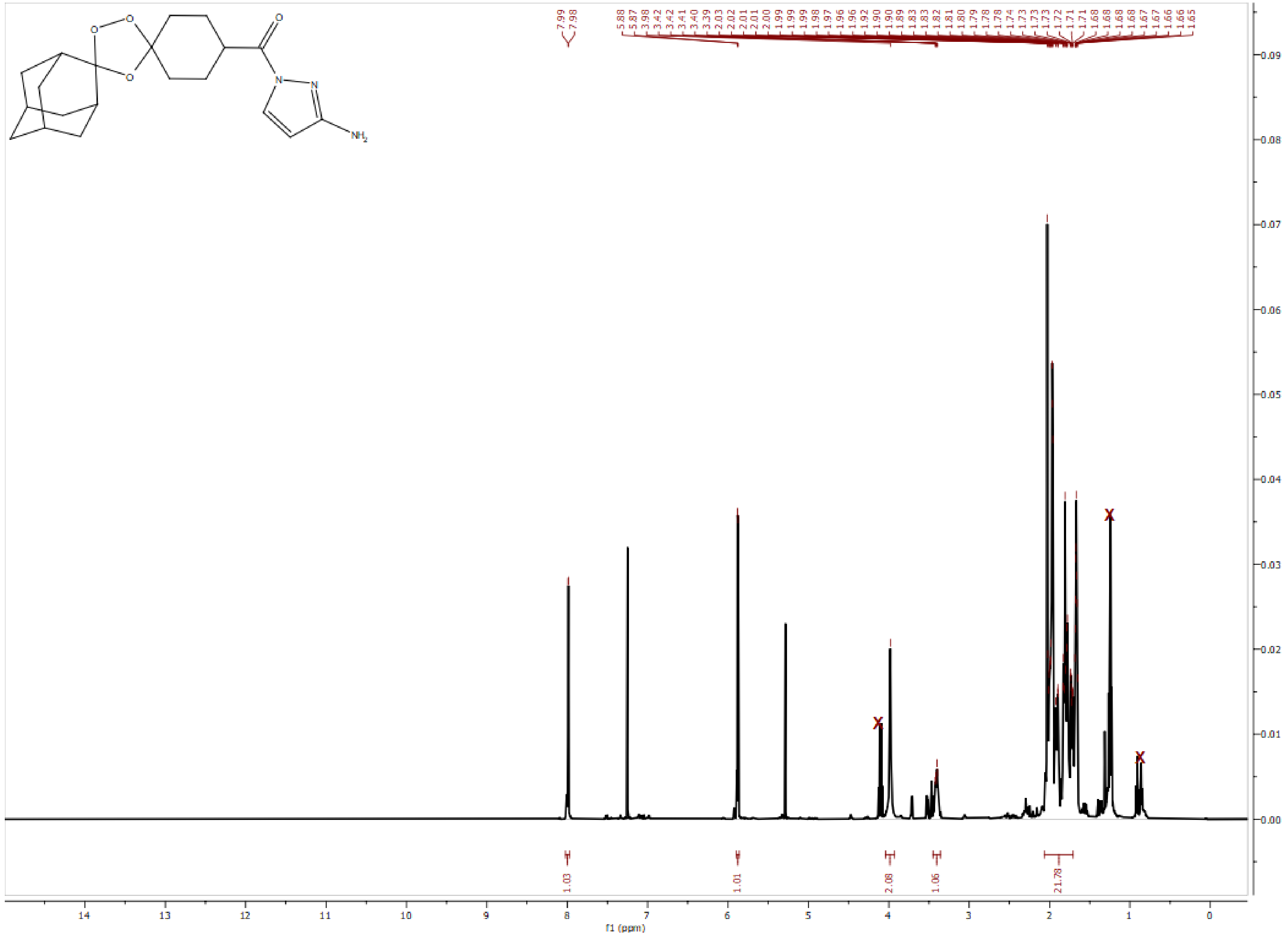

Figure S17. ^{1}H NMR spectrum (500 MHz) of **OZ2** in $CDCl_3$.

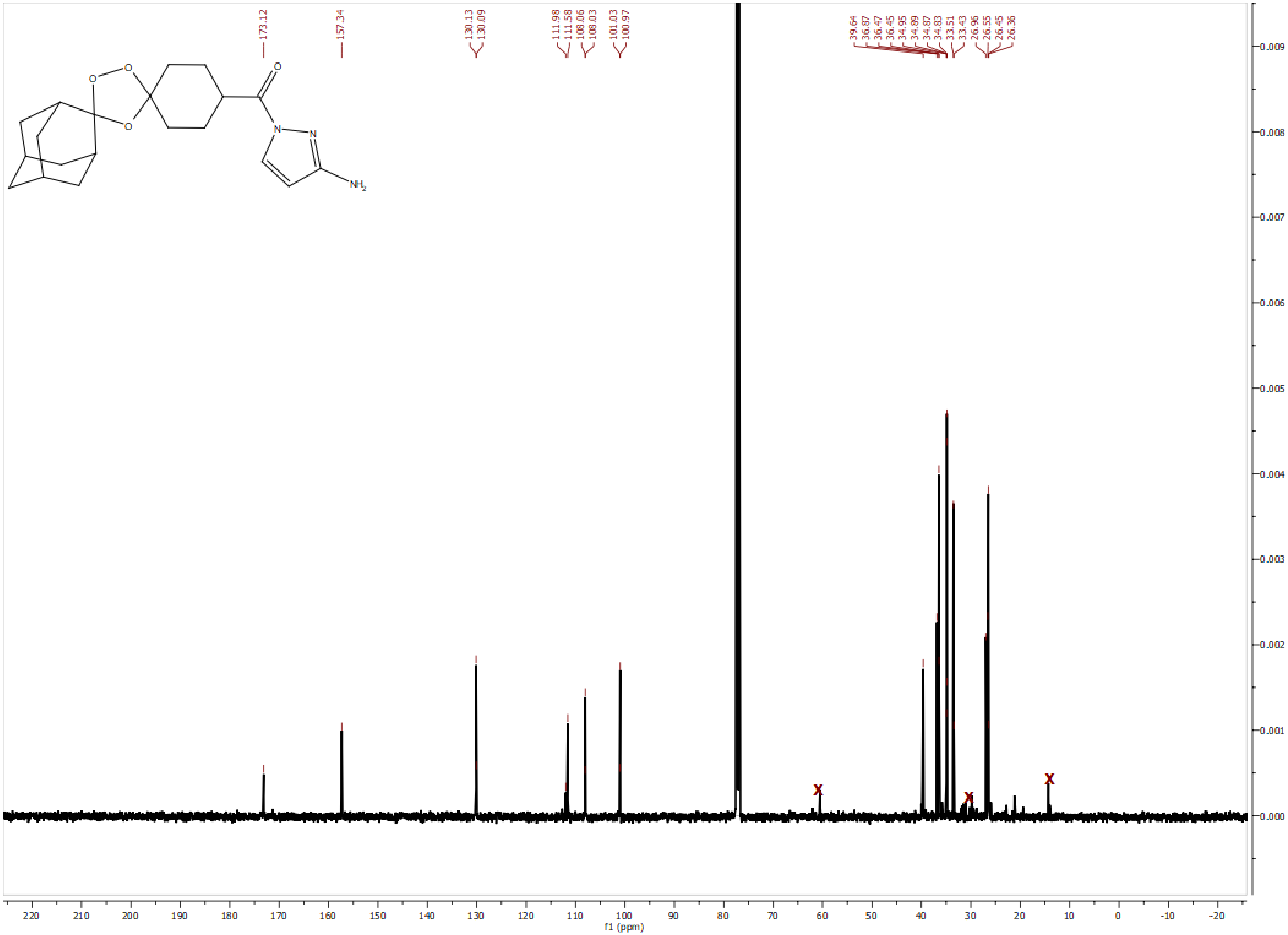

Figure S18. $^{13}C\{^{1}H\}$ NMR spectrum (126 MHz) of **OZ2** in $CDCl_3$.

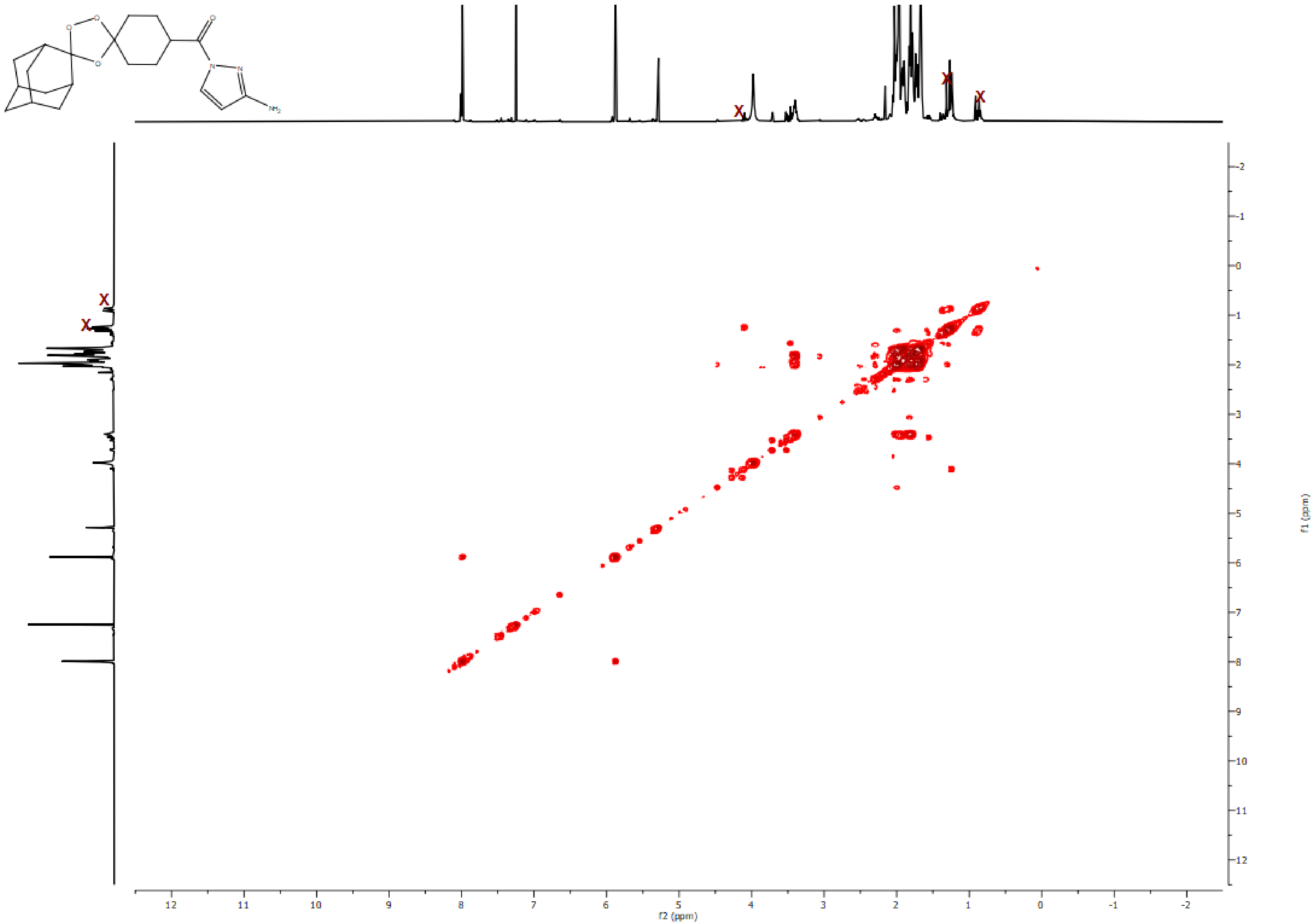

Figure S19. COSY spectrum (500 MHz) of **OZ2** in $CDCl_3$.

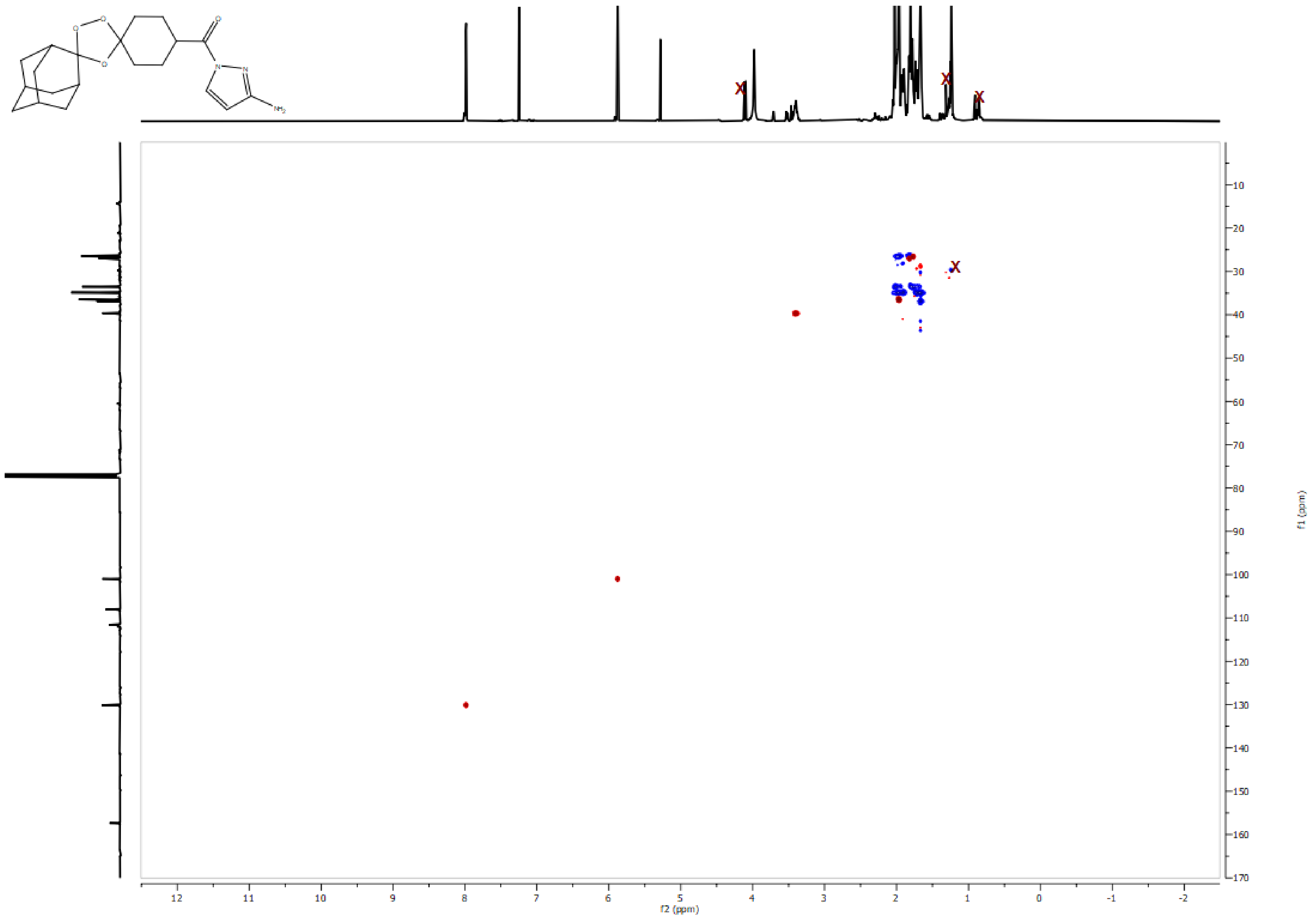

Figure S20. HSQC spectrum (500 MHz) of **OZ2** in $CDCl_3$.

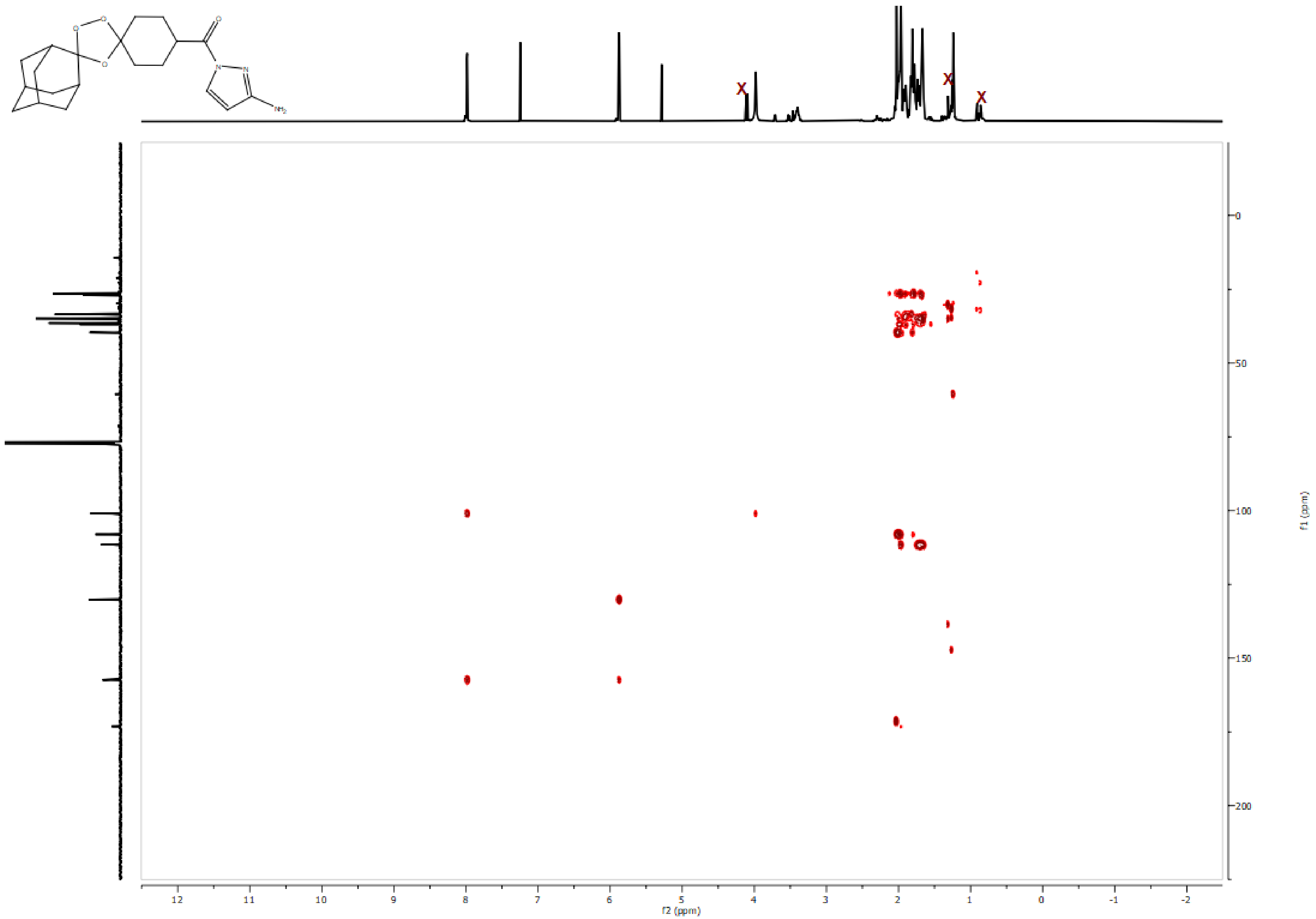

Figure S21. HMBC spectrum (500 MHz) of **OZ2** in $CDCl_3$.

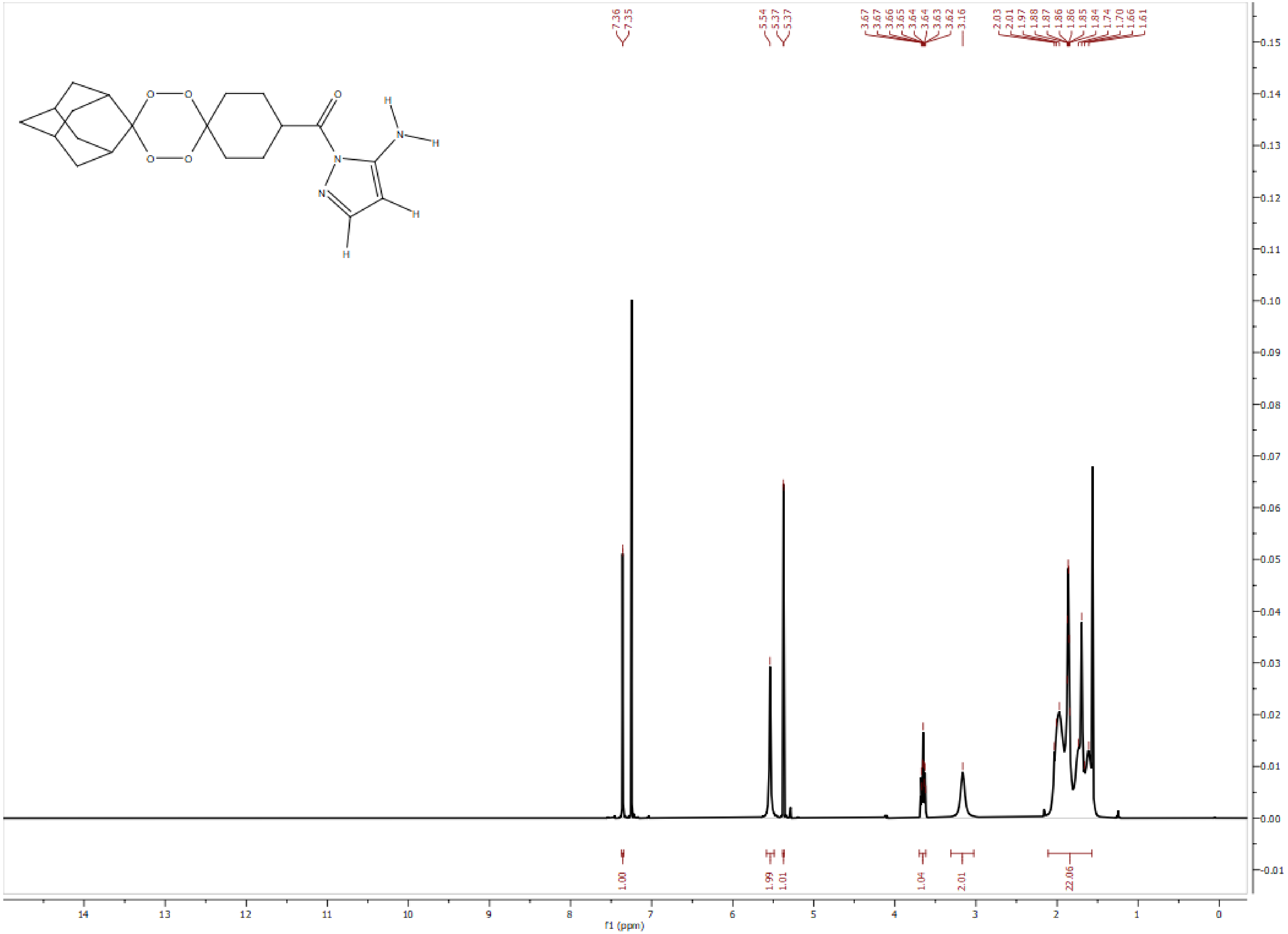

Figure S22. ^{1}H NMR spectrum (500 MHz) of **T1** in $CDCl_3$.

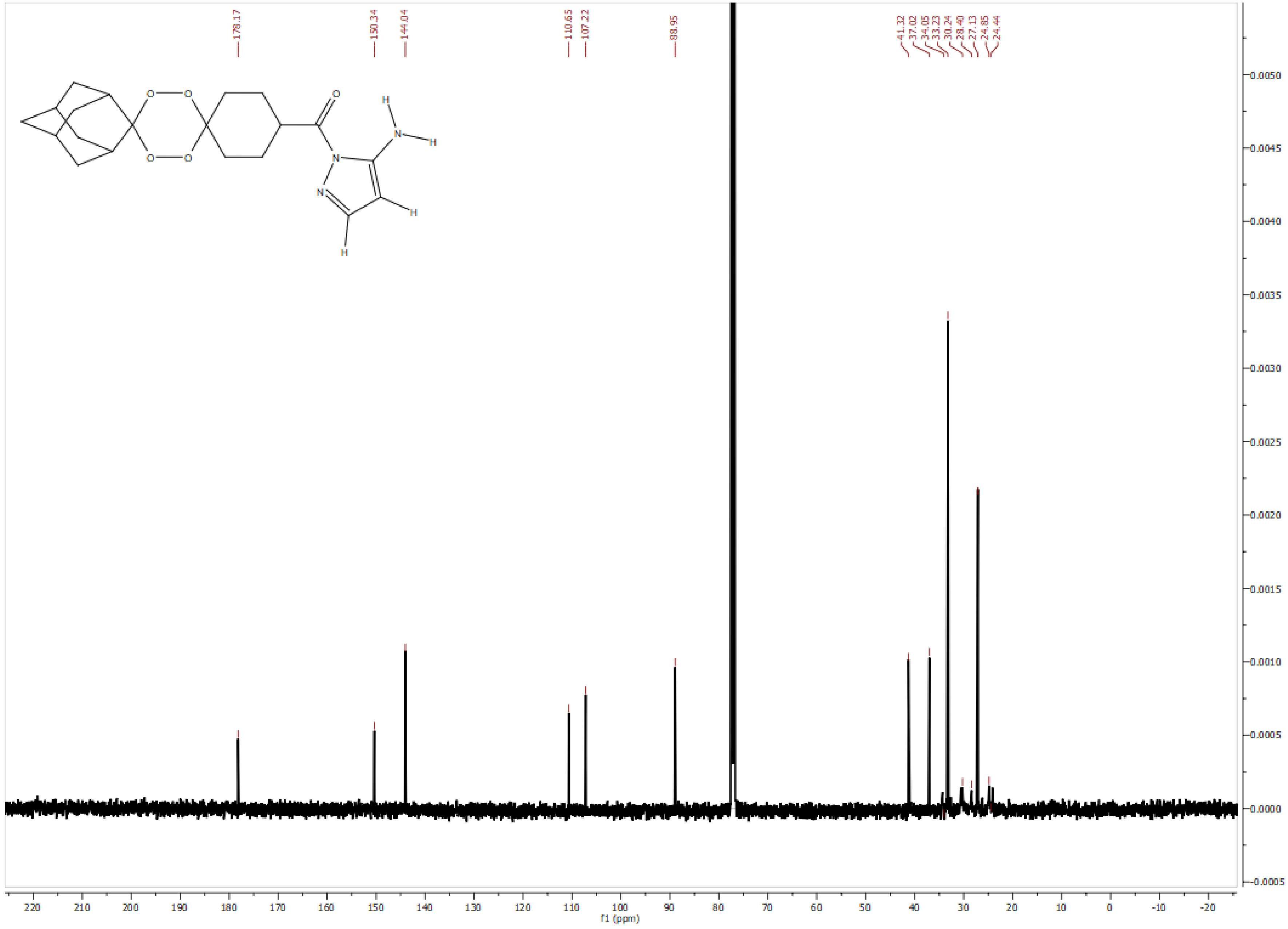

Figure S23. $^{13}C\{^{1}H\}$ NMR spectrum (126 MHz) of **T1** in $CDCl_3$.

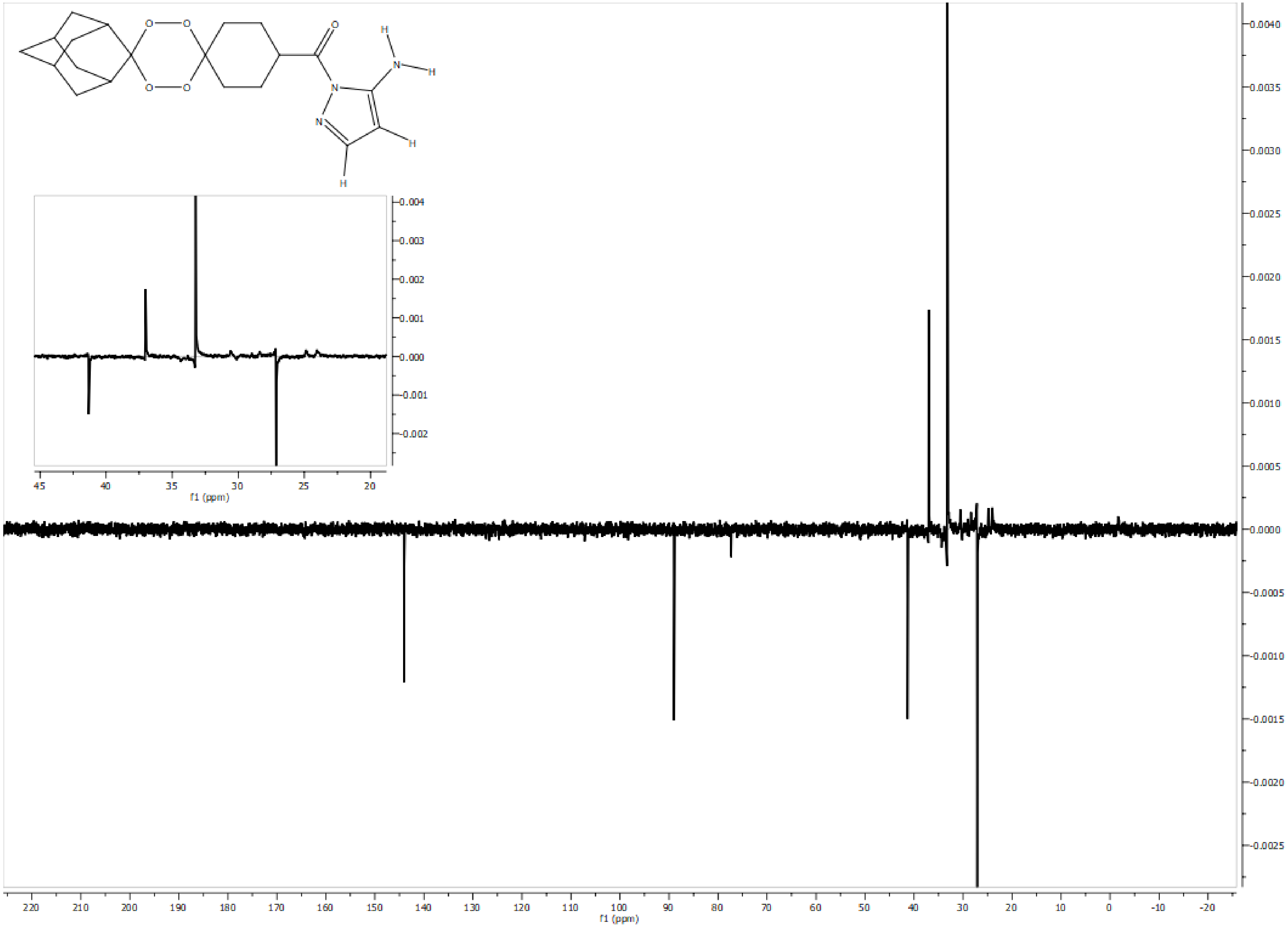

Figure S24. $^{13}C\{^{1}H\}$ DEPT-135 NMR spectrum (126 MHz) of **T1** in $CDCl_3$.

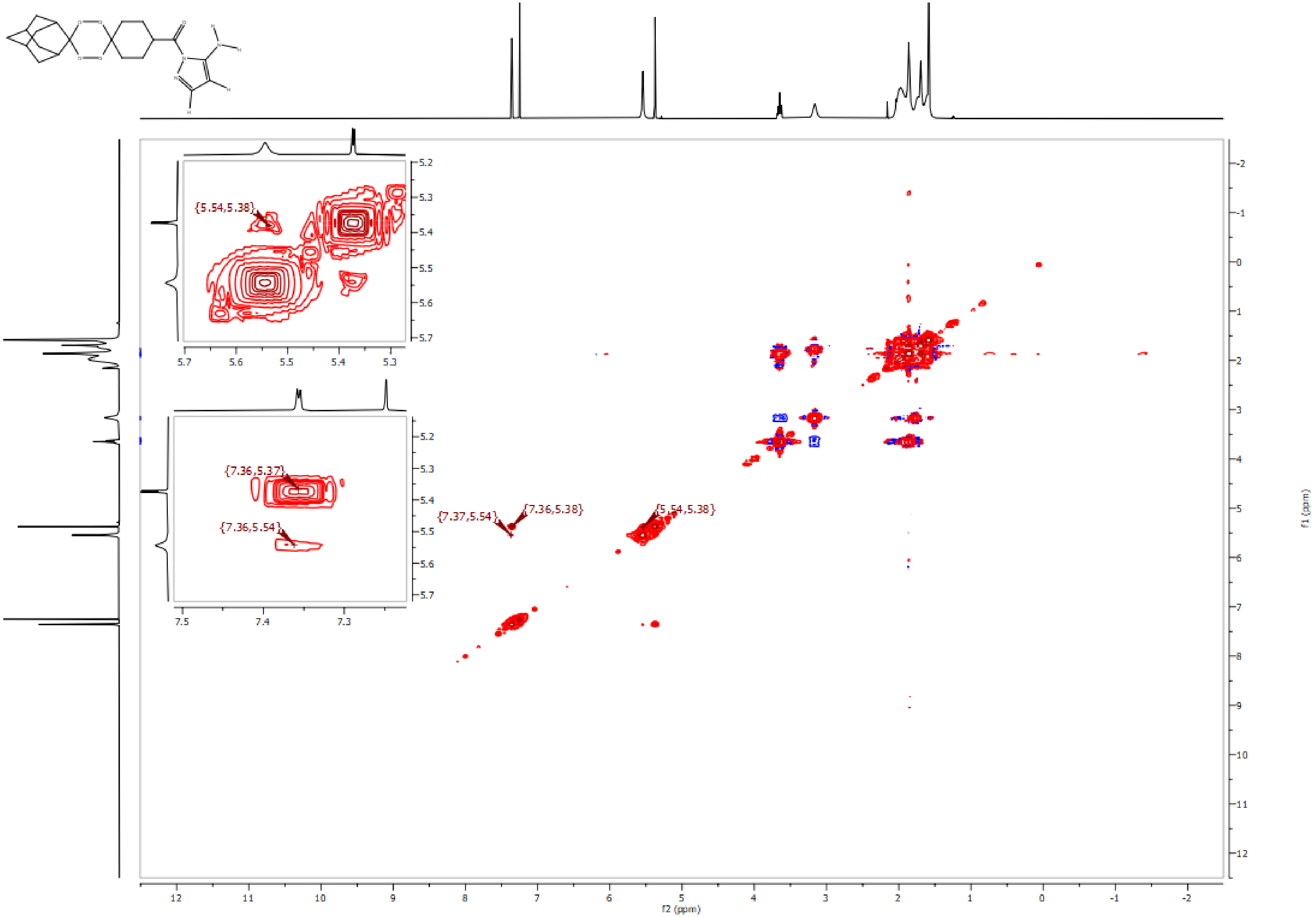

Figure S25. COSY spectrum (500 MHz) of **T1** in $CDCl_3$.

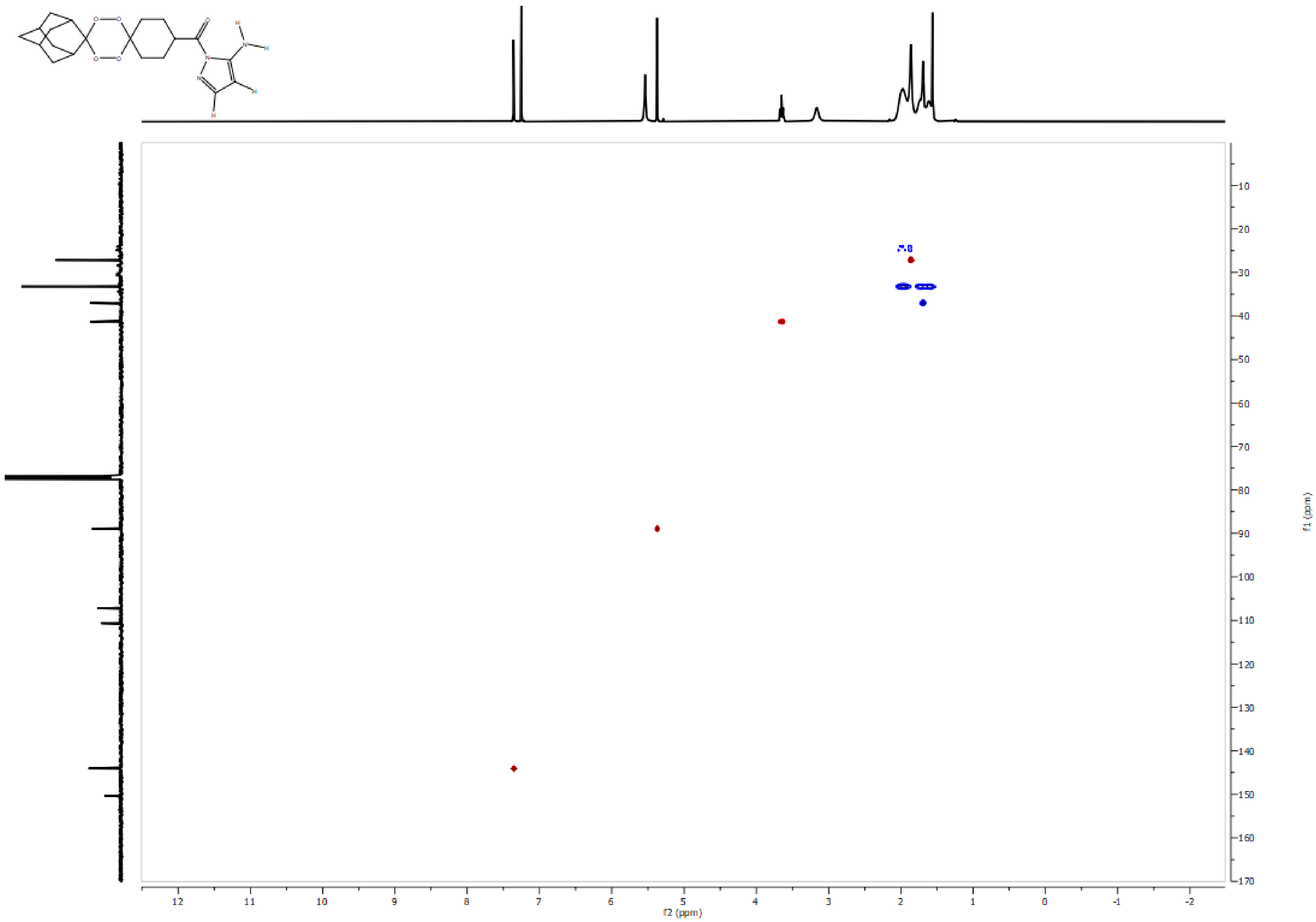

Figure S26. HSQC spectrum (500 MHz) of **T1** in $CDCl_3$.

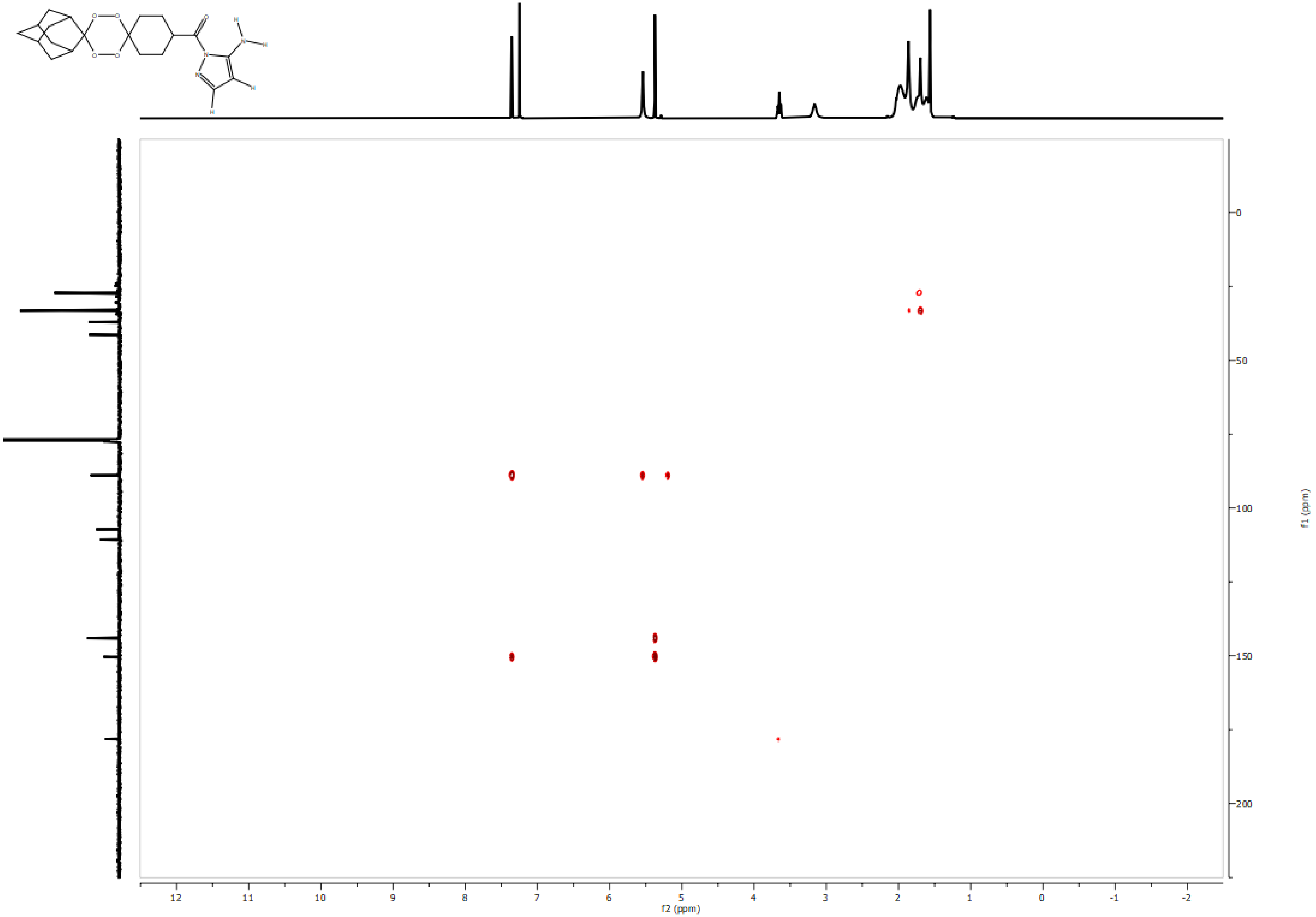

Figure S27. HMBC spectrum (500 MHz) of **T1** in $CDCl_3$.

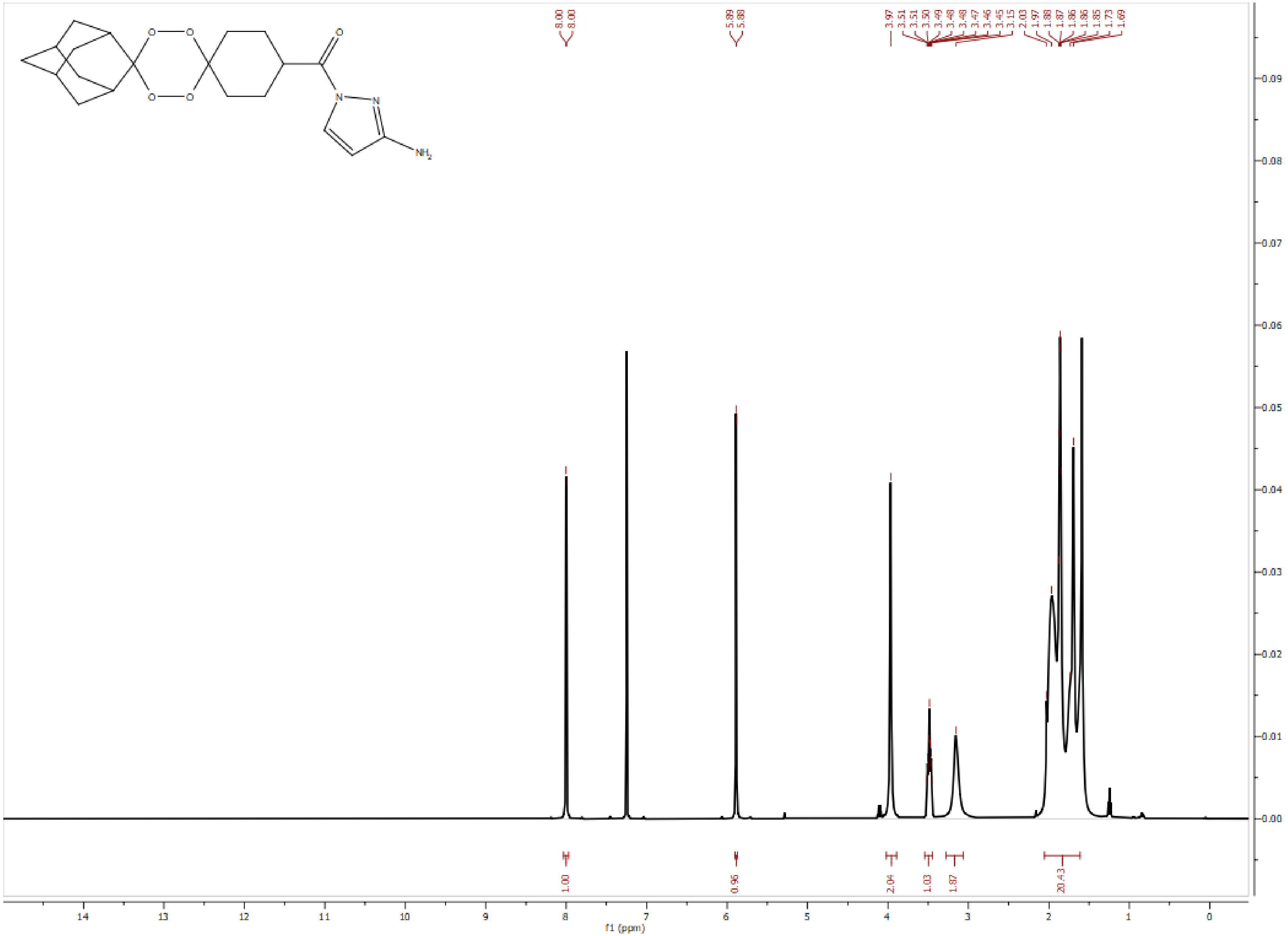

Figure S28. ^{1}H NMR spectrum (500 MHz) of **T2** in $CDCl_3$.

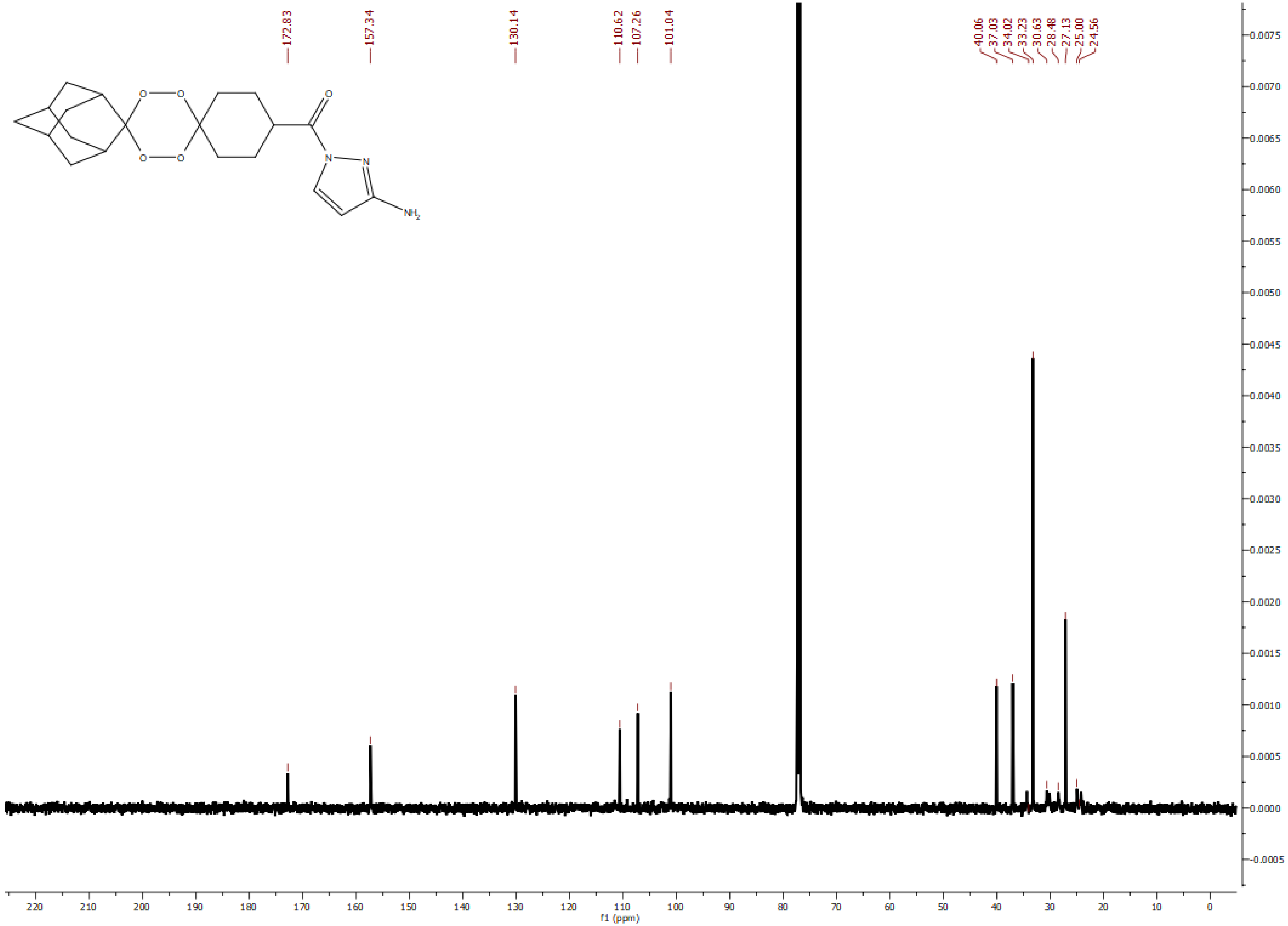

Figure S29. $^{13}C\{^{1}H\}$ NMR spectrum (126 MHz) of **T2** in $CDCl_3$.

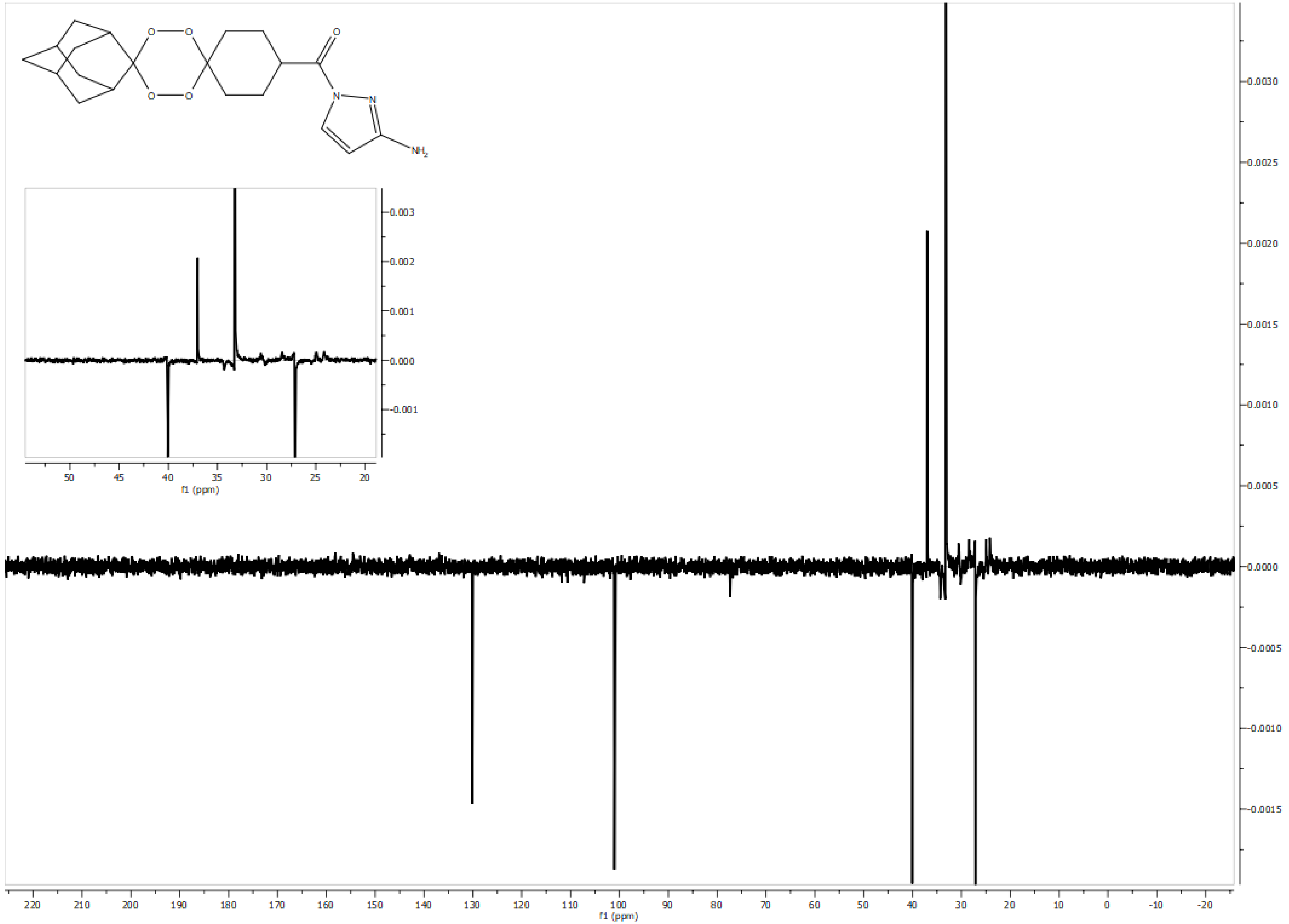

Figure S30. $^{13}C\{^{1}H\}$ DEPT-135 NMR spectrum (126 MHz) of **T2** in $CDCl_3$.

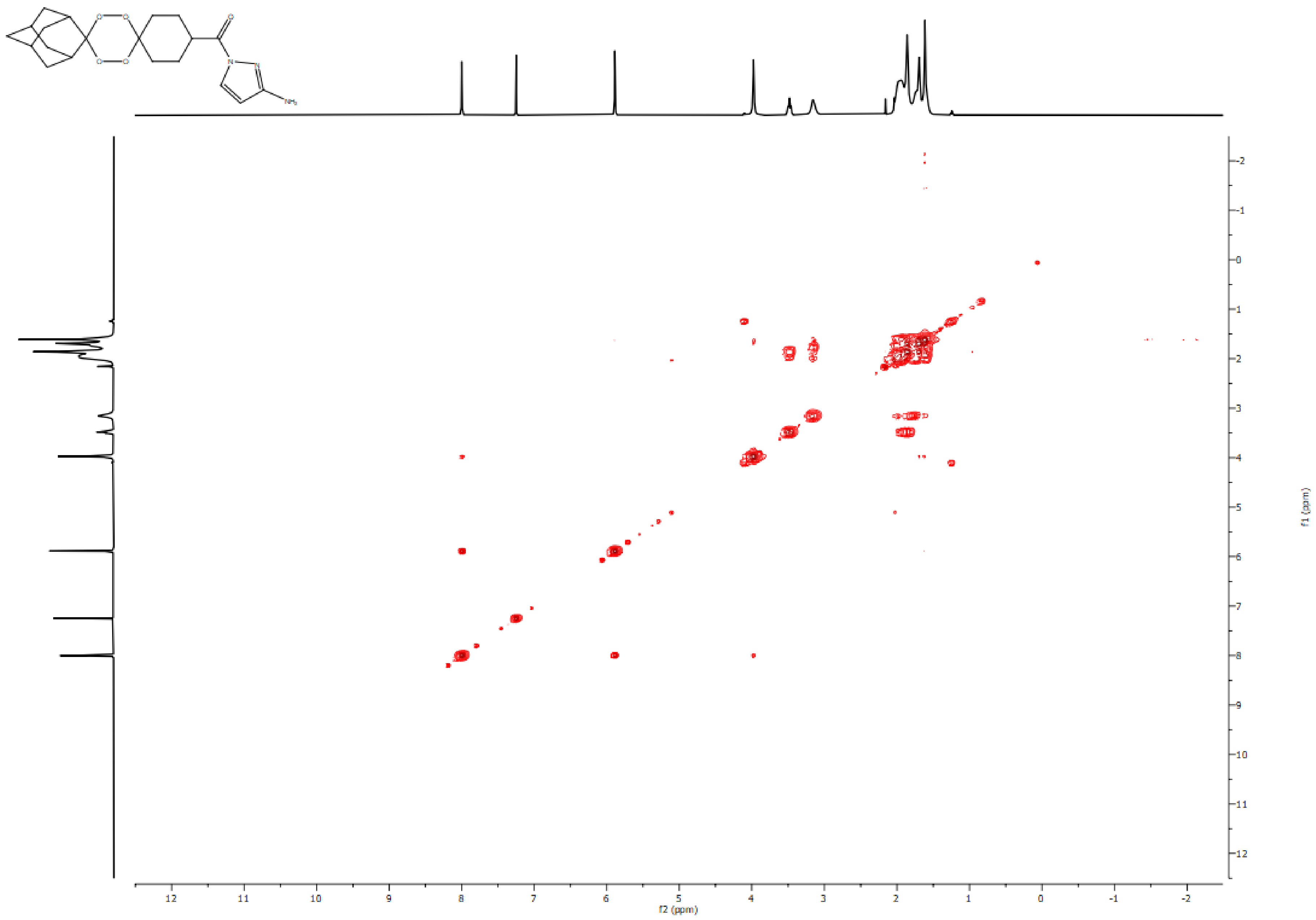

Figure S31. COSY spectrum (500 MHz) of **T2** in $CDCl_3$.

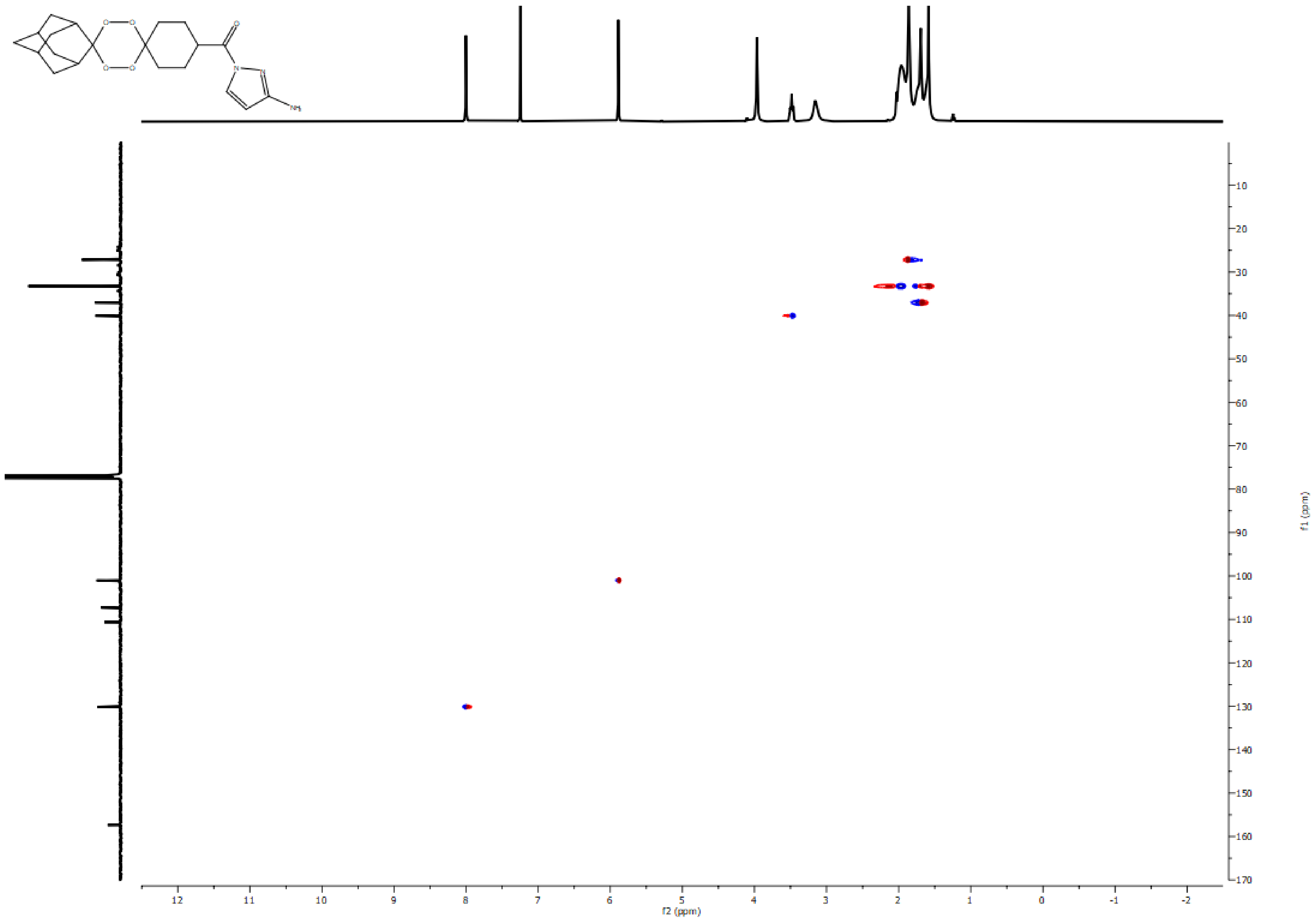

Figure S32. HSQC spectrum (500 MHz) of **T2** in $CDCl_3$.

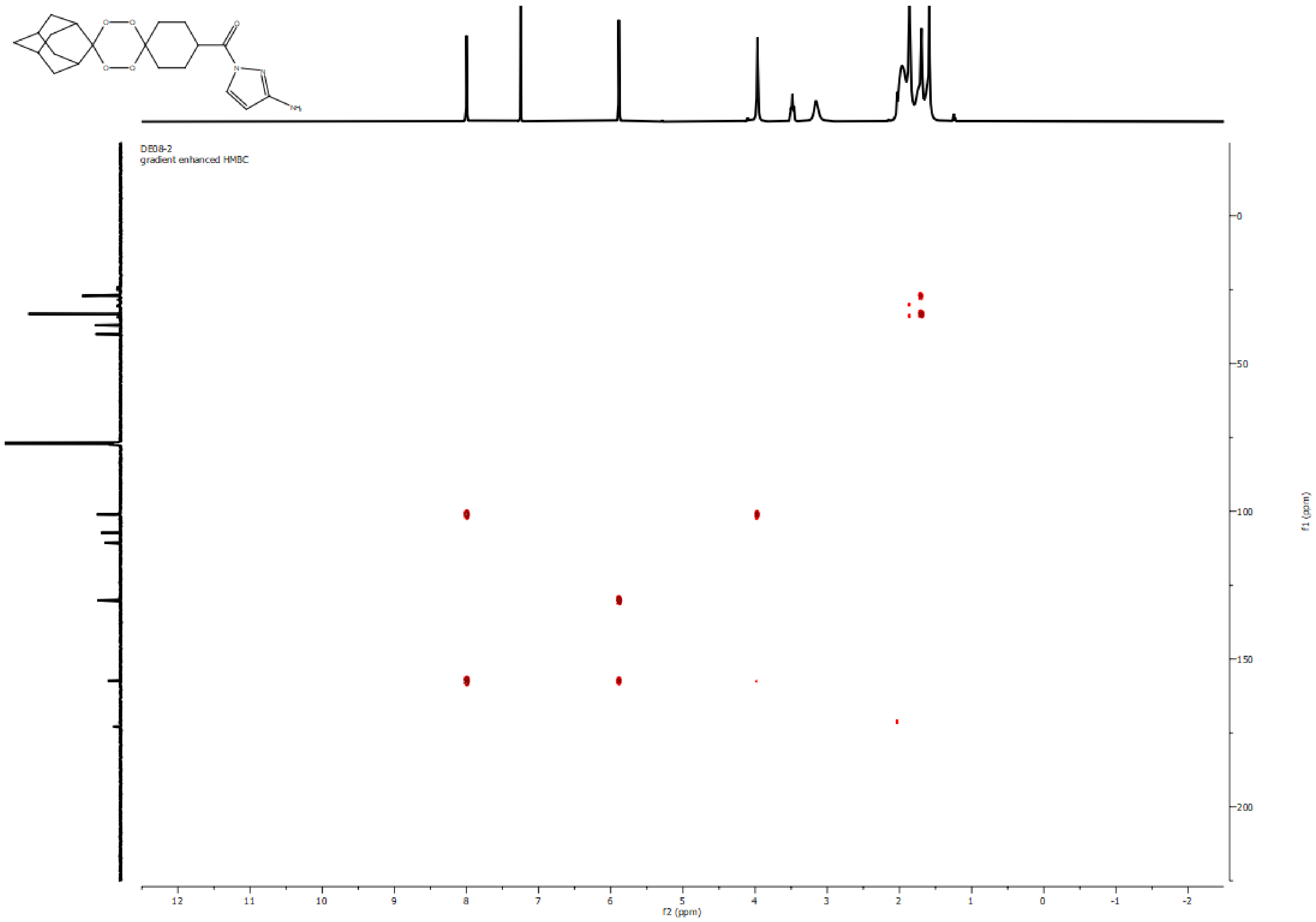

Figure S33. HMBC spectrum (500 MHz) of **T2** in $CDCl_3$.

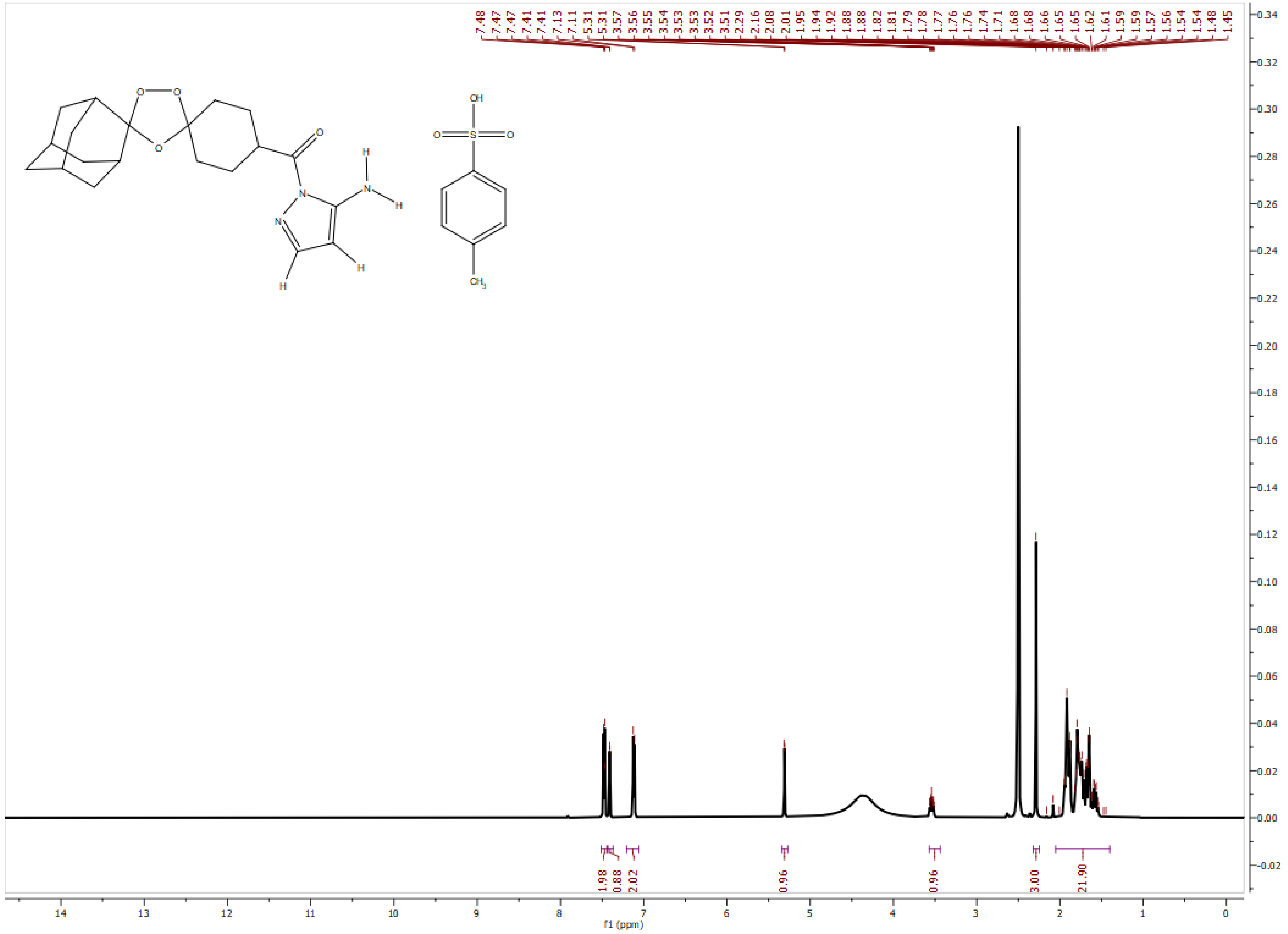

Figure S34. ^{1}H NMR spectrum (500 MHz) of **OZ1•TsOH** in DMSO-d_6.

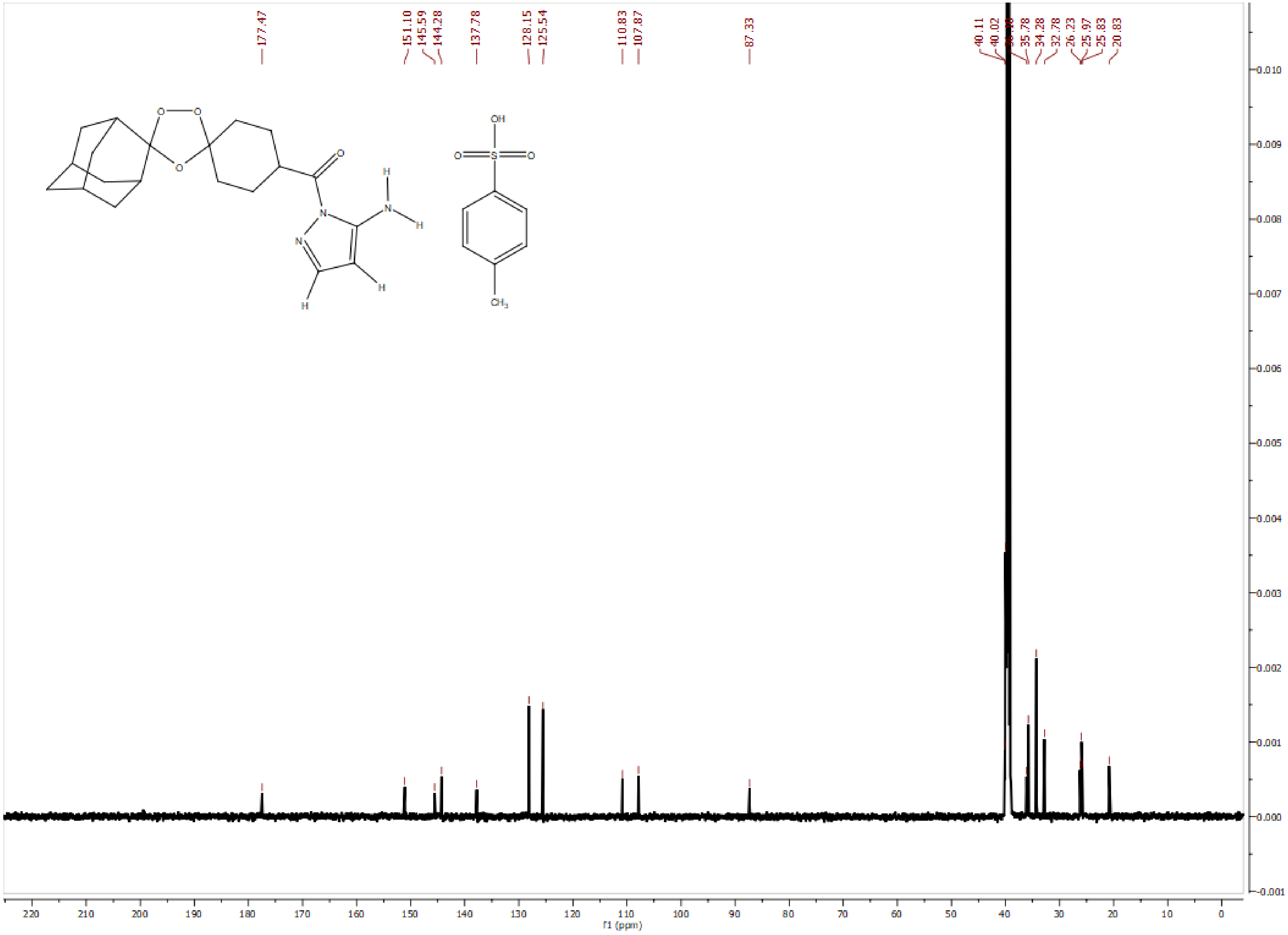

Figure S35. $^{13}C\{^{1}H\}$ NMR spectrum (126 MHz) of **OZ1•TsOH** in DMSO-d_6.

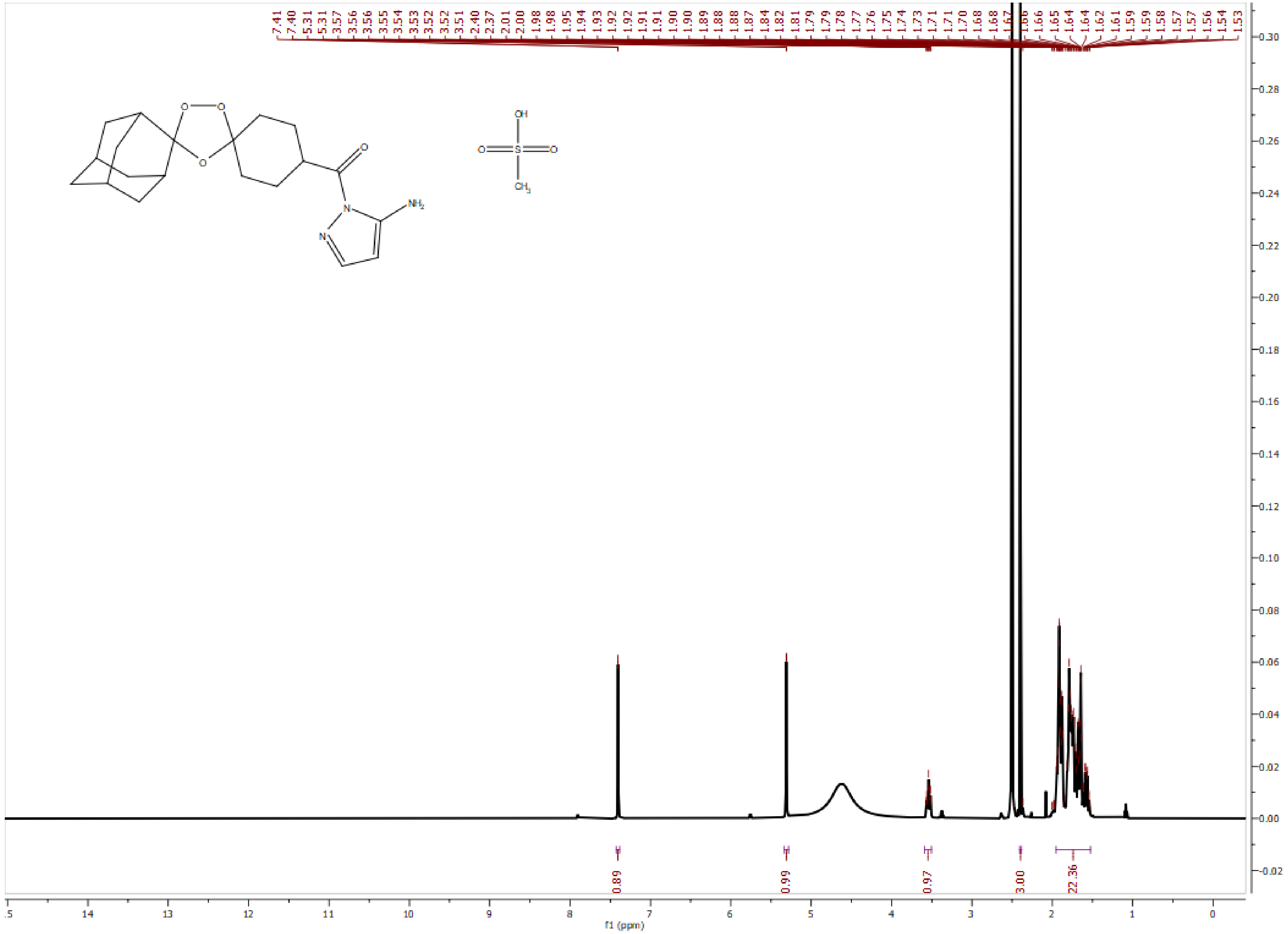

Figure S36. ^{1}H NMR spectrum (500 MHz) of **OZ1•MsOH** in DMSO-d_6.

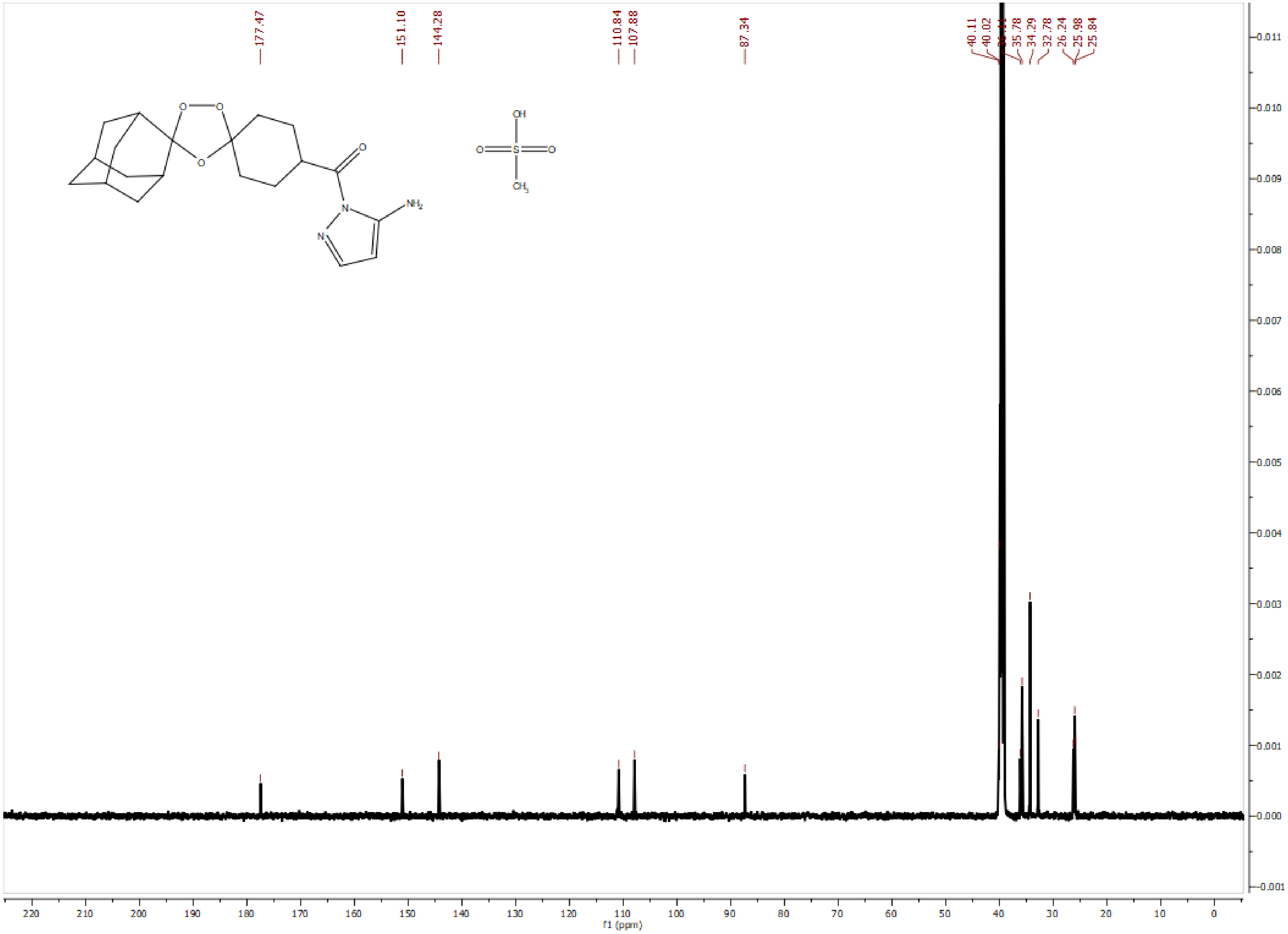

Figure S37. $^{13}C\{^{1}H\}$ NMR spectrum (126 MHz) of **OZ1•MsOH** in DMSO-d_6.

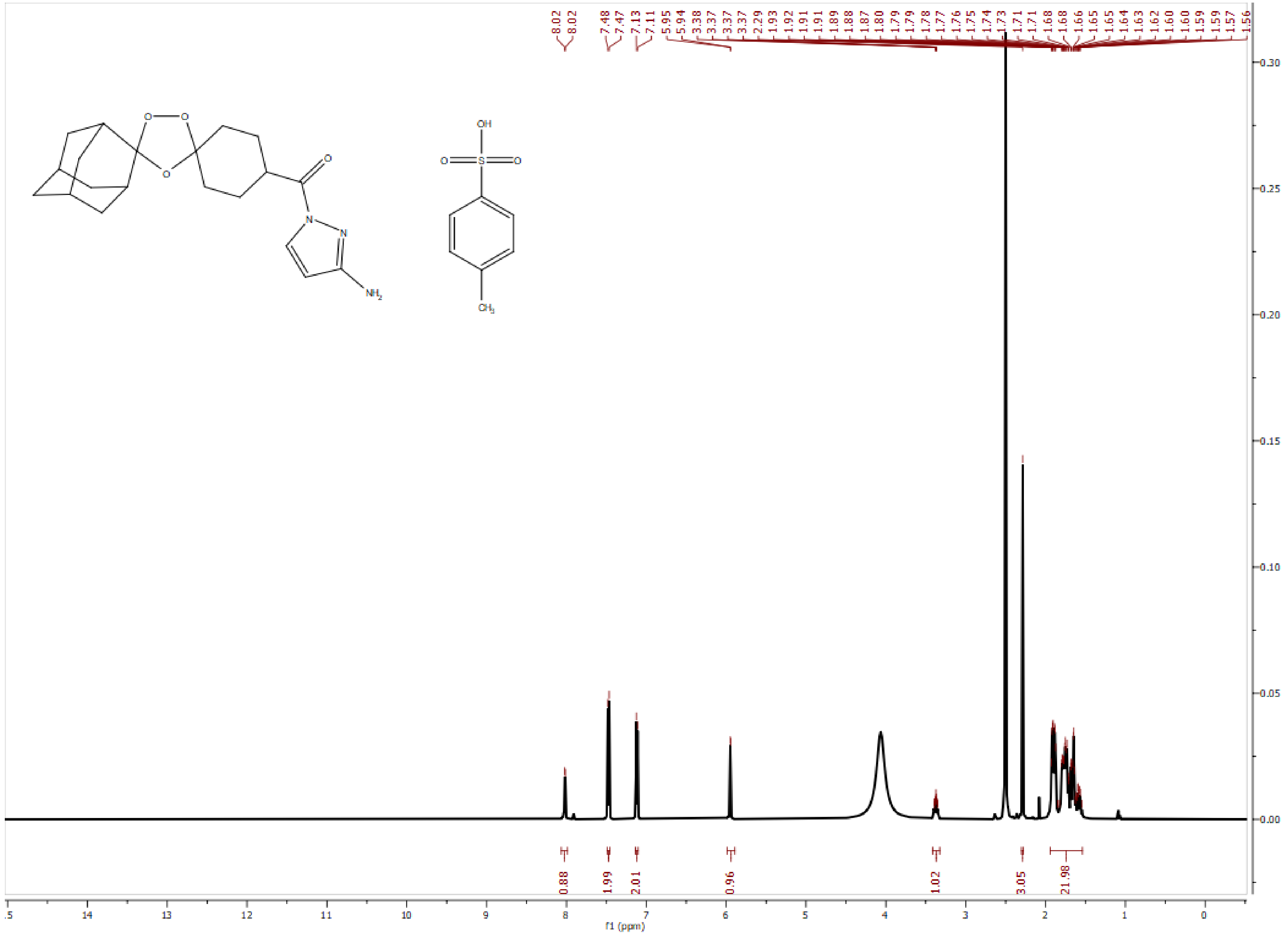

Figure S38. ^{1}H NMR spectrum (500 MHz) of **OZ2•TsOH** in DMSO-d_6.

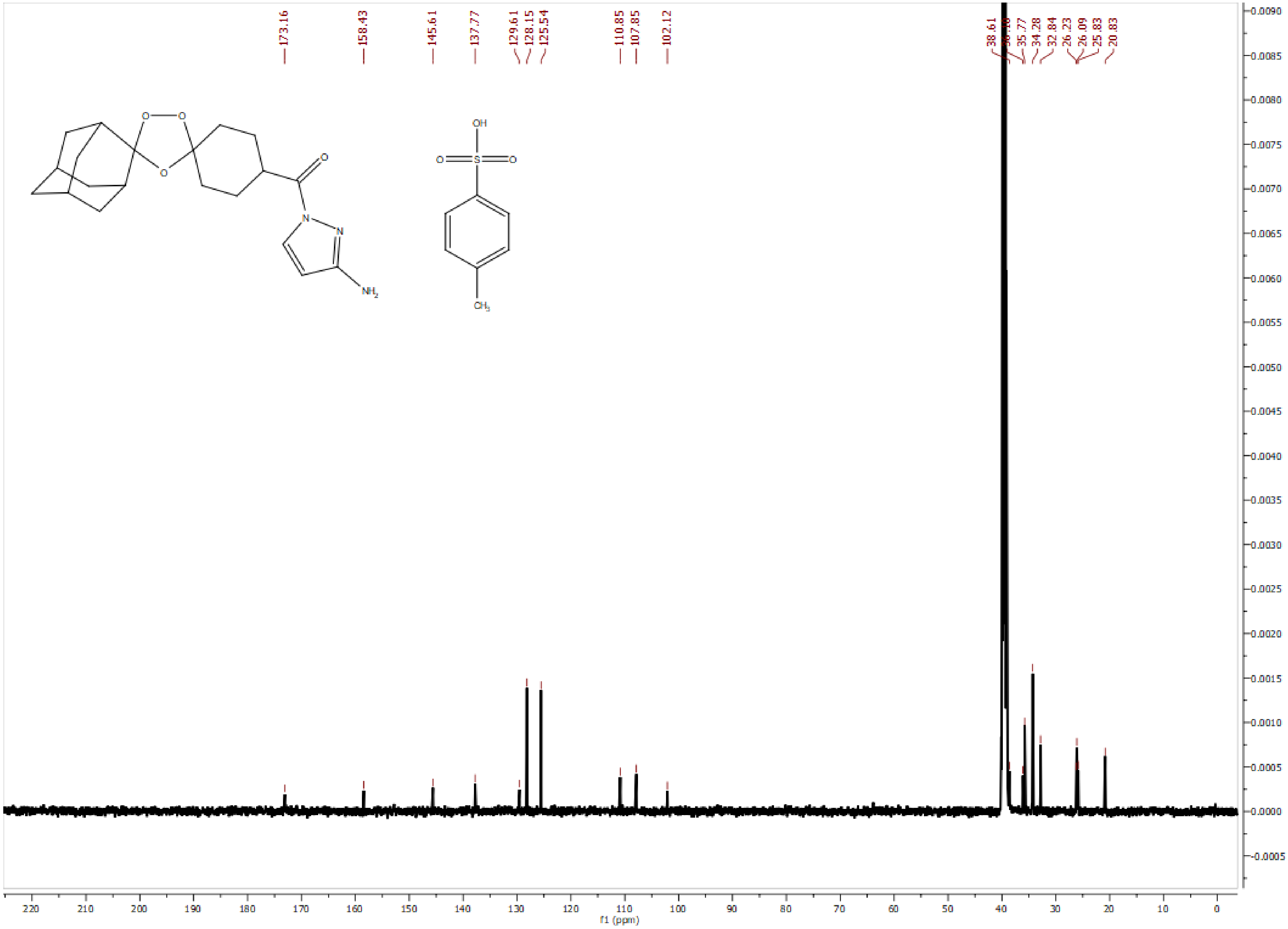

Figure S39. $^{13}C\{^{1}H\}$ NMR spectrum (126 MHz) of **OZ2•TsOH** in DMSO-d_6.

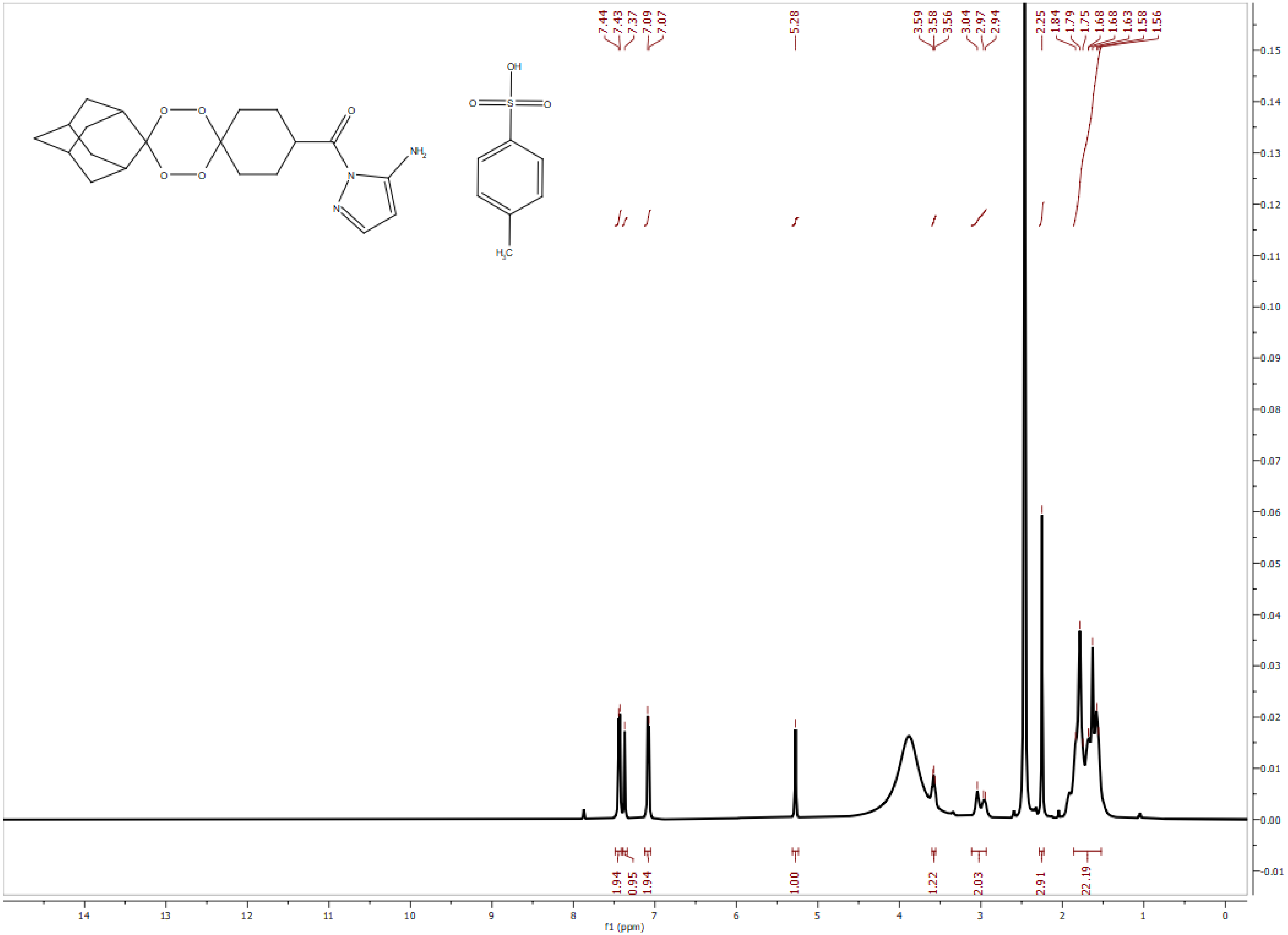

Figure S40. ^{1}H NMR spectrum (500 MHz) of **T1•TsOH** in DMSO-d_6.

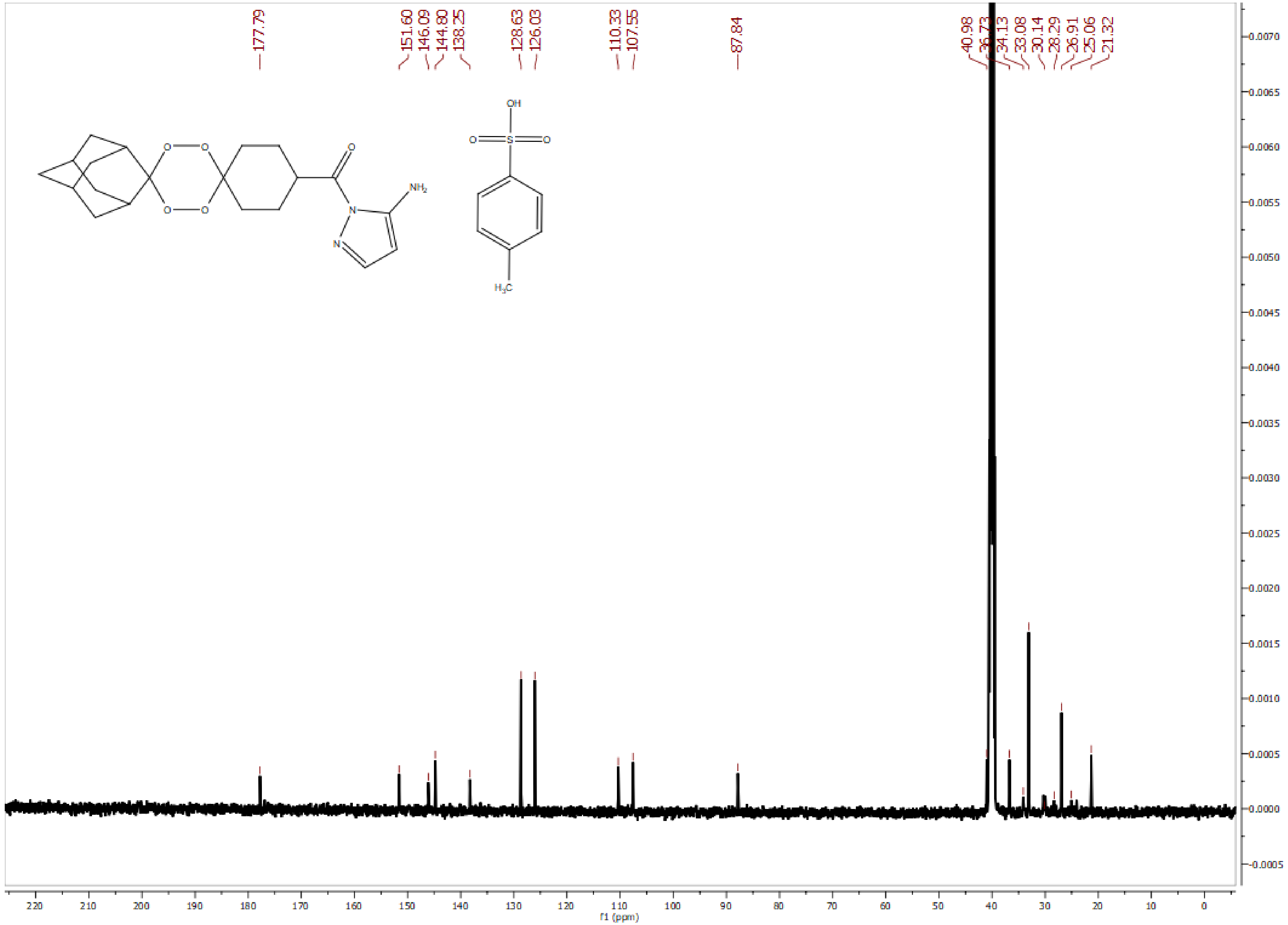

Figure S41. $^{13}C\{^{1}H\}$ NMR spectrum (126 MHz) of **T1•TsOH** in DMSO-d_6.

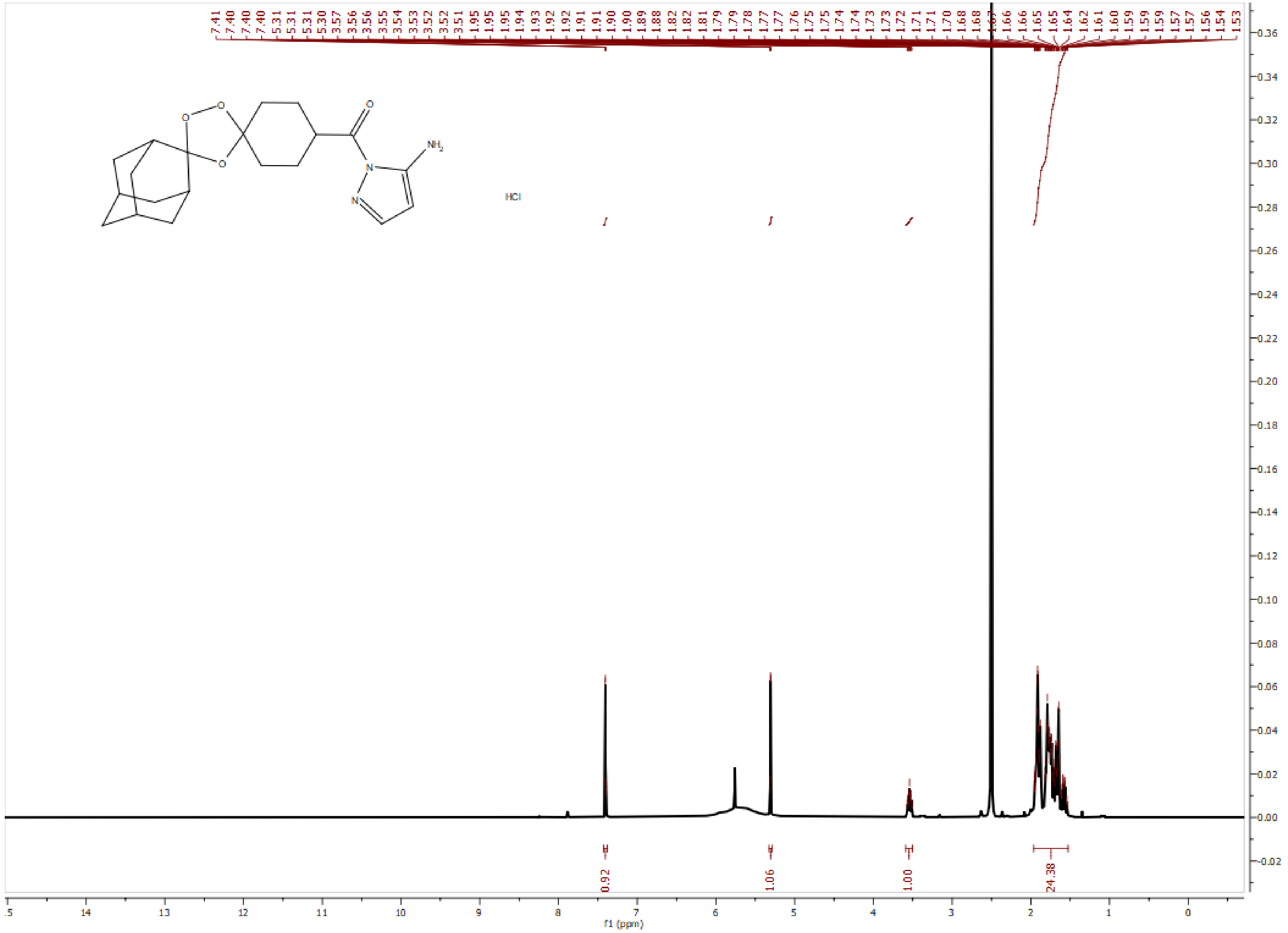

Figure S42. ^{1}H NMR spectrum (500 MHz) of **OZ1•HCl** in DMSO-d_6.

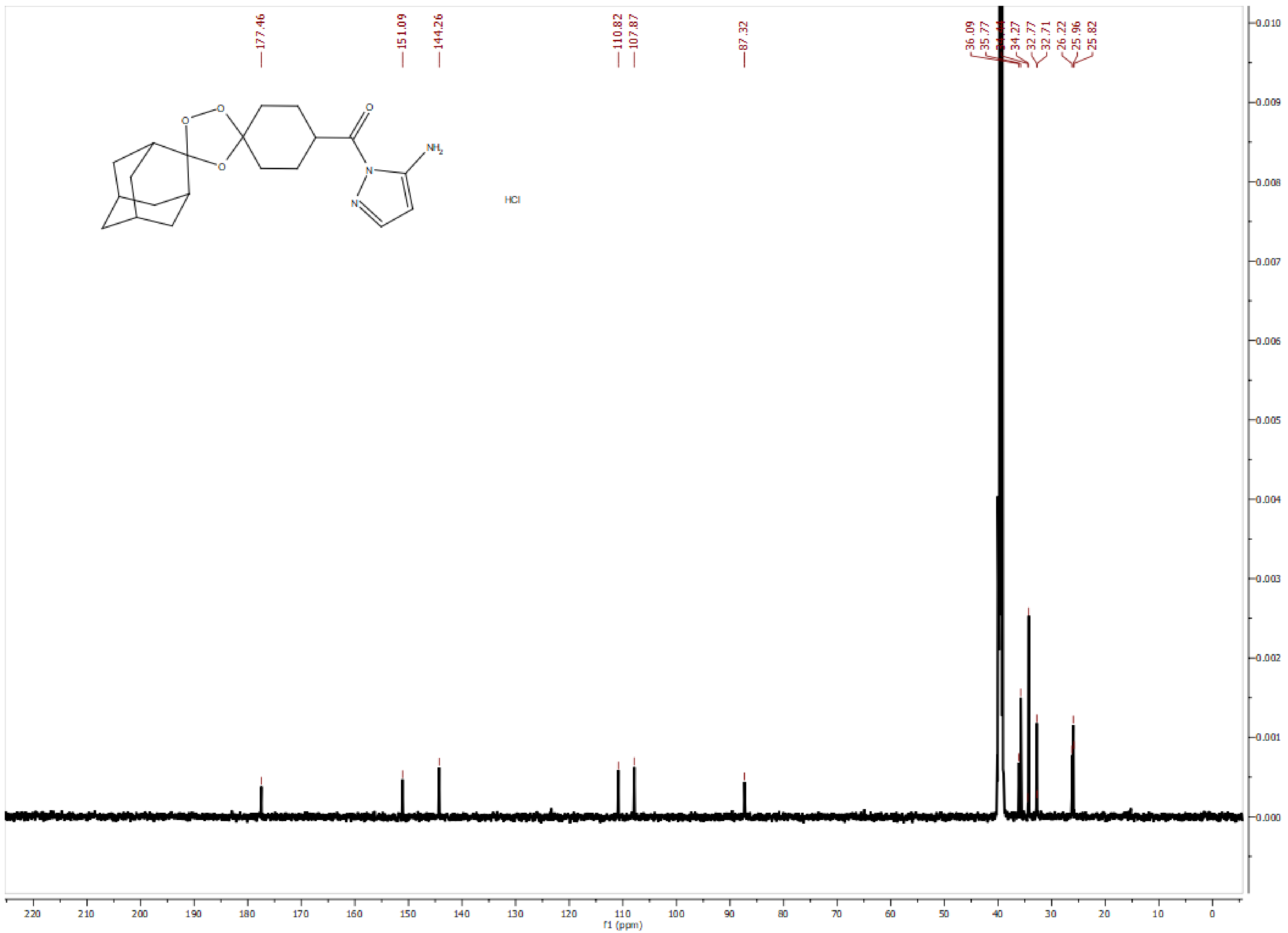

Figure S43. $^{13}C\{^{1}H\}$ NMR spectrum (126 MHz) of **OZ1•HCl** in DMSO-d_6.

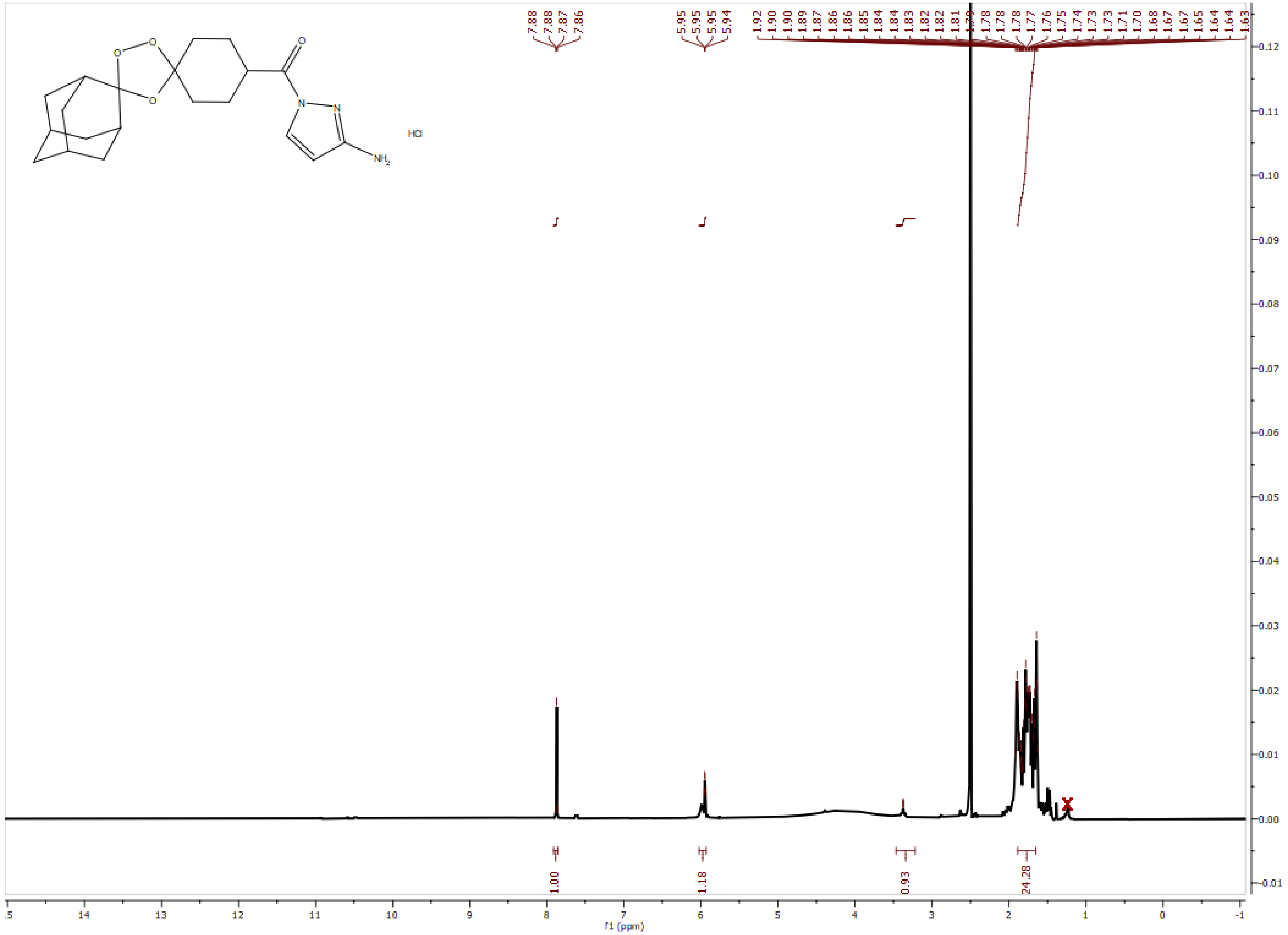

Figure S44. ^{1}H NMR spectrum (500 MHz) of **OZ2•HCl** in DMSO-d_6.

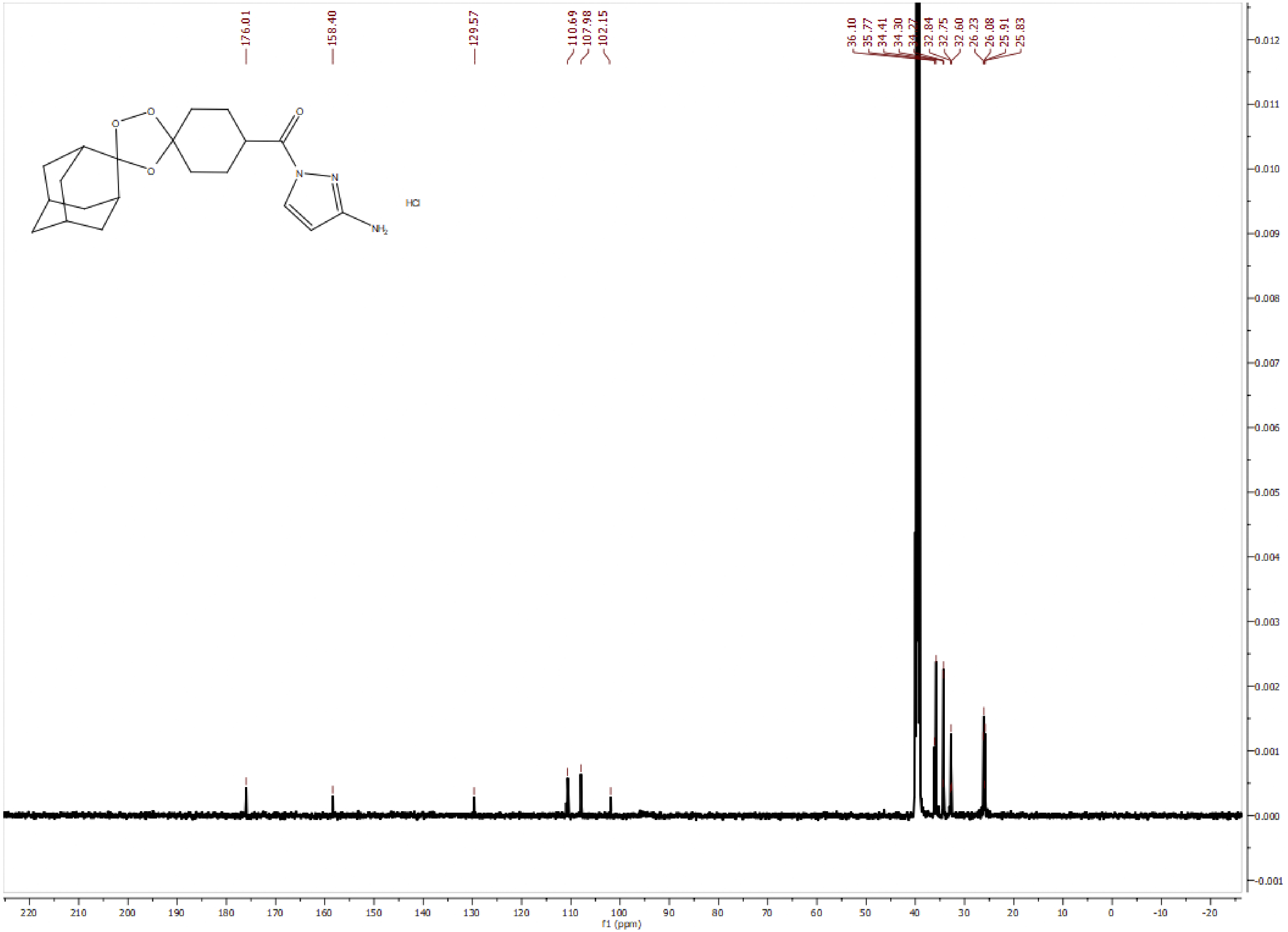

Figure S45. $^{13}C\{^{1}H\}$ NMR spectrum (126 MHz) of **OZ2•HCl** in DMSO-d_6.

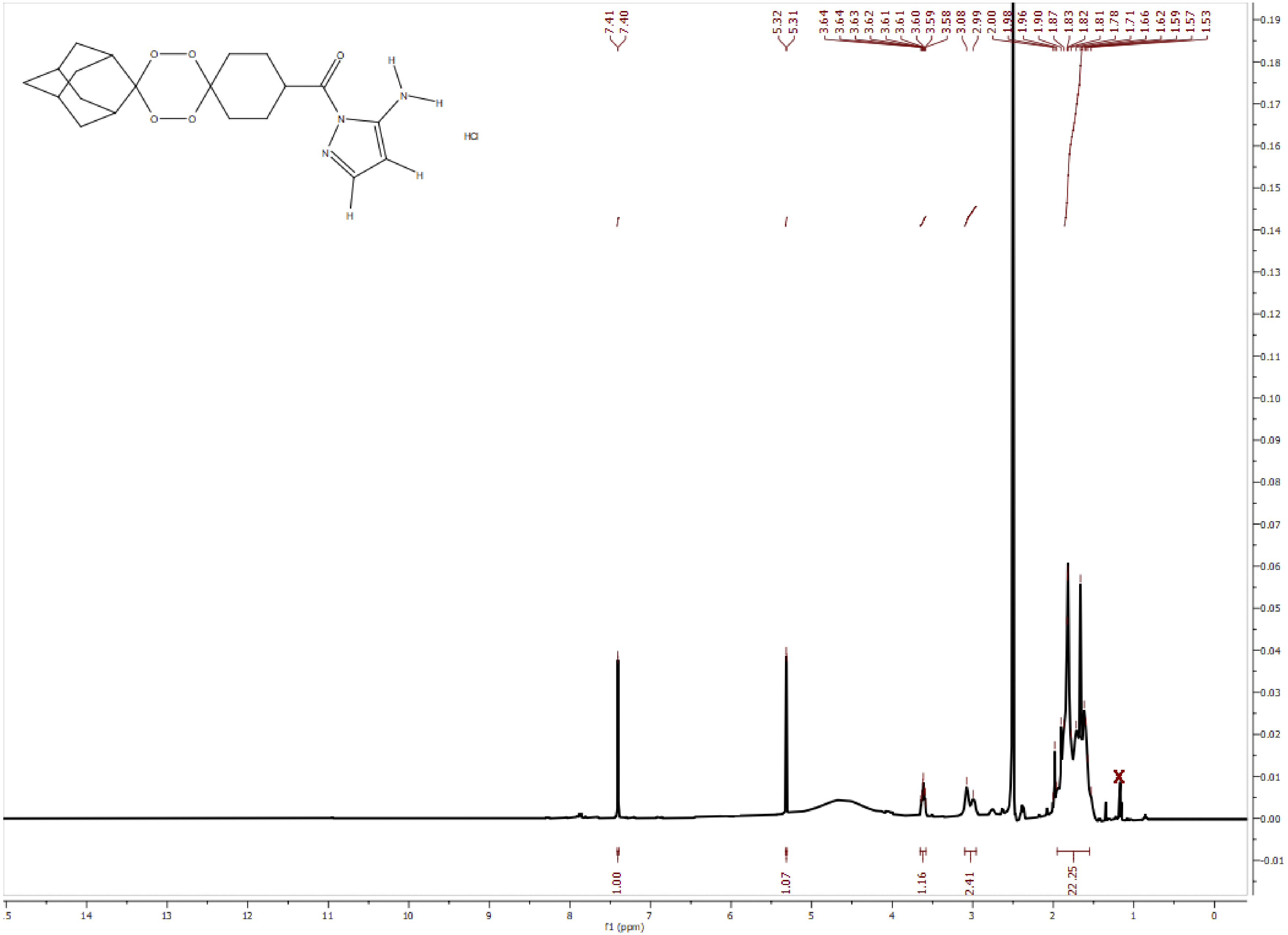

Figure S46. ^{1}H NMR spectrum (500 MHz) of **T1•HCl** in DMSO-d_6.

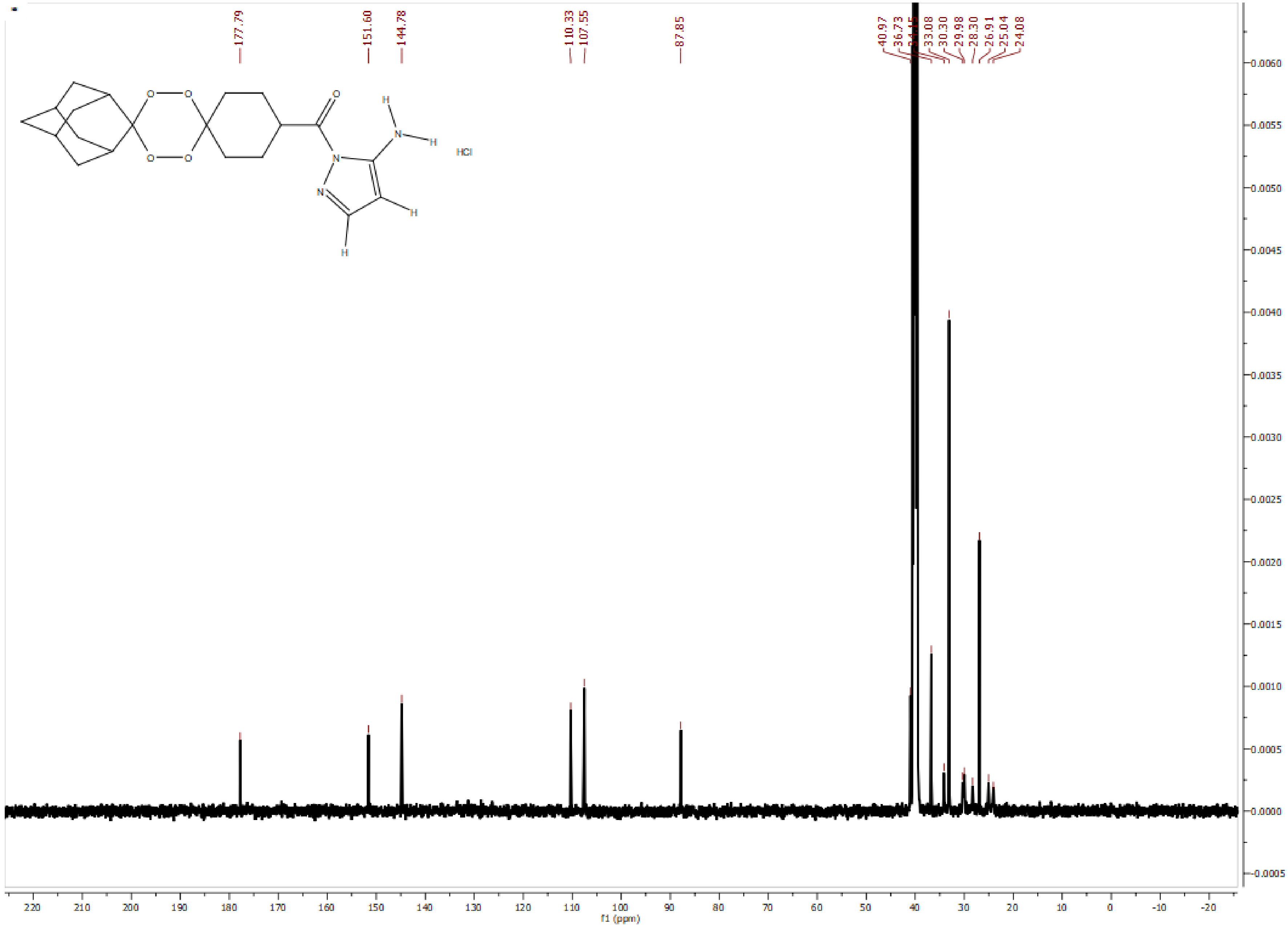

Figure S47. $^{13}C\{^{1}H\}$ NMR spectrum (126 MHz) of **T1•HCl** in DMSO-d_6.

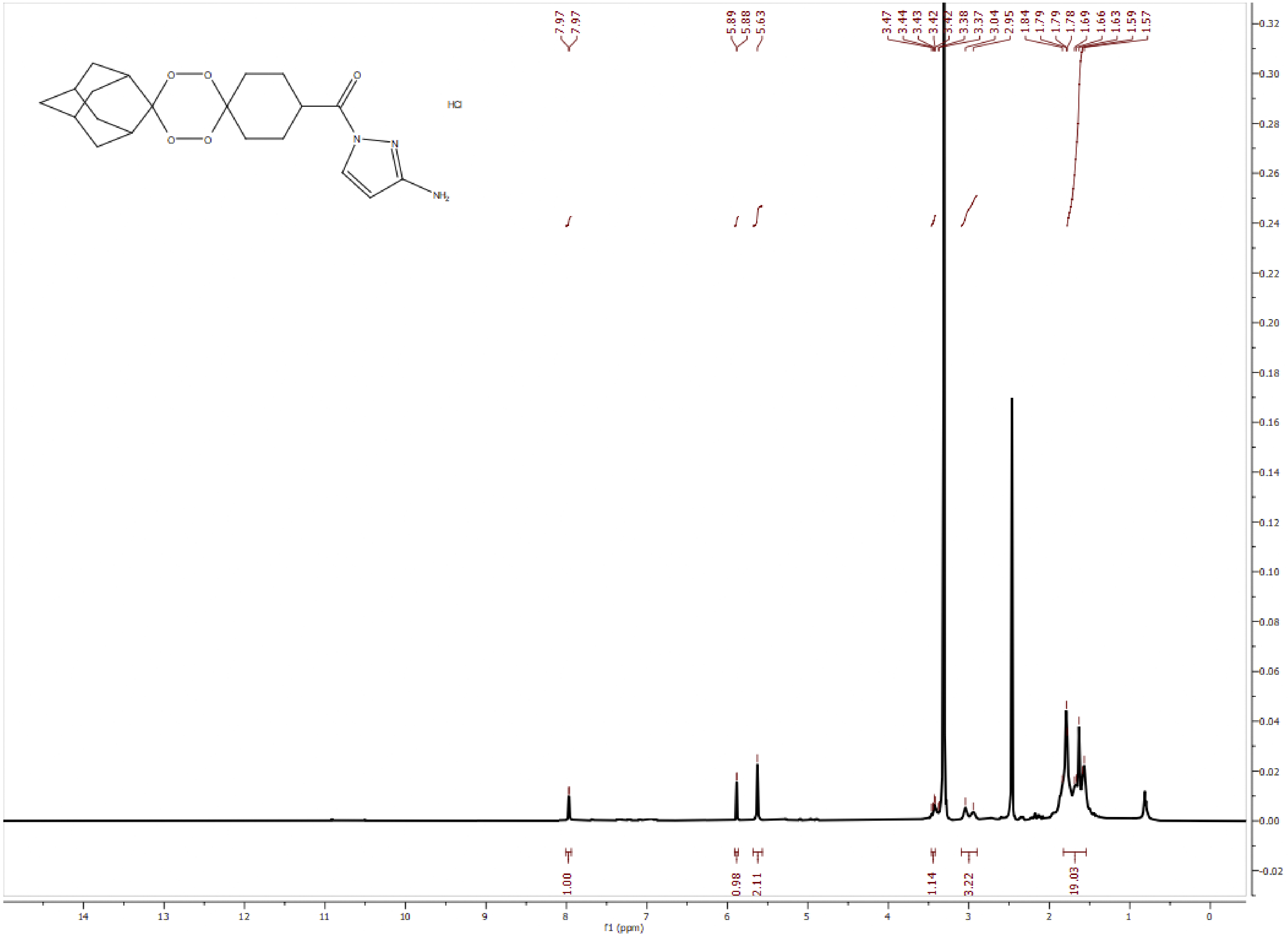

Figure S48. ^{1}H NMR spectrum (500 MHz) of **T2•HCl** in DMSO-d_6.

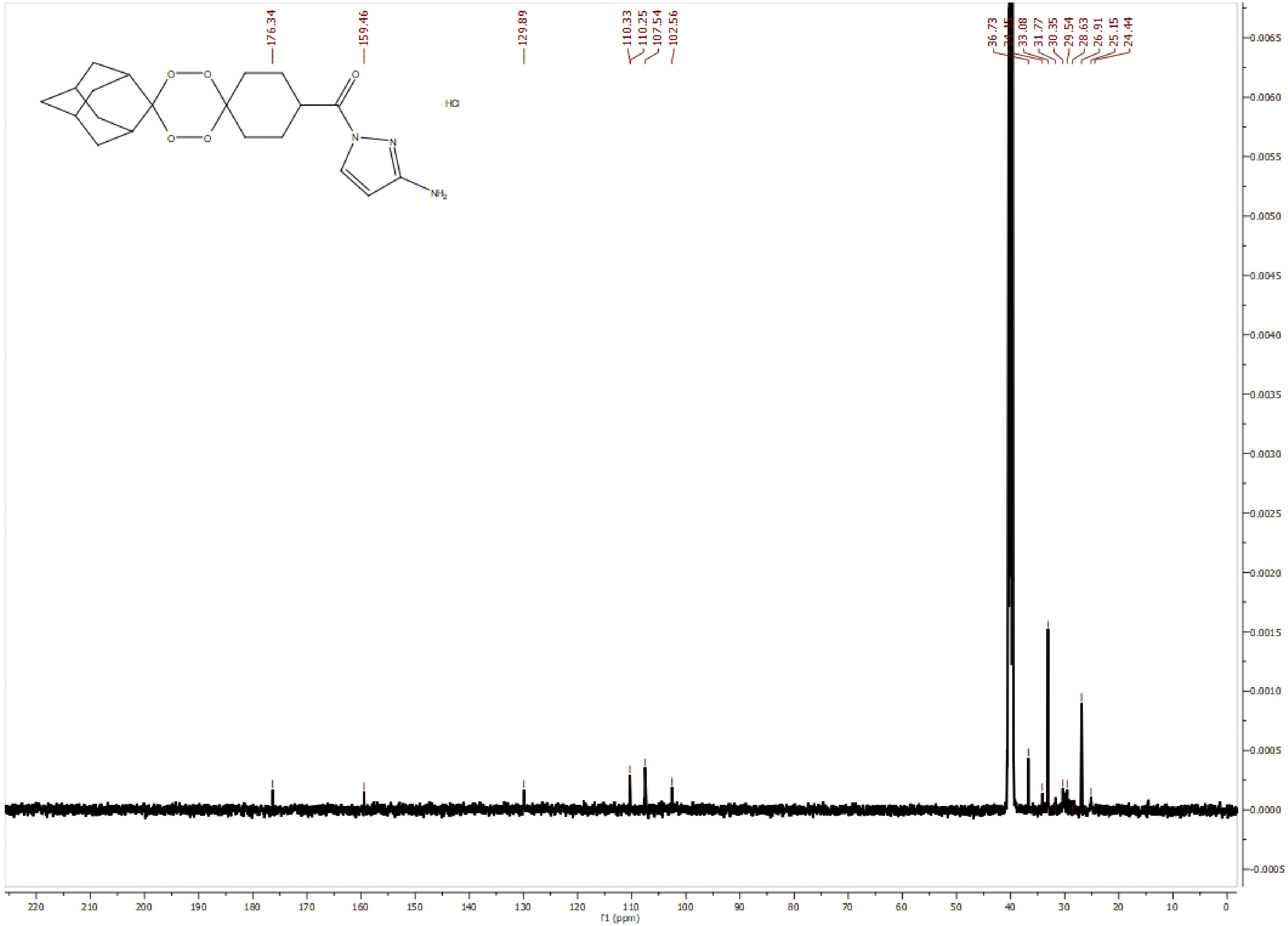

Figure S49. ^{1}H NMR spectrum (500 MHz) of **T2•HCl** in DMSO-d_6.

S2. HRMS spectra of the synthesised compounds

Electrospray ionization mass spectrum in positive-ion mode (HRMS-ESI$^+$) of **compound 1o**

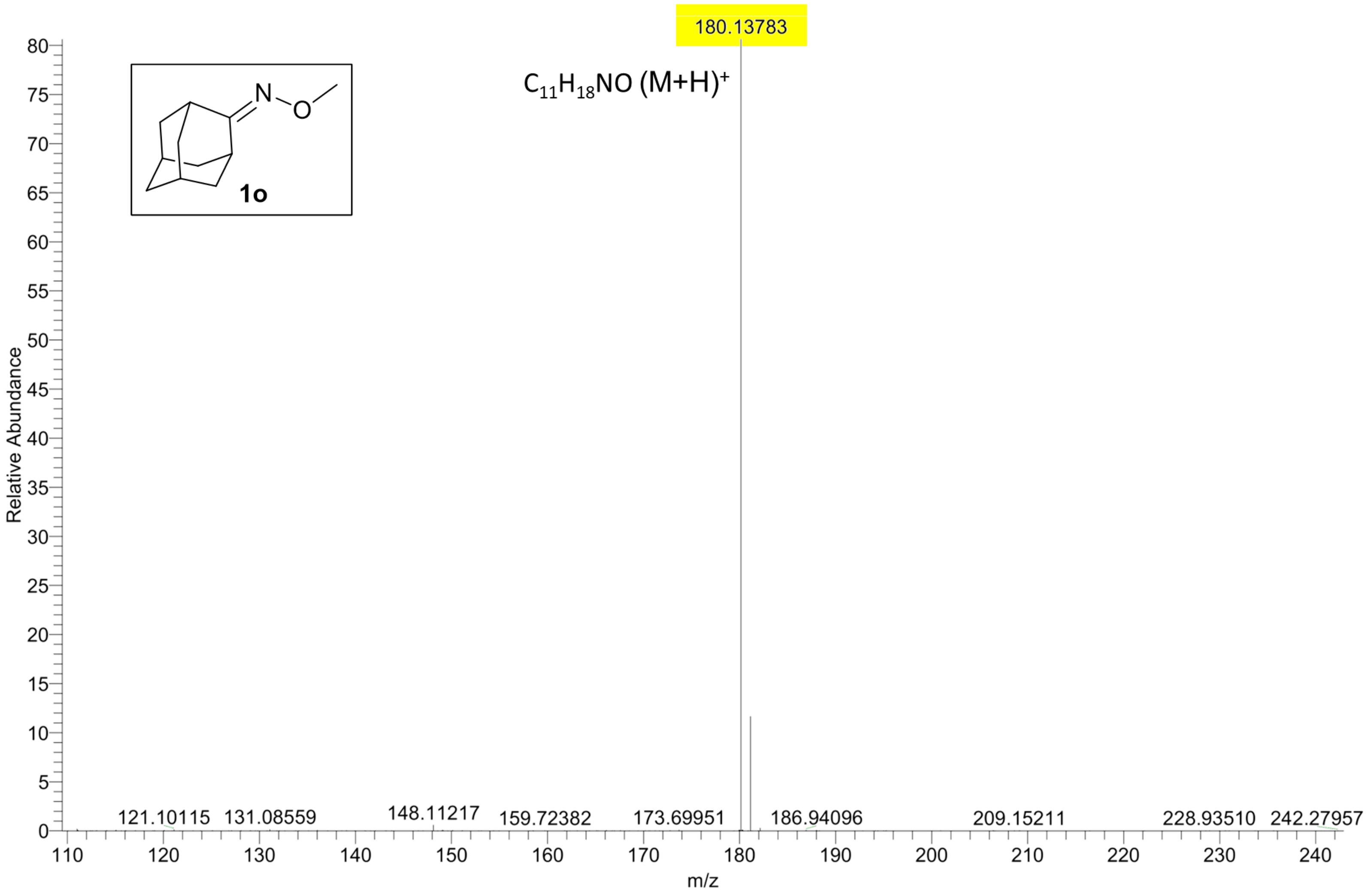

Electrospray ionization mass spectrum in positive-ion mode (HRMS-ESI$^+$) of **compound 2o**

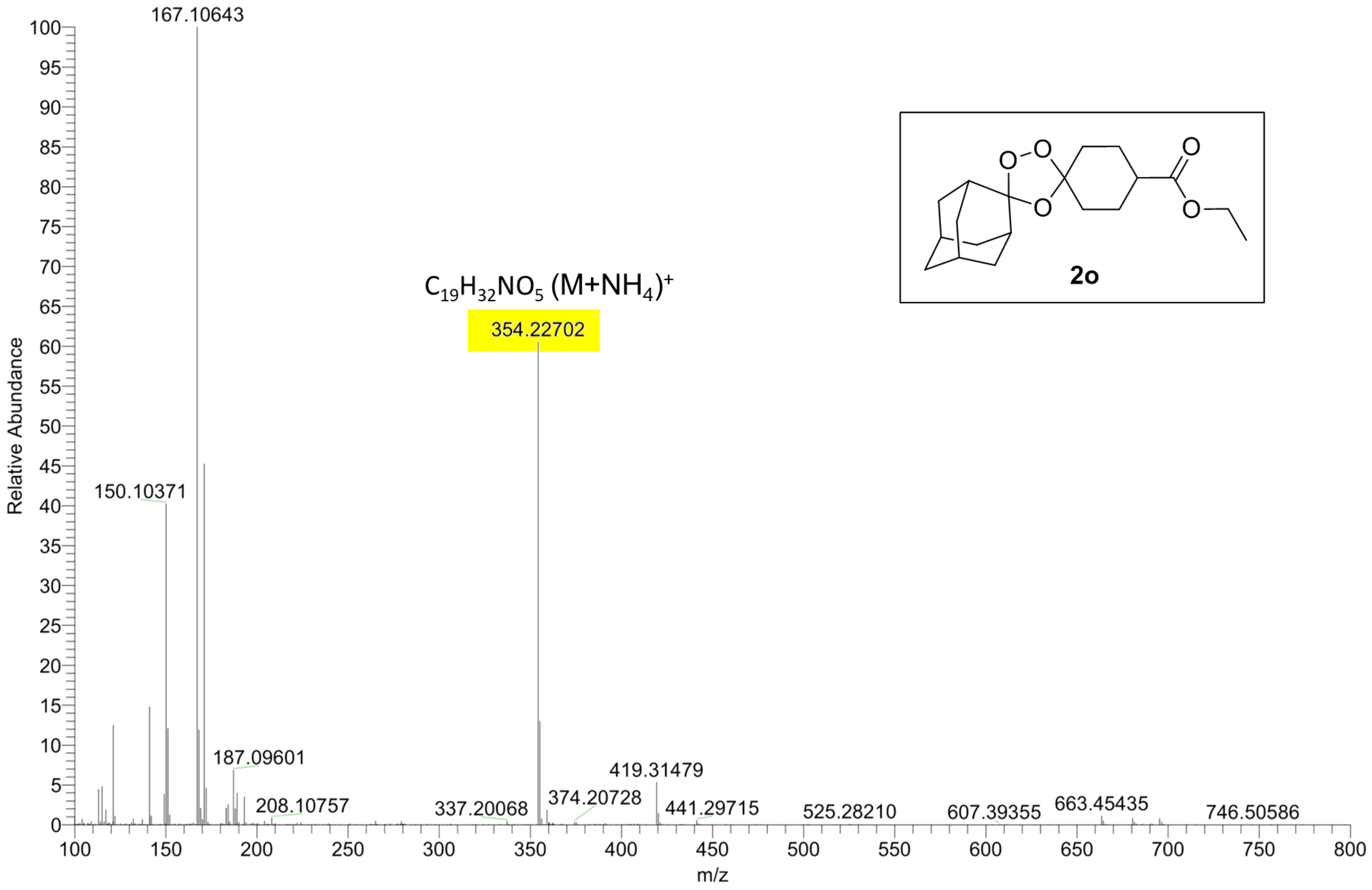

Electrospray ionization mass spectrum in negative-ion mode (HRMS-ESI$^-$) of **compound 3o**

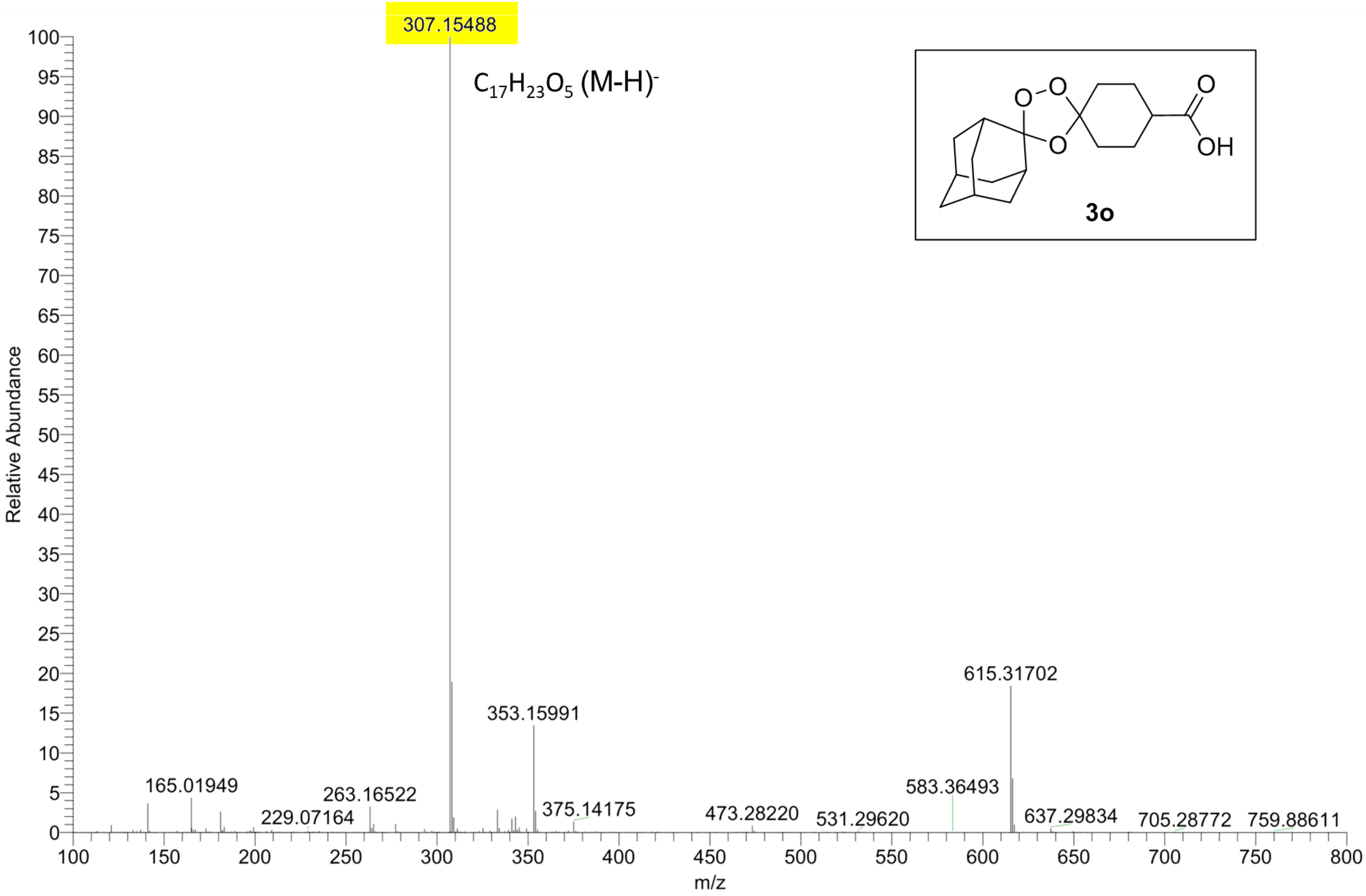

Electrospray ionization mass spectrum in positive-ion mode (HRMS-ESI$^+$) of **compound 2t**

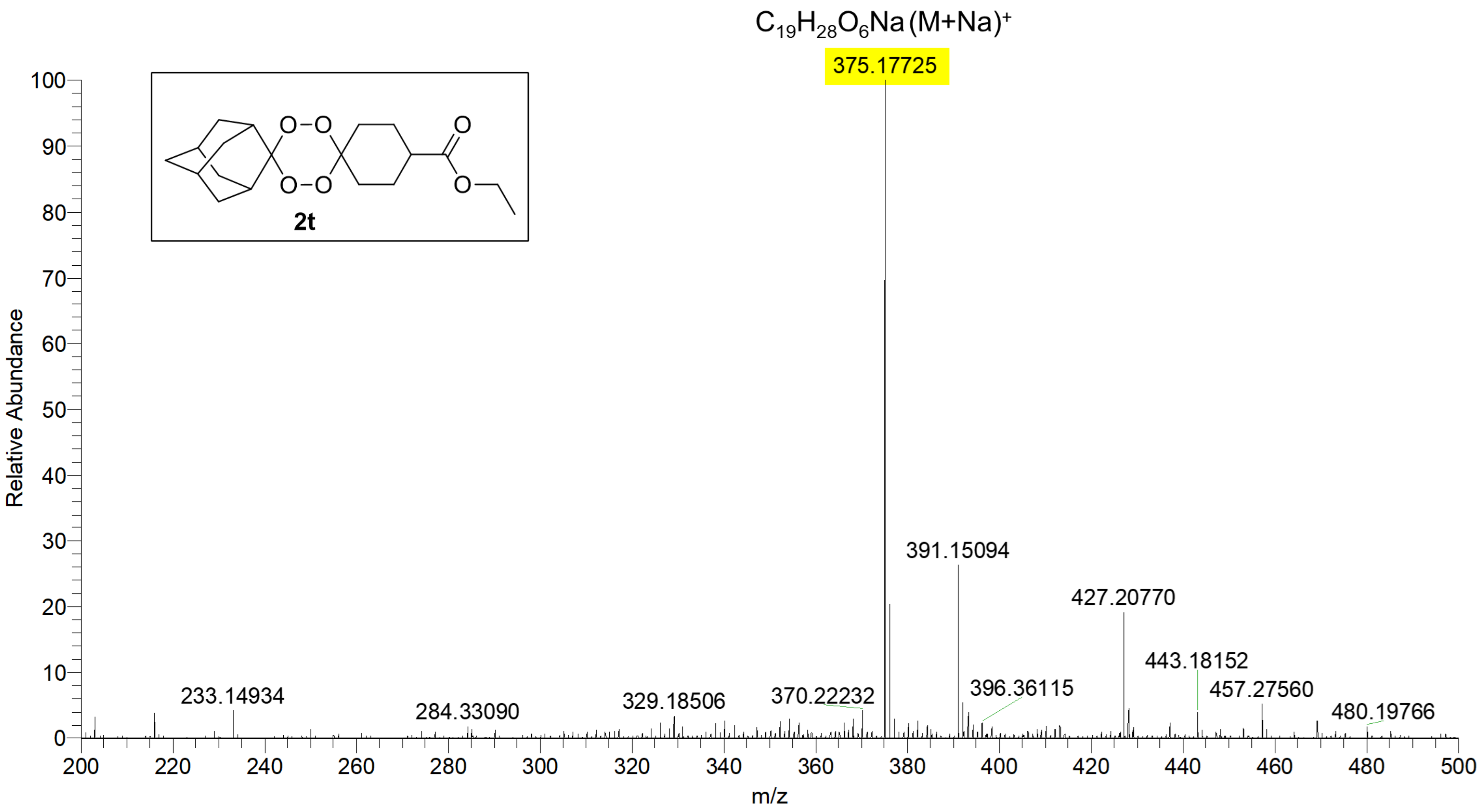

Electrospray ionization mass spectrum in negative-ion mode (HRMS-ESI$^-$) of **compound 3t**

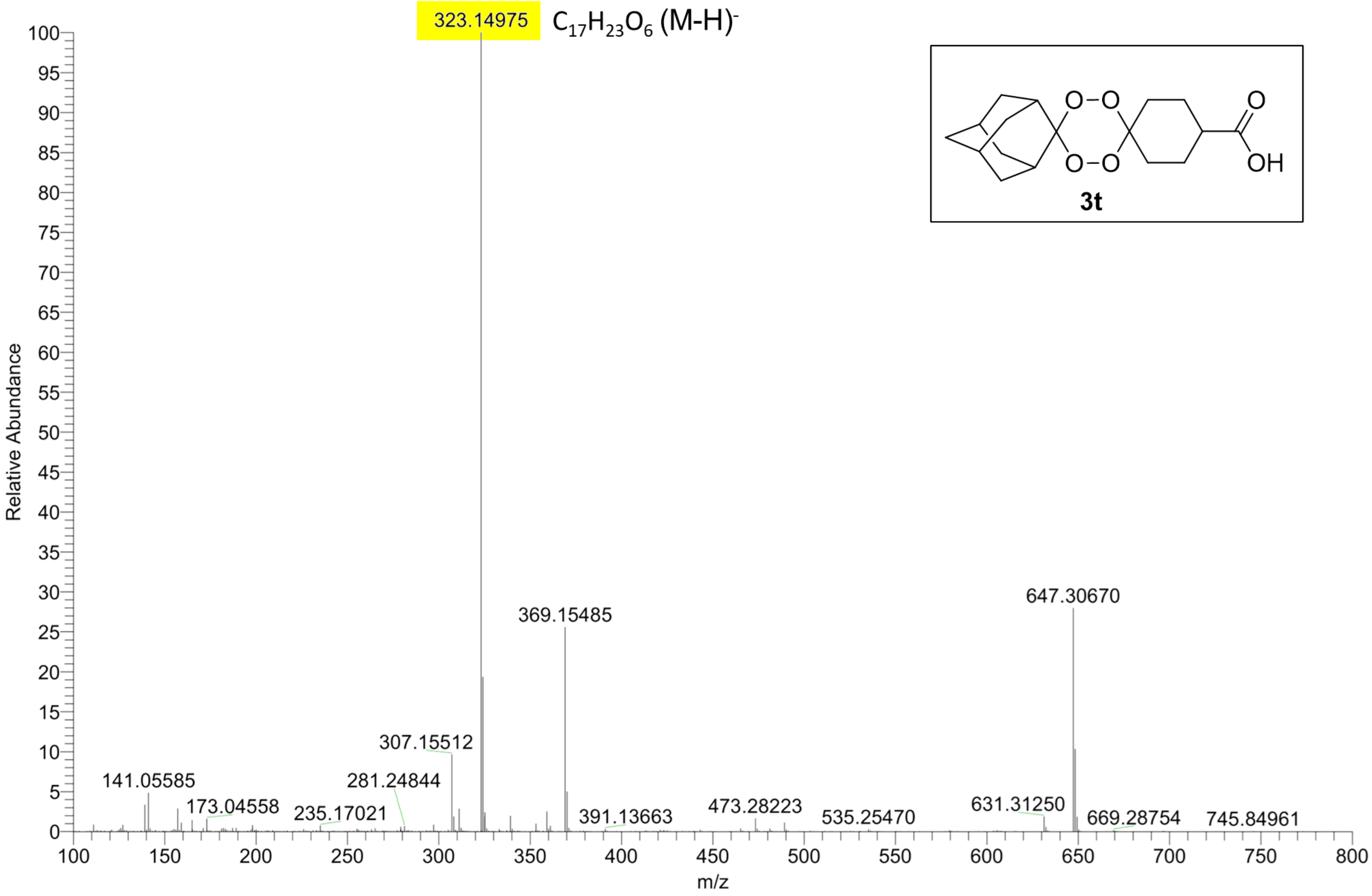

3t

Electrospray ionization mass spectrum in positive-ion mode (HRMS-ESI^{+}) of **compound OZ1**

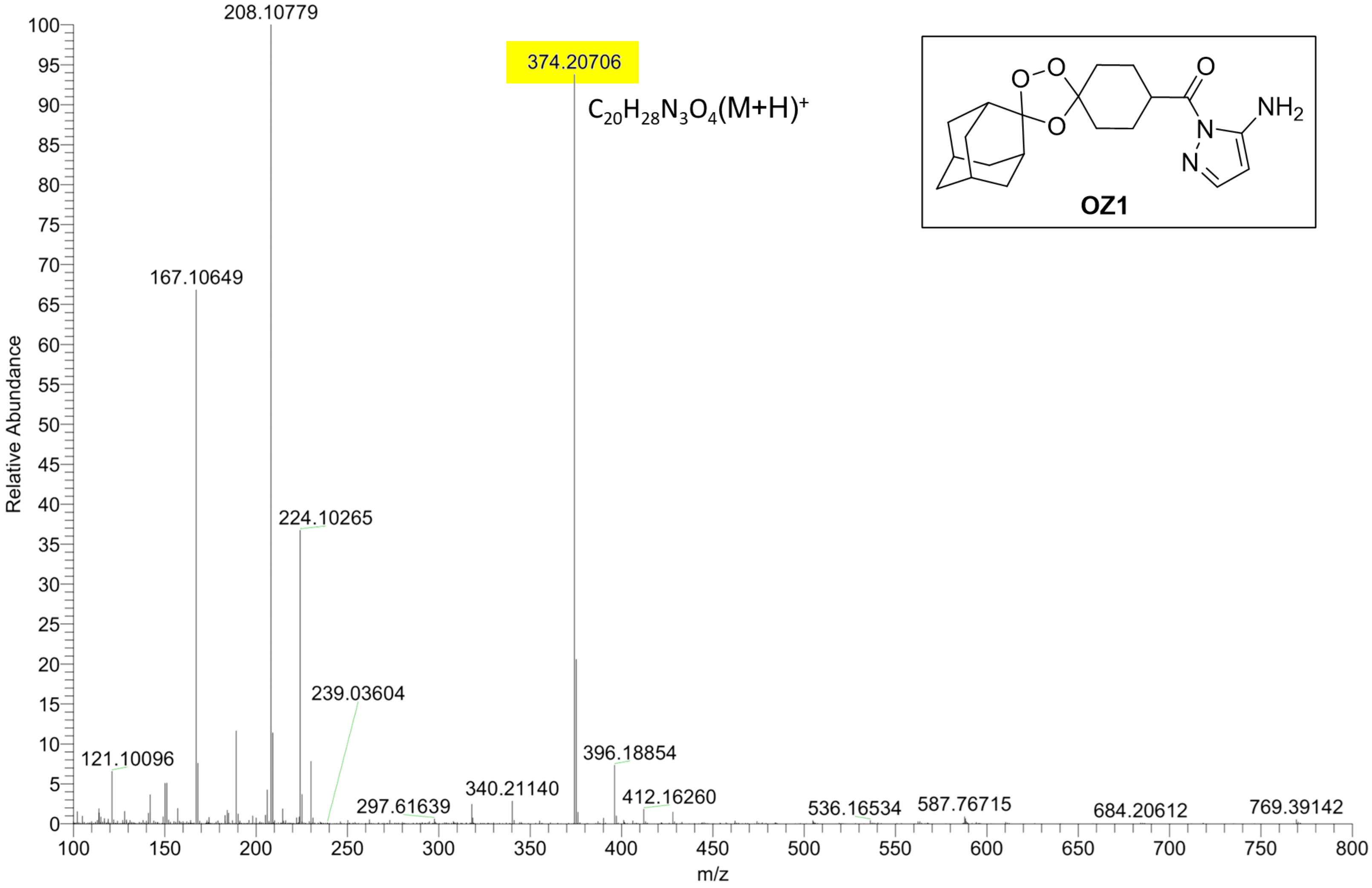

Electrospray ionization mass spectrum in positive-ion mode (HRMS-ESI+) of **compound OZ2**

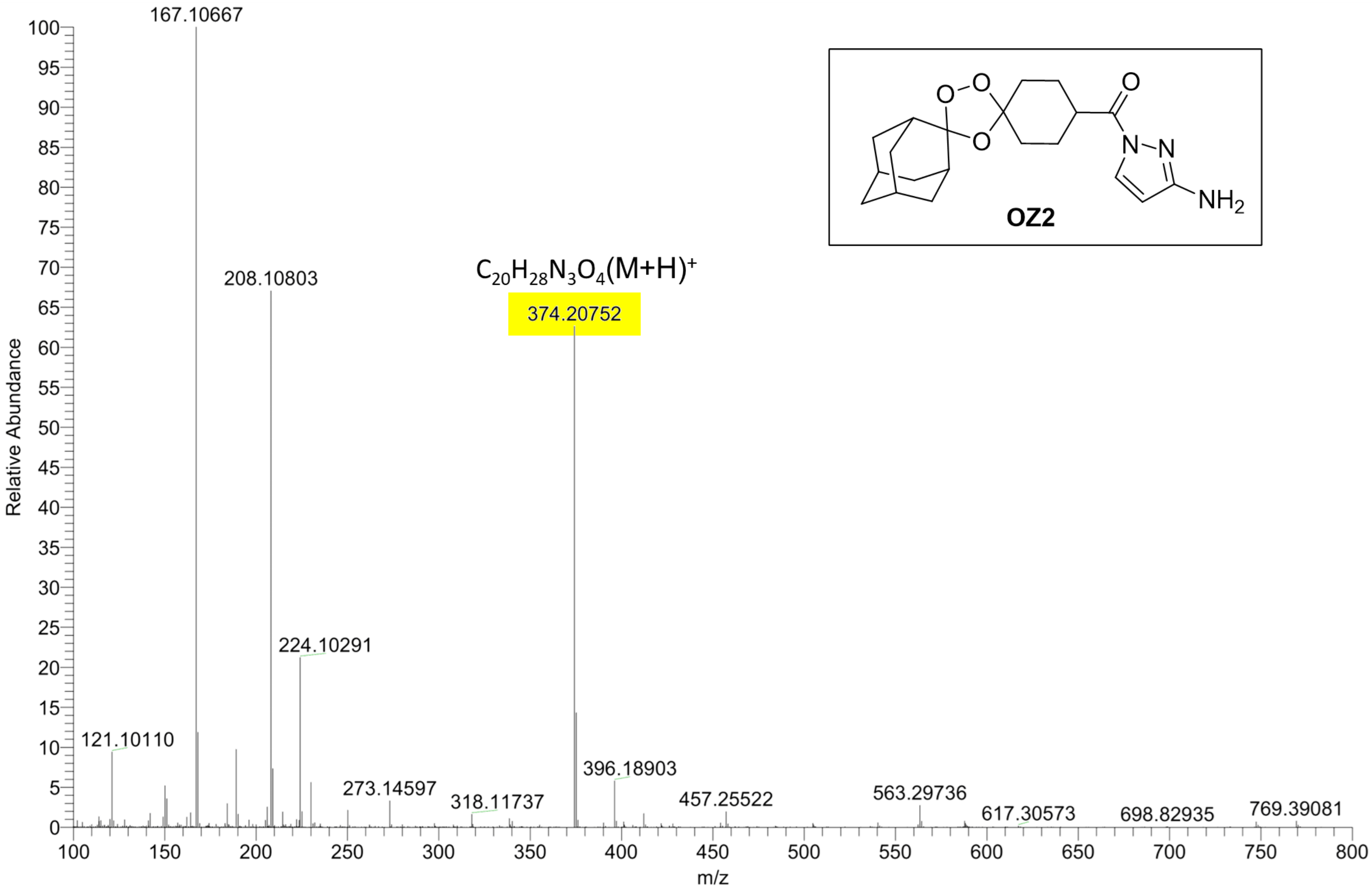

Electrospray ionization mass spectrum in positive-ion mode (HRMS-ESI$^+$) of **compound T1**

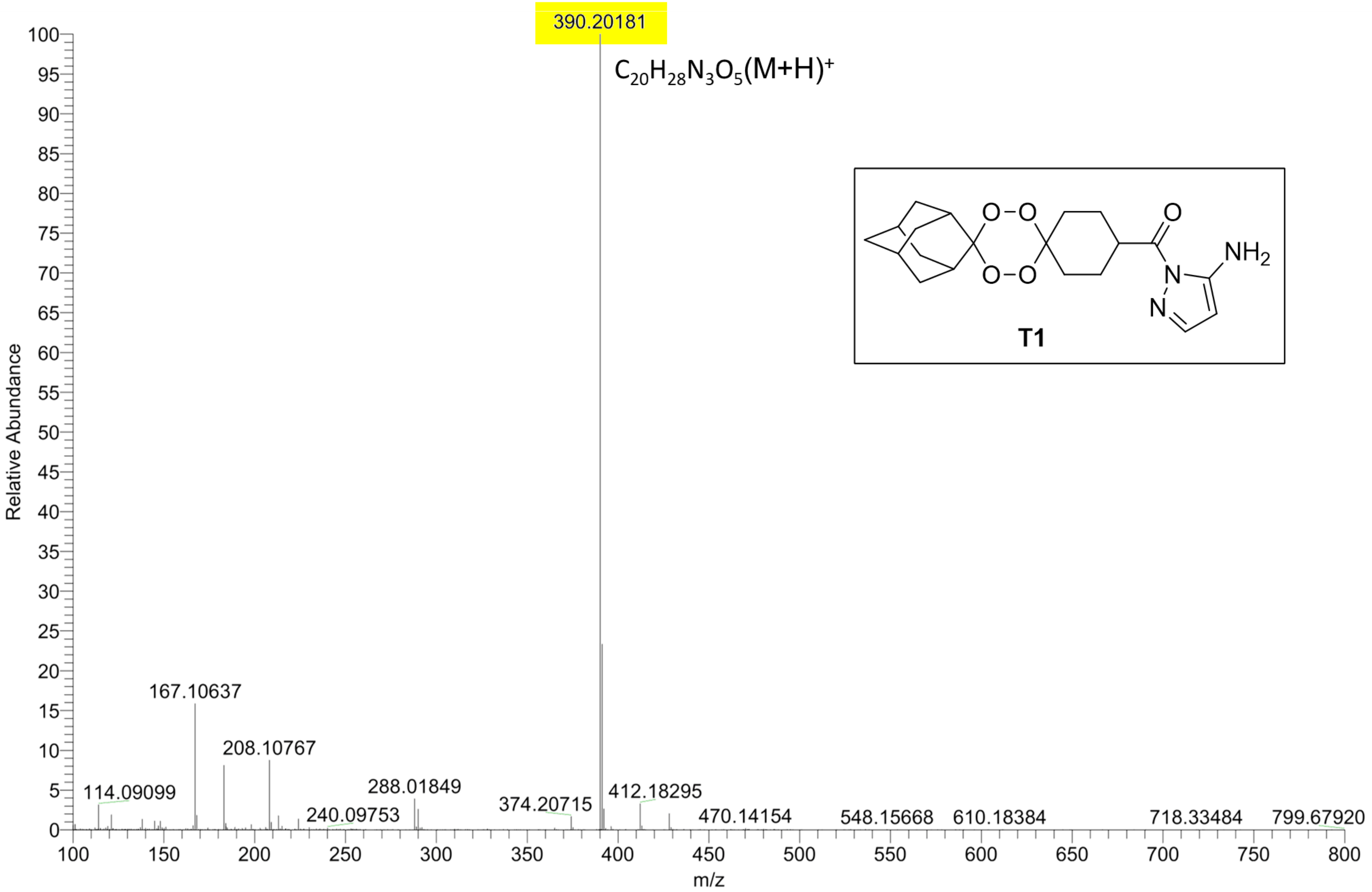

Electrospray ionization mass spectrum in positive-ion mode (HRMS-ESI+) of **compound T2**

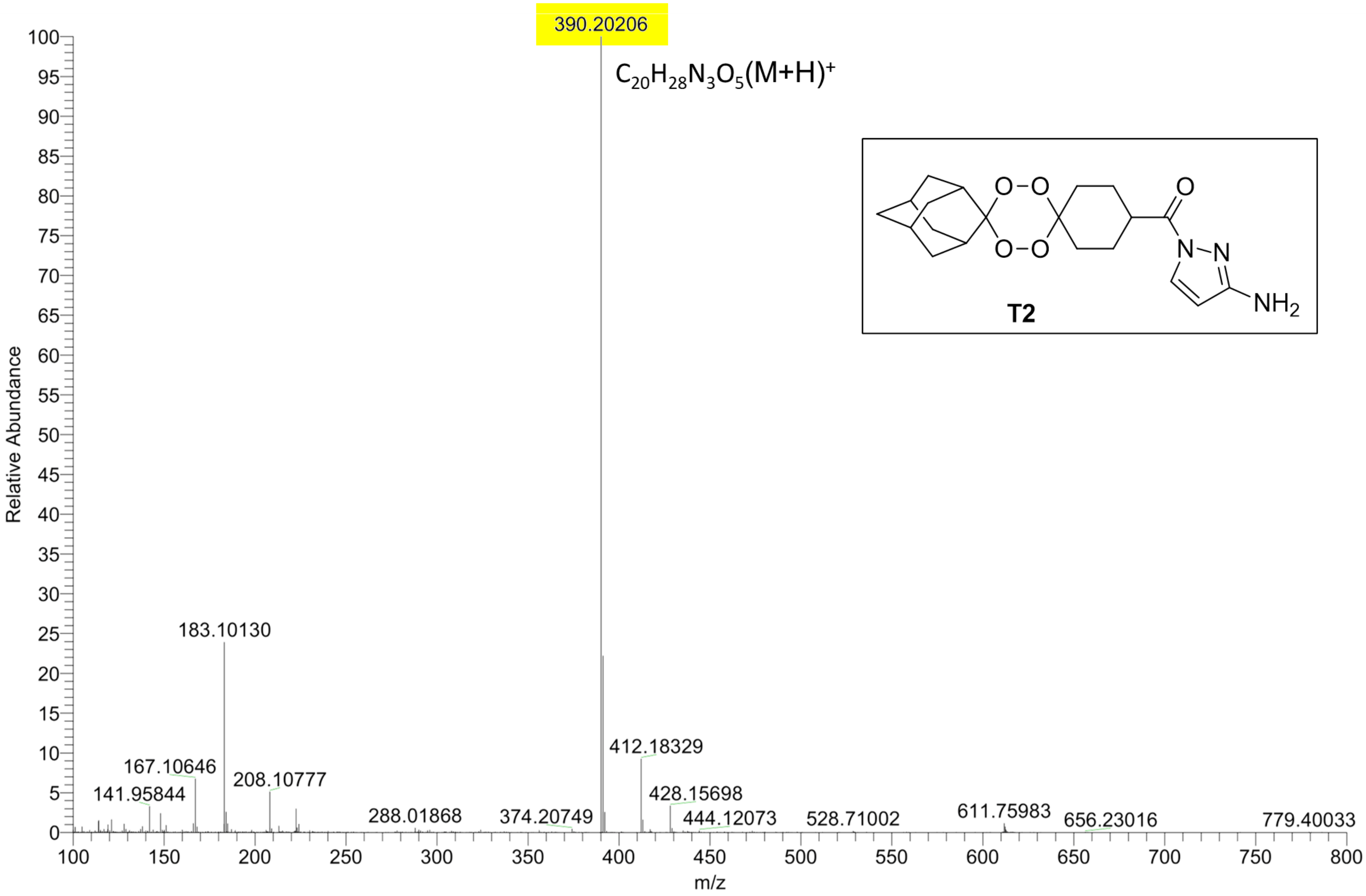

S3. X-ray crystallography

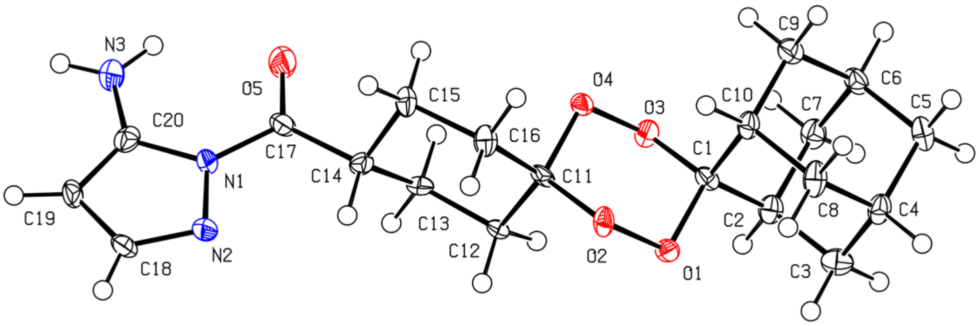

Figure S50. ORTEP drawing of the molecule of (5-amino-*1H*-pyrazol-1-yl) (dispiro[cyclohexane-1,3'-[1,2,4,5]tetraoxane-6',2''-tricyclo[3.3.1.1^{3,7}]decan]-4-yl)-methanone (**T1**). Displacement ellipsoids are drawn at the 50% probability level.

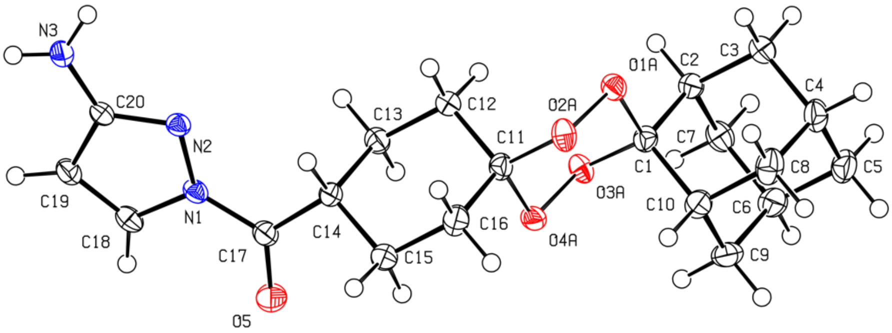

Figure S51. ORTEP drawing of the molecule of (3-amino-*1H*-pyrazol-1-yl) (dispiro[cyclohexane-1,3'-[1,2,4,5]tetraoxane-6',2''-tricyclo[3.3.1.1^{3,7}]decan]-4-yl)-methanone (**T2**). Displacement ellipsoids are drawn at the 50% probability level. The tetraoxane group has minor disorder over two alternate chair conformations; for the sake of clarity, only the major conformation is shown.

CIF files containing supplementary crystallographic data were deposited at the Cambridge Crystallographic Data Centre with references 2151742 (**T1**) and 2151175 (**T2**).
